# Saturation mutagenesis identifies activating and resistance-inducing FGFR kinase domain mutations

**DOI:** 10.1038/s41588-025-02431-8

**Published:** 2025-12-08

**Authors:** Carla Tangermann, Avantika Ghosh, Martin Ziegler, Francesco Facchinetti, Jannis Stappenbeck, Yagmur Oyku Carus Sahin, Marisa Riester, Luise Carmina Viardot, Tobias Zundel, Luc Friboulet, Antoine Hollebecque, José J. Naveja, Angela Wanninger, Maria Elena Hess, Tilman Brummer, Melanie Boerries, Sonja Loges, Yohann Loriot, Anna L. Illert, Sven Diederichs

**Affiliations:** 1 Division of Cancer Research, Department of Thoracic Surgery, Medical Center—University of Freiburg, Faculty of Medicine, University of Freiburg, Freiburg, Germany; 2 German Cancer Consortium (DKTK), partner site Freiburg, a partnership between DKFZ and University Medical Center Freiburg, Freiburg, Germany; 3 Division of Personalized Medical Oncology (A420), German Cancer Research Center (DKFZ), Heidelberg, Germany and DKFZ-Hector Cancer Institute at the University Medical Center Mannheim, Mannheim, Germany; 4 German Cancer Consortium (DKTK), DKFZ core center Heidelberg, Heidelberg, Germany; 5 Université Paris-Saclay, Gustave Roussy, Inserm U981, Villejuif, France; 6 Département d’Innovation Thérapeutique (DITEP), Gustave Roussy, Villejuif, France; 7 Département de Médecine Oncologique, Gustave Roussy, Villejuif, France; 8 3rd Medical Department and University Cancer Center, University Medical Center, Johannes Gutenberg University Mainz, Mainz, Germany; 9 Institute of Molecular Biology (IMB) gGmbH, Mainz, Germany; 10 Institute of Medical Bioinformatics and Systems Medicine, Medical Center—University of Freiburg, Faculty of Medicine, University of Freiburg, Freiburg, Germany; 11 Faculty of Biology, University of Freiburg, Freiburg, Germany; 12 Institute of Molecular Medicine and Cell Research (IMMZ), Medical Center—University of Freiburg, Faculty of Medicine, University of Freiburg, Freiburg, Germany; 13 Internal Medicine III, Hematology and Medical Oncology, School of Medicine, Technical University Munich, Munich, Germany; 14 German Cancer Consortium (DKTK), partner site Munich, a partnership between DKFZ and Technical University Munich, Munich, Germany; 15 Department of Medicine I, Medical Center—University of Freiburg, Faculty of Medicine, University of Freiburg, Freiburg, Germany; 16 Department of Hematology and Medical Oncology, University Medical Center Göttingen (UMG), Göttingen, Germany

**Keywords:** Oncogenes, Targeted therapies, Mutagenesis

## Abstract

Variants of uncertain significance represent the biggest challenge for genomics-based precision oncology. Activated fibroblast growth factor receptors (FGFRs) frequently drive tumorigenesis by different genetic aberrations. However, it remains unknown which of the many point mutations affecting *FGFR1*, *FGFR2*, *FGFR3* or *FGFR4* in cancer are druggable, that is, activating signaling while not mediating FGFR inhibitor resistance. Here we implemented a saturation mutational scanning platform to screen all 11,520 possible point mutations covering the kinase domains of *FGFR1*–*4*. Pooled positive selection screens identified 474 activating and 738 mutations mediating resistance to the FGFR inhibitors pemigatinib and futibatinib, together revealing 301 druggable *FGFR* mutations analogous to a strong PS3/BS3 evidence level. The screens also identified loss-of-function mutations and an association of gain-of-function mutations with hydrophobic changes. The functional screens identified 97% of acquired resistance mutations in clinical trials. Our comprehensive catalog of every druggable mutation in the FGFR kinase domains is readily available for clinical decision support.

## Abstract (web summary)

Saturation mutagenesis screening examines 11,520 point mutations in the kinase domains of *FGFR1*, *FGFR2*, *FGFR3* and *FGFR4*, identifying their activating and resistance properties to the FGFR inhibitors pemigatinib and futibatinib.

## Main

Cancer is frequently driven by genetic aberrations, making functional genomics crucial for interpreting cancer genome data to understand the biological impact of each individual mutation and to clinically match patients with the appropriate targeted therapies. However, except for the few better-studied hotspot mutations, most cancer mutations are variants of uncertain significance (VUSs) whose impact on activation and drug sensitivity is unknown, which considerably limits the therapeutic options despite the availability of corresponding inhibitors.

Building on previous applications of saturation mutagenesis or deep mutational scanning^1–7^, we utilized this approach for the fibroblast growth factor receptor (FGFR) family to evaluate the functional impact of thousands of variants in parallel. FGFRs are central regulators of fetal development and adult tissue homeostasis. They act as driver oncogenes in several cancer types^8^, with an overall prevalence of FGFR aberrations of 7% across all tumor subtypes, but varying between different entities^9,10^. Although *FGFR1* or *FGFR2* amplifications and mutations are frequent in non-small cell lung, breast, endometrial and gastric cancer^11–13^, intrahepatic cholangiocarcinoma is more prone to have *FGFR2* gene fusions^14^. *FGFR3* point mutations and fusions are prevalent in bladder^15^ and cervical cancer^16^ and *FGFR4* amplifications and mutations are observed in head and neck squamous cell carcinoma^17^ and rhabdomyosarcoma^18^.

Like other receptor tyrosine kinases (RTKs), FGFRs possess a conserved intracellular tyrosine kinase domain for phosphorylating tyrosine residues and initiating downstream signaling cascades. The FGFR kinase domain comprises key structural elements, including the activation loop and the ATP-binding pocket^19^, and shares similarities with other RTKs, such as epidermal growth factor receptor (EGFR) and vascular endothelial growth factor receptor (VEGFR)^20^. However, unique structural characteristics of the ATP-binding pocket within the FGFR split kinase domain confer specificity for FGFR-selective therapies while minimizing off-target effects^21^.

To target cancer cells driven by aberrantly activated FGFR, FGFR inhibitors (FGFRis) have been developed. Pemigatinib (INCB054828) and futibatinib (TAS-120) have obtained approval from the US Food and Drug Administration (FDA) for treatment of metastatic cholangiocarcinoma with an *FGFR2* fusion or other rearrangement^22,23^. Erdafitinib has received FDA approval for locally advanced or metastatic urothelial carcinoma with FGFR3 or FGFR2 aberrations^24^. These approvals highlight the clinical relevance of FGFR-targeted therapies in specific cancer subtypes. However, the approval of pemigatinib and futibatinib has been granted only for FGFR rearrangements and not for point mutations, which are nevertheless frequently found in cancer and could also be targeted with these inhibitors. Erdafitinib is the only FGFRi approved for bladder cancer harboring any of only four point mutations in the FGFR3 extracellular domain (Arg248Cys, Ser249Cys, Gly370Cys and Tyr373Cys). The many functionally uncharacterized point mutations are particularly challenging. These are not considered as actionable markers for FGFRi treatment, because they often associate with resistance^25–28^.

To exploit these mutations for precision medicine, a detailed understanding of each FGFR kinase mutation and its effect on activation and resistance is essential. This study provides a saturation mutational scanning of all 11,520 possible point mutations in the kinase domains of *FGFR1*, *FGFR2*, *FGFR3* and *FGFR4*. In positive selection screens, we have compiled a comprehensive catalog defining the activation and resistance, and thus actionability and clinical relevance, of each *FGFR* kinase mutation for pemigatinib and futibatinib.

## Results

### Saturation mutagenesis for *FGFR* activation and drug resistance

**Fig. 1:**
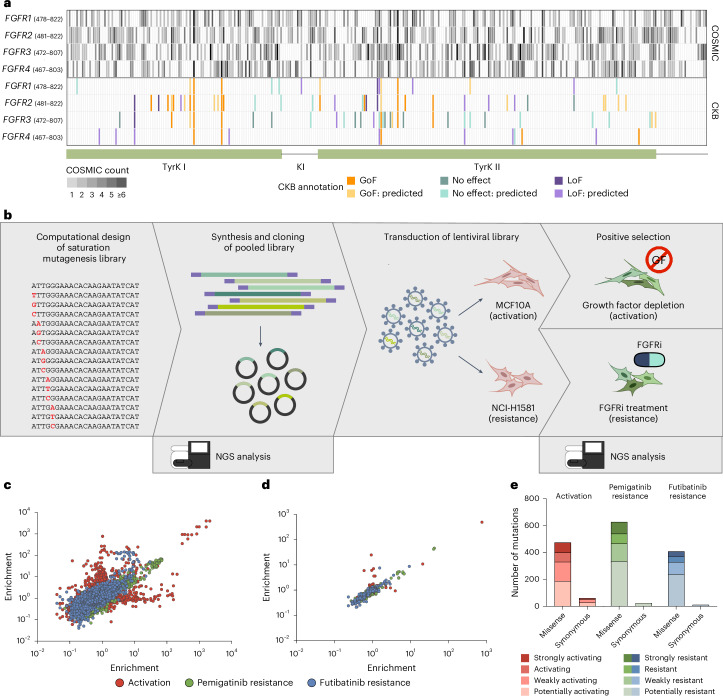
Saturation mutational scanning to identify activating and resistance-inducing *FGFR* mutations. **a**, Point mutations in the *FGFR1*–*4* kinase domains until their carboxy-termini reported in COSMIC and CKB. Top: each value representing the number of COSMIC hits harboring a mutation at this amino acid position. Bottom: the corresponding information for the same amino acid in CKB for each *FGFR*. **b**, Overview of the saturation mutational scanning workflow. Saturation mutagenesis libraries were designed computationally and ordered as pooled oligonucleotide libraries. Oligonucleotides of each pool were cloned into a plasmid library which was analyzed via NGS to determine coverage for each mutation. Lentiviral libraries derived from these plasmid libraries were used to transduce MCF10A or NCI-H1581 cells. MCF10A cells were positively selected on growth factor depletion (no EGF, no insulin and reduced horse serum) for up to 3 weeks. NCI-H1581 cells were treated with pemigatinib or futibatinib for 8 d to positively select for resistance-conferring mutations. After selection, gDNA was analyzed via NGS. **c**, *FGFR1* correlation of replicates for activation and resistance screens. All combinations of the replicates for activation (*n* = 4) or resistance (*n* = 3) are depicted. **d**, Median enrichment correlation between different nucleotide mutations giving rise to the same amino acid change in *FGFR1*. **e**, Comparison of activating or resistance-conferring missense and synonymous mutations and their categorization according to the effect strength for *FGFR1*–*4*. TyrK, tyrosine kinase domain. Source data

*FGFR* point mutations frequently occur in tumor samples. Our pan-cancer analysis of the COSMIC database of somatic mutations in cancer found a total of 1,749 mutations distributed across the entire kinase domains of *FGFR1*–*4* (Fig. 1a). However, as most mutations are of uncertain significance, physicians cannot exploit most point mutations for clinical decision-making even though FGFRi drugs are available. In the Clinical Knowledge Base (CKB), information on the protein effects is available for only a minute number of mutations (Fig. 1a), for only 1.3% of all possible point mutations in the kinase domains.

**Extended Data Fig. 1:**
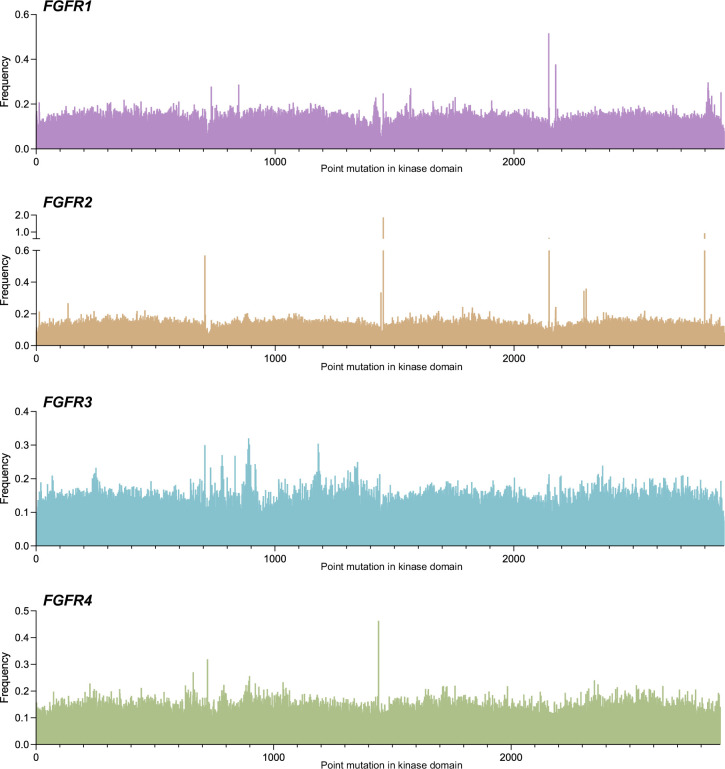
Coverage of *FGFR* plasmid libraries. Presence and frequency of each point mutation in the plasmid libraries covering the kinase domains of *FGFR1, FGFR2, FGFR3* and *FGFR4* determined by NGS. Data represent the median of n = 3 independent replicates. Source data

**Extended Data Fig. 2:**
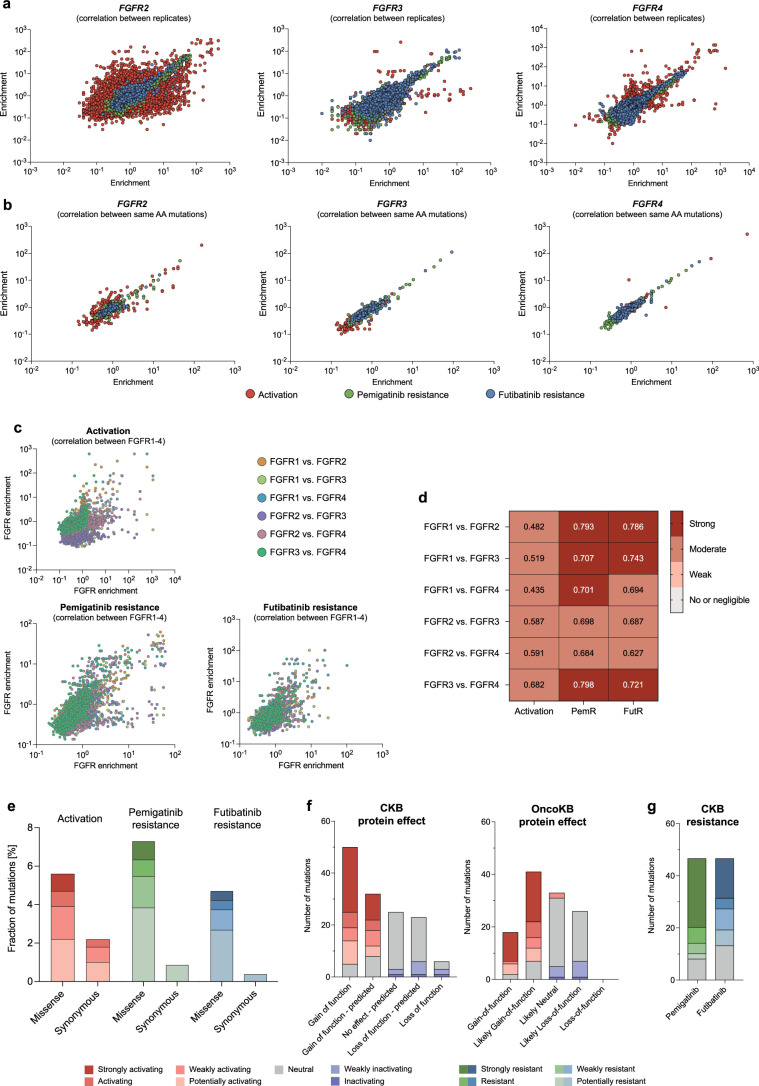
Quality controls of the screen and comparison between FGFR1-4. **a**, Correlation of replicates for activation (n = 4) and resistance (n = 3) for *FGFR2*, *FGFR3* and *FGFR4*. All combinations of the different replicates are depicted together. **b**, Median enrichment correlation between different nucleotide mutations giving rise to the same amino acid change in *FGFR2, FGFR3* and *FGFR4*. **c-d**, Correlation of median enrichments between *FGFR1-4* for each screen condition (activation, pemigatinib and futibatinib resistance). All six pairwise combinations of the different *FGFR*s are depicted together (c) and Spearman correlation coefficients indicate strong to very strong correlations between the different *FGFR*s for each screen condition **(d)**. **e**, Fraction of activating or resistance-conferring missense and synonymous mutations and their categorization according to the effect strength for *FGFR1-4* normalized to the total number of missense or synonymous mutations included in the screen. **f**, Comparison of the functional effect determined in the activation screen and available information on mutation classification according to the two databases CKB and OncoKB for FGFR1-4. **g**, Comparison of the functional effect determined in both resistance screens and available information on drug resistance in CKB. Source data

The large number of different mutations already found in tumors provided the rationale for saturation mutational scanning covering all possible *FGFR* point mutations, thus including mutations appearing in patients with cancer in the future. To gain functional information about activating and resistance properties, we designed a saturation mutagenesis expression library on the nucleotide level for the kinase domains of *FGFR1*, *FGFR2*, *FGFR3* and *FGFR4*, comprising 11,520 single nucleotide substitutions resulting in 7,524 amino acid substitutions (Fig. 1b). Pooled oligonucleotide libraries were synthesized and cloned into lentiviral expression plasmids (Extended Data Fig. 1). To identify activating mutations, we used the growth factor-dependent cell line MCF10A^29,30^. Transduction of the mutation libraries led to a stable expression of the respective *FGFR* harboring one distinct point mutation. Under growth factor depletion, cells were positively selected for mutations that drove cell proliferation. To identify resistance mutations, the FGFRi-sensitive cell line NCI-H1581 (*FGFR1* amplified lung cancer, but dependent only on *FGFR2* signaling according to DepMap) was transduced and treated with either 20 nM pemigatinib or 10 nM futibatinib, two FGFRis in clinical use. These concentrations, based on their half-maximal inhibitory concentration (IC_50_) values, matched recently published inhibitor concentrations^28^ and, importantly, were equal or well below clinically achievable serum concentrations^31^. For all screens, genomic DNA (gDNA) of positively selected cells was harvested and analyzed via next-generation sequencing (NGS) to determine mutations leading to activation, pemigatinib resistance or futibatinib resistance (Fig. 1b). Multiple quality controls, such as correlations^32^ or comparisons between missense and synonymous mutations^33^, confirmed the validity of the data (Fig. 1c–e, Extended Data Fig. 2a–e, Supplementary Note and Supplementary Tables 1 and 2).

Furthermore, we compared the screen results with information about gain-of-function (GoF), loss-of-function (LoF), neutral and resistance mutations in the CKB^34^ and OncoKB^35^ and found a strong overlap (Extended Data Fig. 2f,g). Following the American College of Medical Genetics and Genomics (ACMG)/Association for Molecular Pathology (AMP) clinical variant interpretation guidelines^36^, we calculated an odds path of 45.4/52.0 for pathogenic and 0.010/0.015 for benign variants as listed in the CKB and OncoKB, respectively, resulting in a strong PS3/BS3 evidence level (Supplementary Table 3).

Overall, these quality control analyses demonstrated that we established a reliable and robust screening system for activating and resistance-mediating *FGFR* mutations.

### Hundreds of variants in the kinase domains activate FGFR

**Fig. 2:**
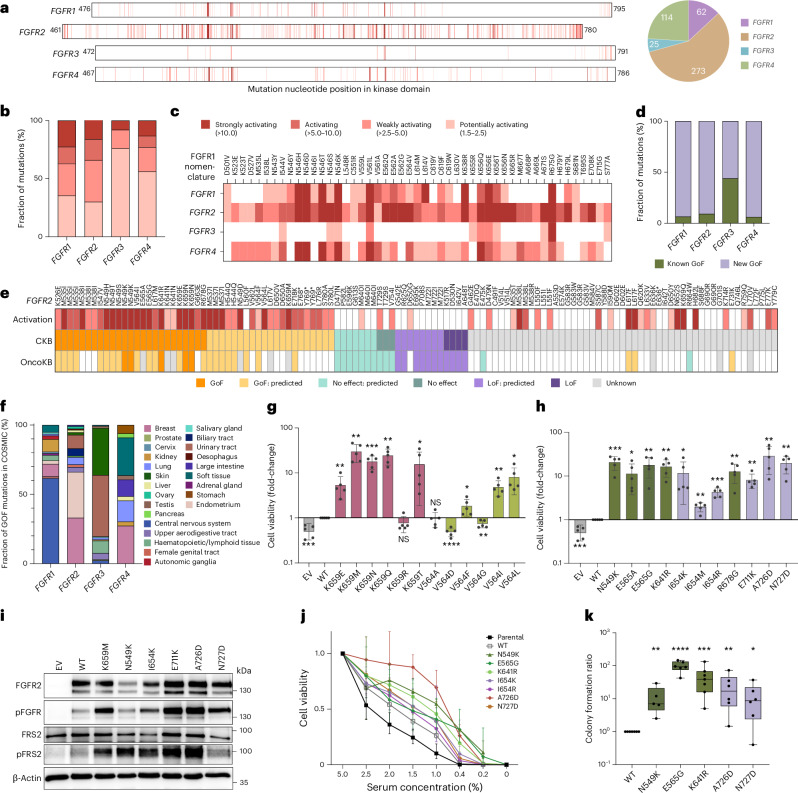
Detection and validation of activating *FGFR* mutations. **a**, Enrichment scores for missense mutations sorted according to their position in the kinase domains of *FGFR1*–*4*. Each value represents the median of four independent biological replicates. The intensity of the red color indicates the strength of the enrichment detected. The pie chart presents the absolute number of activating mutations (≥1.5-fold enrichment in at least 3 of 4 replicates) per *FGFR*. **b**, Overview of the distribution of activating mutations according to the effect size for *FGFR1*–*4*. **c**, Amino acid mutations with conserved activation in at least two FGFRs. The intensity of the red color indicates the median activation strength. For nucleotide mutations leading to the same amino acid change, the value represents the median of all mutations coding for the same amino acid. Mutations are listed according to their nomenclature in *FGFR1*. **d**, Overview of the proportion of activating mutations that are already reported in the CKB or OncoKB (‘known’) or were newly identified in this screen. **e**, Comparison of protein effect classifications for *FGFR2* mutations by the CKB or OncoKB and their detected median activation properties. Amino acid changes listed more than once arise from different nucleotide mutations independently tested. **f**, Distribution of tumor subtypes (COSMIC) harboring CKB GoF mutations for *FGFR1*–*4*. **g**,**h**, Validation of activating *FGFR2* mutations in codons Lys659 (pink) and Val564 (light-green) (**g**) and of known (dark-green) and new (lilac) GoF mutations (**h**) in MCF10A cell viability assays. Empty vector (EV) and FGFR2 wildtype (WT) constructs served as controls (gray). The data are presented as the mean of the fold-change compared to the WT ± s.d. of *n* = 5 biological replicates. *P* values are calculated for each mutation compared to WT using two-tailed Student’s *t*-tests. **i**, Stable overexpression of six different *FGFR2* mutants in MCF10A cells leading to increased FGFR and FRS2 phosphorylation compared to WT overexpression in western blot analysis. Lys659Met and Asn549Lys are well-known GoF mutations and Ile654Lys, Glu711Lys, Ala726Asp and Asn727Asp are newly identified in the activation screen. A representative blot for *n* = 3 biological replicates with similar results is shown. **j**, NIH-3T3 low-serum proliferation assay. For each mutation and serum concentration, the geometric mean ± s.d. of *n* = 3 for Asn549Lys, *n* = 4 for parental, *n* = 5 for WT, Lys641Arg, Ile654Lys, Ile654Arg and Asn727Asp, and *n* = 6 for Glu565Gly and Ala726Asp biological replicates is plotted. **k**, Quantification of NIH-3T3 colony formation soft agar assay with log(transformation) of ratios normalized to WT from *n* = 5 for Asn549Lys, *n* = 6 for Glu565Gly, Ala726Asp and Asn727Asp to *n* = 7 for Lys641Arg biological replicates. The center line is the median, the interquartile range is the 25th to 75th percentiles and the whiskers go down to the smallest value and up to the largest). *P* values are calculated for each mutation compared to WT using Student’s *t*-tests on log(transformed) ratios. *P < 0.05, **P < 0.01, ***P < 0.001, ****P < 0.0001. NS, not significant. Source data

The screen for growth factor independence reflecting *FGFR* activation identified 474 activating *FGFR1*–*4* kinase domain missense mutations (Fig. 2a), which corresponded to 5.6% of all 8,407 tested missense mutations. Mutations were classified as activating if the level of enrichment was higher than or equal to 1.5 in 3 out of 4 independent biological replicates. Most activating mutations occurred in *FGFR2* (58%) and the fewest in *FGFR3* (5%). We categorized activating mutations according to their median enrichment into four categories: potentially activating (1.5-fold to 2.5-fold), weakly activating (>2.5-fold to 5-fold), activating (>5-fold to 10-fold) or strongly activating (>10-fold) (Fig. 2b).

**Extended Data Fig. 3:**
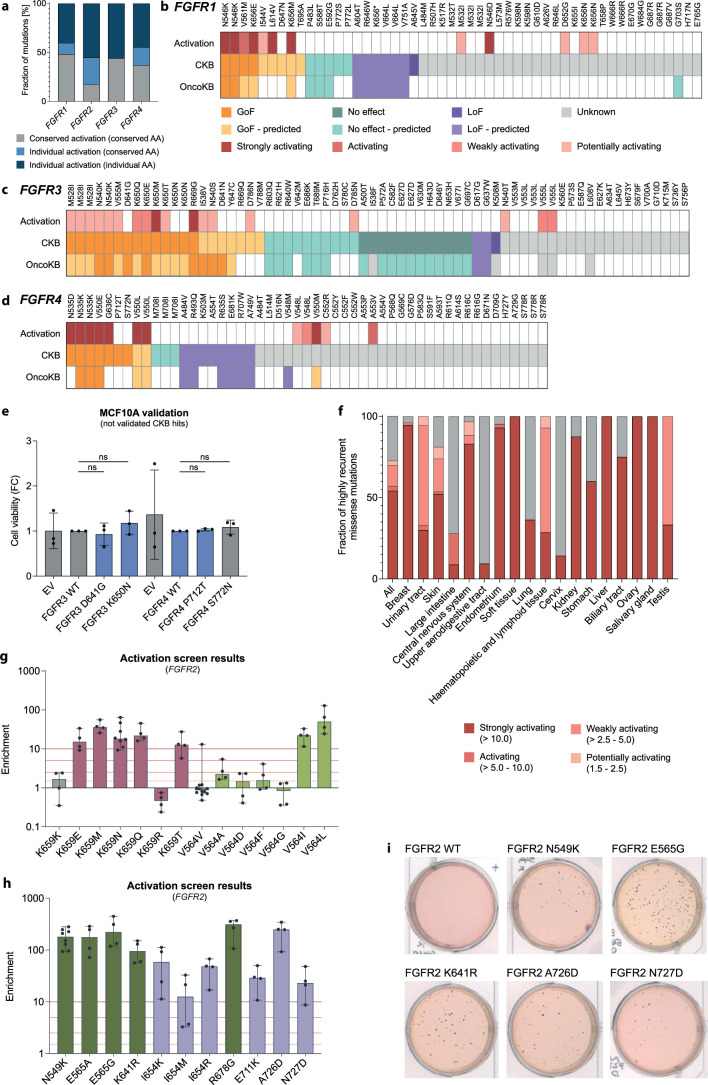
Individual and conserved activating *FGFR* mutations, their reporting in CKB and their tumor distribution in COSMIC. **a**, Fraction of activating mutations affecting conserved and non-conserved amino acids in FGFR1-4. Conserved activation was assigned when the mutation affected a residue conserved in all four *FGFR*s and the same mutation was activating in two or more *FGFR*s. An individual activation was only observed in the respective *FGFR* but may affect conserved or individual amino acid mutations. **b-d**, Comparison of CKB/OncoKB protein effect classification for *FGFR1* (**b**), *FGFR3* (**c**) and *FGFR4* (**d**) missense variants and their median activating properties detected in the activation screen (n = 4). Amino acid mutations listed more than once arise from different nucleotide mutations resulting in the same amino acid change. **e**, Validation of the lack of an activation effect for mutations reported as GoF mutations in CKB, but neutral in the screen (blue). *FGFR3* and *FGFR4* wildtype (WT) and empty vector (EV) constructs served as controls (grey). The data are presented as the mean of the fold change (n≥3) in MCF10A cell viability compared to the EV ± standard deviation of biological replicates. *P* values are calculated for each mutation compared to the respective WT using a two-tailed Student’s t-test. **f**, Primary sites of tumors (with at least three mutations) for mutations in *FGFR1-4* reported at least five times in COSMIC (highly recurrent) and their categorization according to enrichment in the activation screen. The first bar represents all activating mutations regardless of their entity, and the order of the different entities is based on the total number of mutations present in this entity, with the most occurring in breast and the least in testis. **g**, Results of the activation screen for mutations in the FGFR2 amino acids K659 (pink) and V564 (light green), with the synonymous mutations for each amino acid colored in gray. The four red colored lines represent the thresholds for effect strength classification. The data are presented as the median of n = 4 biological replicates with 95% CI (mutations with n > 4 replicates result from different nucleotide mutations that lead to the same amino acid change). **h**, Results of the activation screen for known (dark green) and new (lilac) *FGFR2* GoF mutations. Data represent the median of n = 4 independent replicates with 95% CI (mutations with n > 4 replicates result from different nucleotide mutations that lead to the same amino acid change). **i**, Pictures of NIH-3T3 colony formation soft agar assay with nitroblue tetrazolium chloride stained colonies after 14 days of incubation. Source data

The four FGFR kinase domains are highly conserved with 62% of the amino acids (and 54% of the amino acid changes due to point mutations) identical in all four FGFRs. Eight mutations were activating in all four FGFRs (*FGFR1* nomenclature): Ile544Val, Asn546Ser, Asn546Lys, Val561Leu, Lys656Gln, Lys656Glu, Lys656Thr and Arg675Gly (Fig. 2c). Although these were mainly hotspot mutations^37,38^, their activating effect was unknown for 41% because it had been described only in other FGFRs. We further compared amino acids conserved in all four FGFRs to overlapping activation in at least two FGFRs. However, a majority of activating mutations were activating only in one FGFR (Extended Data Fig. 3a).

**Extended Data Fig. 4:**
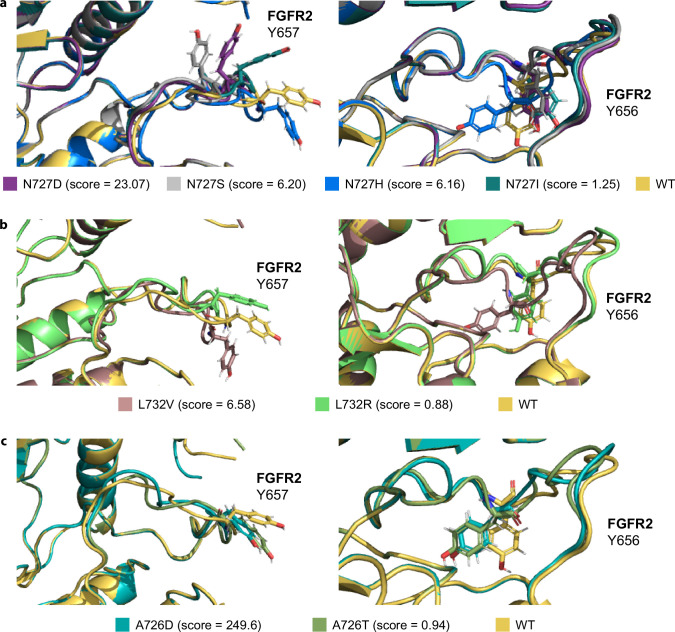
Structural modeling of C-terminal FGFR2 mutations. **a-c**, The effect on the protein structure of selected C-terminal mutations that were classified as activating or neutral in the screen (and affect the same amino acid) was predicted using AlphaFold2. The focus is on the orientation of the critical autophosphorylation sites Y656 and Y657 for each mutation. For N727 **(a)**, three activating mutations and one neutral mutation are visualized with a visible change in orientation for all activating mutations for Y657, while for Y656, only N727H is oriented differently than WT/neutral. For L732 **(b)**, one activating and one neutral mutation is visualized with a visible change in orientation for the activating mutation for Y657 and Y656. For A726 **(c)**, one activating and one neutral mutation is visualized with no visible change for both residues. Source data

Further analyses of the dataset included comparison to the CKB and OncoKB databases, tumor subtypes harboring activating mutations and experimental validation of individually cloned mutations in two cell line models, structural modeling and analysis of functional motifs^39^ or autophosphorylation sites^40,41^ (Fig. 2d–j, Extended Data Figs. 3b–i and 4a–c, Supplementary Note and Supplementary Table 4).

In conclusion, we identified hundreds of activating kinase domain mutations in *FGFR1*–*4*. The respective somatic mutations in cancer covered a broad spectrum of tumor subtypes. Validations in two different cell lines indicated a high level of reproducibility, robustness and universal validity of the mutational scanning data.

### Dual or selective resistance mutations to pemigatinib or futibatinib

**Fig. 3:**
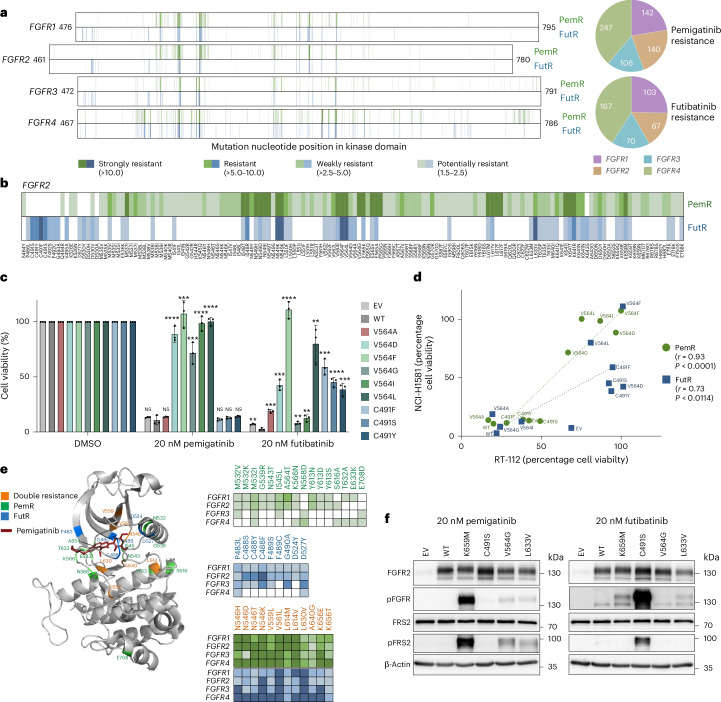
Determination of *FGFR* mutations mediating resistance to pemigatinib and futibatinib. **a**, Heatmap with enrichment scores (median of *n* = 3) for resistance to pemigatinib (PemR, green) or futibatinib (FutR, blue) for missense mutations sorted according to their position in the kinase domains. The color intensity indicates the strength of the enrichment. The pie chart presents the absolute number of resistance mutations per *FGFR* for each inhibitor. Mutations were classified as resistant if an enrichment ≥1.5 was observed in 2 out of 3 independent biological replicates. **b**, Heatmap depicting all *FGFR2* mutations with resistance phenotypes to pemigatinib, futibatinib or both inhibitors (legend in **a**). **c**, Validation of *FGFR2* mutations conferring similar or differential resistance to pemigatinib and futibatinib. Mean cell viability of NCI-H1581 cells is presented normalized to dimethyl sulfoxide (DMSO) ± s.d. of *n* = 3 biological replicates. *P* values are calculated for each mutation compared to WT using two-tailed Student’s *t*-tests. **d**, Comparison of RT-112 and NCI-H1581 resistance validation (mean cell viability normalized to DMSO in the respective cell line). The dotted lines and *r* values reflect Pearson’s correlations between both cell lines for the two inhibitors (PemR green, FutR blue). **e**, *FGFR1* WT kinase domain structure (Protein Data Bank (PDB) accession no. 7WCL) with bound pemigatinib (red), with colored amino acids according to their resistance phenotype to pemigatinib (green), futibatinib (blue) or both (orange) in *FGFR1*–*4*. The colors in the heatmaps depict the strength of the resistance enrichment in *FGFR1*–*4* (legend in **a**). **f**, Stable overexpression of four different resistance-conferring *FGFR2* mutations in NCI-H1581 cells leading to increased FGFR phosphorylation compared to WT, after treatment with either 20 nM pemigatinib (left) or 20 nM futibatinib (right). Lys659Met is a well-known resistance mutation (double resistance) whereas Cys491Ser (only FutR), Val564Gly (only PemR) and Leu633Val (double resistance) are newly identified in the resistance screens. A representative blot for *n* = 3 biological replicates with similar results is shown. **P < 0.01, ***P < 0.001, ****P < 0.0001. Source data

**Extended Data Fig. 5:**
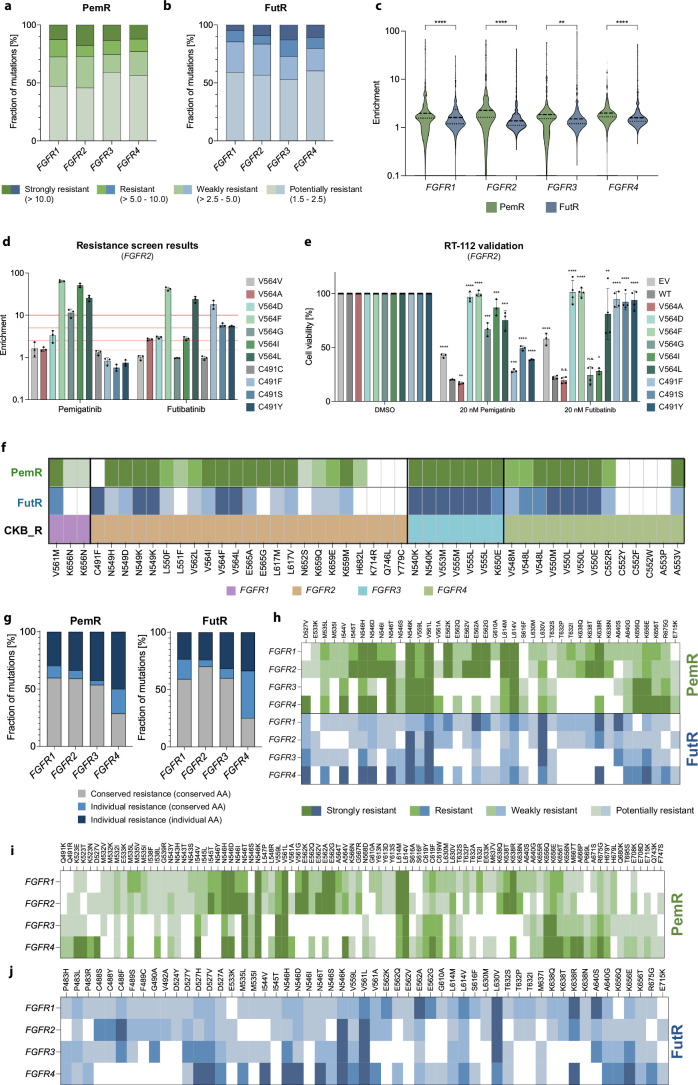
Resistance mediating mutations identified in the saturation screen and comparison with CKB and between different *FGFR*s. **a-b**, Classification and distribution of mutations according to their effect strength for pemigatinib (green, **a**) and futibatinib (blue, **b**) resistance screens in *FGFR1-4*. **c**, Violin plot representing the resistance enrichment levels for pemigatinib (PemR) and futibatinib (FutR) for the full screen results (n = 3). *P* values for each *FGFR* are calculated using a nonparametric two-tailed Mann-Whitney *U* test comparing two unpaired groups. **d**, Median results (n = 3, 95% CI) of both resistance screens affecting the FGFR2 amino acids V564 and C491. The four red colored lines represent the thresholds for effect strength classification. **e**, Validation of *FGFR2* mutations conferring similar or differential resistance to pemigatinib or futibatinib in RT-112 bladder cancer cells. The data are presented as the mean of cell viability normalized to DMSO ± standard deviation of n = 3 for pemigatinib and n = 4 for futibatinib biological replicates. *P* values are calculated for each mutation compared to WT using a two-tailed Student’s t-test. **f**, Comparison of CKB resistance classification for *FGFR1***-***4* missense variants and their impact on resistance to pemigatinib (green) and futibatinib (blue) depicted as median of n = 3 independent replicates (see legend in panel **a**). **g**, Fraction of resistance mutations affecting conserved and non-conserved amino acids. Conserved resistance was assigned when the mutation affected a residue conserved in all four *FGFR*s and the same mutation was mediating resistance in two or more FGFRs. An individual resistance was only observed in the respective *FGFR* but may affect conserved or individual amino acid mutations. **h-j**, Amino acid mutations with conserved PemR and FutR in at least two FGFRs (**h**), with conserved PemR in at least two FGFRs (**i**) or with conserved FutR in at least two FGFRs (**j**). The intensity of the respective color indicates the resistance strength (n = 3). **f,h-j**, Amino acid mutations listed more than once arise from different nucleotide mutations that lead to the same amino acid change. In this case, the color represents the median of all samples encoding for the same amino acid. Mutations are named according to their nomenclature in FGFR1. Source data

Few point mutations in the kinase domains are known to induce FGFRi resistance^42,43^. To comprehensively identify mutations mediating resistance to the FDA-approved inhibitors pemigatinib (designed to target *FGFR1*–*3*) and futibatinib (designed to target *FGFR1*–*4*), we established a second pooled high-throughput system in the FGFRi-sensitive lung cancer cell line NCI-H1581. The cells were transduced with the FGFR mutation pools and resistance mutations were positively selected for by treating with the FGFRis (Fig. 1b). Resistance mutations were classified depending on their enrichment (Extended Data Fig. 5a,b). Screening 11,520 mutations in the *FGFR1*–*4* kinase domains detected 635 pemigatinib resistance (PemR) and 407 futibatinib resistance (FutR) mutations (Fig. 3a and Extended Data Fig. 5c). Resistance mutations were more evenly distributed between the *FGFR*s than activating mutations, with *FGFR4* harboring most resistance mutations (40%).

For *FGFR2*, 32% of the resistance mutations conferred dual resistance to both inhibitors, whereas 58% were specific to pemigatinib and only 10% to futibatinib—clustering at amino acids Cys491, the covalent binding site of the irreversible inhibitor futibatinib^28^, Phe492 and Asp530 (Fig. 3b).

**Extended Data Fig. 6:**
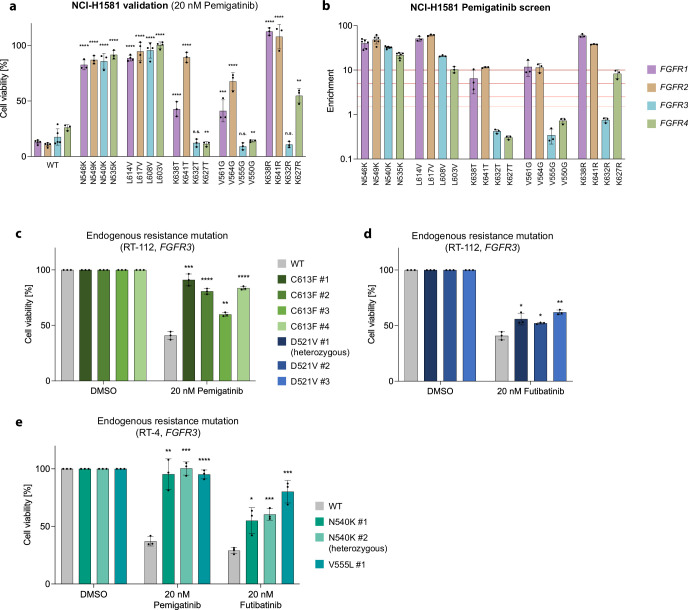
Validation of resistance across all *FGFR*s and endogenous validation of selected resistance mutations. **a-b**, Results of the pemigatinib resistance validation **(a)** and the screen **(b)** for five different amino acid mutations with similar or differential resistance across the four *FGFR*s. Mean viability of NCI-H1581 cells normalized to DMSO is presented ± standard deviation of n = 3 biological replicates (only for WT n = 5 and L614V, L617V, L608V and L603V n = 4) **(a)**. P values are calculated for each mutation compared to WT using a two-tailed Student’s t-test. Results of the pemigatinib resistance screen **(b)** with n = 3 and 95% CI. The four red colored lines represent the thresholds for effect strength classification. Mutations with n > 3 replicates result from multiple nucleotide mutations that lead to the same amino acid change. **c-e**, Validation of resistance for mutations endogenously introduced by precision genome editing. Mean viability of RT-112 and RT-4 cells normalized to DMSO is presented ± standard deviation of n = 3 biological replicates. P values are calculated for each mutation compared to WT using a two-tailed Student’s t-test. FGFR3 C613F **(c)** is a pemigatinib resistance mutation and FGFR3 D521V **(d)** is a futibatinib resistance mutation, both newly identified in this study and introduced into RT-112 cells (urinary bladder, FGFR3-TACC3). FGFR3 N540K and V555L **(e)** are both known double resistance mutations and were introduced and validated in RT-4 cells (urinary bladder, FGFR3-TACC3). Source data

The resistance screen results were validated for individually cloned mutations in two cell lines, compared to the CKB database, mapped onto the *FGFR1* structure, analyzed for the phosphorylation patterns of FGFR and FGF receptor substrate 2 (FRS2) and verified in endogenously mutated cells (Fig. 3c–f, Extended Data Figs. 5d–j and 6a–e and Supplementary Note).

In summary, our screen determined hundreds of point mutations conferring either selective resistance to pemigatinib or futibatinib or dual resistance to both inhibitors. More mutations introduced a resistance to pemigatinib and were conserved in at least two FGFRs with a highly significant correlation between all four FGFRs (Extended Data Fig. 2c,d).

### Prediction scores are insufficient to classify *FGFR* mutations

**Fig. 4:**
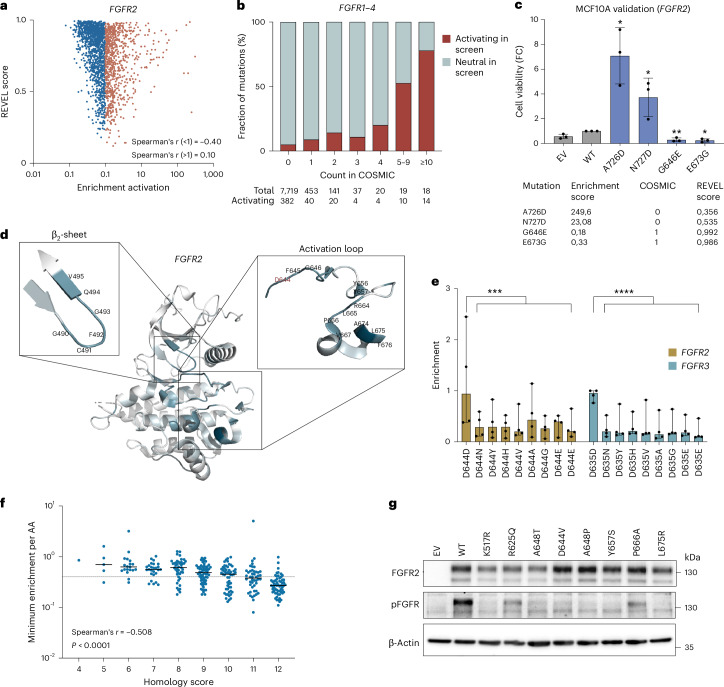
Comparison to in silico prediction scores and detection of LoF mutations. **a**, Correlation between REVEL score and median enrichments detected in the activation screen. For the REVEL score, there is weak positive correlation only with the positive (>1, orange) and a negative correlation with the negative (<1, blue) enrichment values, respectively. The respective *r* values are given in the figure and reflect Spearman’s correlation coefficient. **b**, Comparison of the mutation count in COSMIC and activation properties in the screen. **c**, Comparative validation of activating mutations absent in COSMIC and with low REVEL scores versus nonactivating mutations present in COSMIC with high REVEL scores in MCF10A cells. WT and EV constructs served as controls (gray). The data are presented as the mean of the fold-change in cell viability compared to the EV ± s.d. of *n* = 3 biological replicates. *P* values are calculated for each mutation compared to WT using two-tailed Student’s *t*-tests. **d**, Structure of the *FGFR2* kinase domain (PDB, accession no. 2PVF) with amino acids affected by LoF mutations marked in blue. The color intensity indicates the strength of the inactivation phenotype. The amino acids in the β_2_-sheet and the activation loop are zoomed in to depict the amino acids containing inactivating or strongly inactivating mutations. Asp644, which is part of the DFG motif, is marked in red. **e**, Enrichment of point mutations affecting the *FGFR2* Asp644 and *FGFR3* Asp635 residues in the activation screen. Amino acid changes listed twice originated from different nucleotide mutations. The median enrichment with 95% confidence interval of *n* = 4 independent biological replicates is depicted for each mutation. *P* values are calculated for all missense mutations compared to the synonymous mutation using two-tailed Student’s *t*-tests. **f**, Correlation between LoF and conservation of the respective amino acid in 12 RTKs. The dotted line represents the threshold value of 0.4 and the *r* value reflects the two-tailed Spearman’s correlation coefficient. The data are presented as the median of *n* = 4 biological replicates. **g**, Stable overexpression of eight different *FGFR2* mutations in MCF10A cells leading to decreased FGFR phosphorylation compared to WT overexpression in western blot analysis. Lys517Arg, Arg625Gln and Ala648Thr are well-known LoF mutations and Asp644Val, Ala648Pro, Tyr657Ser, Pro666Ala and Leu675Arg are newly identified in the activation screen. A representative blot for *n* = 3 biological replicates with similar results is shown. *P < 0.05, **P < 0.01, ***P < 0.001, ****P < 0.0001. Source data

**Extended Data Fig. 7:**
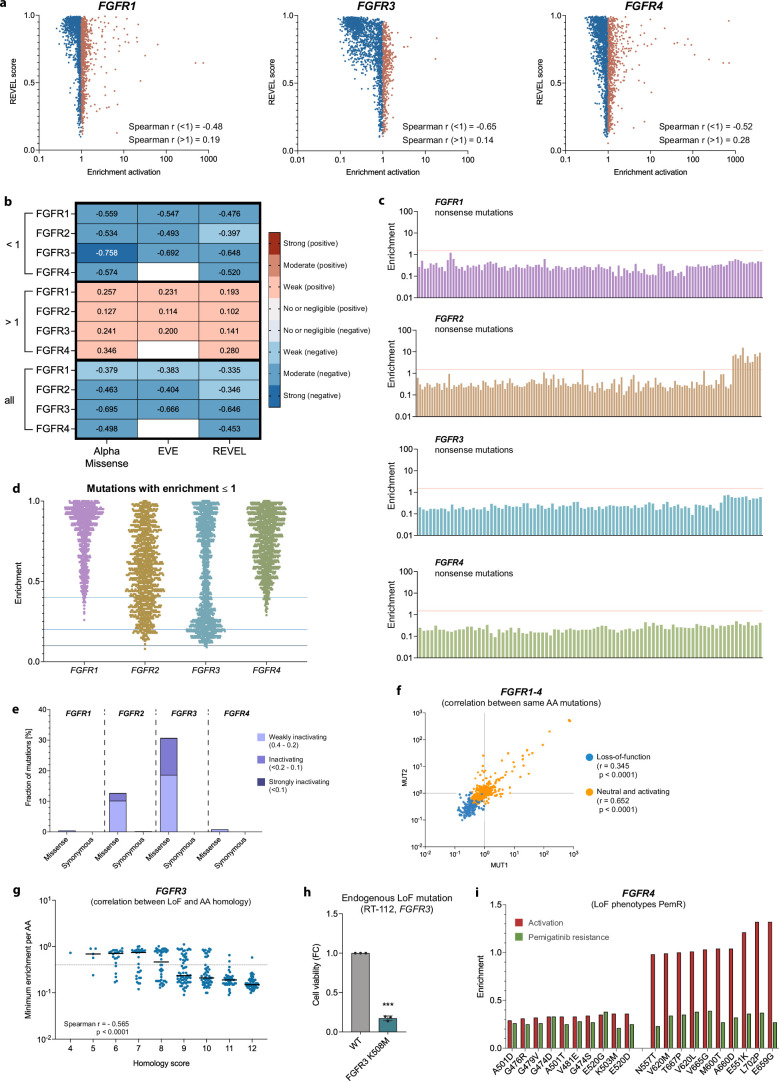
Comparison of functional predictions, nonsense mutations and loss-of-function mutations identified in the activation screen. **a**, Correlation between REVEL scores and median enrichments detected in the activation screens for *FGFR1*, *FGFR3* and *FGFR4*. There is no strong correlation between positive (enrichment >1, orange) and a strong correlation between negative (enrichment <1, blue) median enrichments and the REVEL score. The respective r value is given in the figure and reflects the Spearman correlation coefficient. **b**, Correlation between three different prediction scores (REVEL, EVE and AlphaMissense) and median enrichments detected in the activation screens. All scores showed moderate to very strong correlations for mutations depleted in the screen, but no or weak correlations for mutations enriched (GoF) leading to an overall negative correlation of enrichment versus prediction score. **c**, Enrichment of nonsense mutations introducing a stop codon in the activation screen for each *FGFR* reveals activation for C-terminal nonsense mutations in FGFR2 (n = 4). **d**, Enrichment values for depleted mutations identified in the activation screen. The three blue colored lines represent the thresholds for effect strength classification (≤0.4 = weakly inactivating, <0.2 = inactivating and <0.1 = strongly inactivating). **e**, Comparison of inactivating loss-of-function (LoF) missense and synonymous mutations (enrichment ≤0.4) and their categorization according to the effect strength for *FGFR1-4*. **f**, Correlation of the median activation (n = 4) between different nucleotide mutations giving rise to the same amino acid change being either depleted (blue) or neutral or enriched (yellow). The r value reflects the Spearman correlation. **g**, Correlation between the minimum enrichment of FGFR3 LoF mutations in the activation screen (n = 4) and conservation of the respective amino acid in twelve different RTKs. The black lines indicate the medians, the dotted line represents the threshold value of 0.4 and the r value reflects the Spearman correlation coefficient. **h**, Validation of the loss-of-function mutation FGFR3 K508M endogenously introduced by precision genome editing into RT-112 cells. The mean viability of RT-112 wildtype and K508M mutant cells is presented ± standard deviation of n = 3 biological replicates. P values are calculated for each mutation compared to WT using a two-tailed Student’s t-test. **i**, Depletion of selected *FGFR4* mutations in the activation (red) and pemigatinib resistance (n = 3) shows overlapping as well as independent mutations. Source data

Next, we compared our experimental results to in silico predictions. The Rare Exome Variant Ensemble Learner (REVEL) score is frequently used by clinicians for interpretation of VUSs and combines multiple variant effect prediction tools for pathogenicity (FATHMM, VEST, PolyPhen, SIFT, PROVEAN, MutationAssessor, MutationTaster, LRT, GERP, SiPhy, phyloP and phastCons)^44^. The comparison clearly showed that the REVEL scores were insufficient to predict FGFR GoF missense mutations: for nonactivating mutations (enrichment <1), there was a mostly moderate negative correlation^32^ with the REVEL score, whereas there was only a weak positive correlation for all enriched (>1) mutations (Fig. 4a and Extended Data Fig. 7a). The same applied to the EVE^45^ and AlphaMissense^46^ scores with moderate or strong negative correlations for mutations depleted (enrichment <1) in the screen, but only weak positive correlations for enriched mutations, leading to an overall negative correlation of enrichment versus prediction scores in all cases for all FGFRs (Extended Data Fig. 7b). In general, the comparison highlighted the shortcomings of the three scores not to distinguish between GoF and LoF predictions when classifying pathogenicity.

Recurrent occurrences in cancer could be envisioned to identify pathogenic mutations. Thus, we compared the frequency of mutations in COSMIC to their activating properties. For the few highly recurrent mutations in COSMIC (≥5), the proportion of experimentally determined activating mutations was higher than 50%. Mutations with a lower frequency in cancer showed an only moderately increased fraction of activating mutations compared to mutations never listed in COSMIC (Fig. 4b). To compare experimental data in an exemplary fashion to predicted pathogenicity and tumor occurrence, we cloned two mutations with high REVEL scores and presence in COSMIC but low activation in the functional screen, in comparison to two mutations with low REVEL scores and not found in COSMIC but high activation. The screening results of all four mutations were successfully verified (Fig. 4c), highlighting the need for experimental approaches to assess the functional impact of activating *FGFR* mutations.

### Nonsense, LoF mutations and hydrophobicity

**Extended Data Fig. 8:**
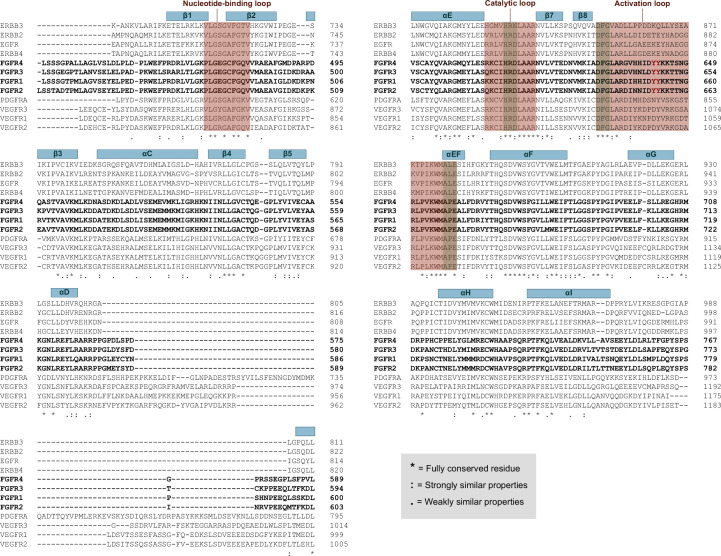
Alignment of the kinase domains of FGFR family members and other RTKs. The kinase domains of twelve related RTKs were aligned using CLUSTAL O. The indicated kinase domain substructures (α-helices and β-sheets) were identified based on McTigue et al.^20^. The nucleotide-binding loop, the catalytic loop and the activation loop are indicated by red boxes. The HRD (part of the catalytic loop), DFG and APE (parts of the activation loop) motifs are marked with green boxes. The two activation loop tyrosine residues are indicated with a bold red font. FGFRs are highlighted in bold. Source data

**Extended Data Fig. 9:**
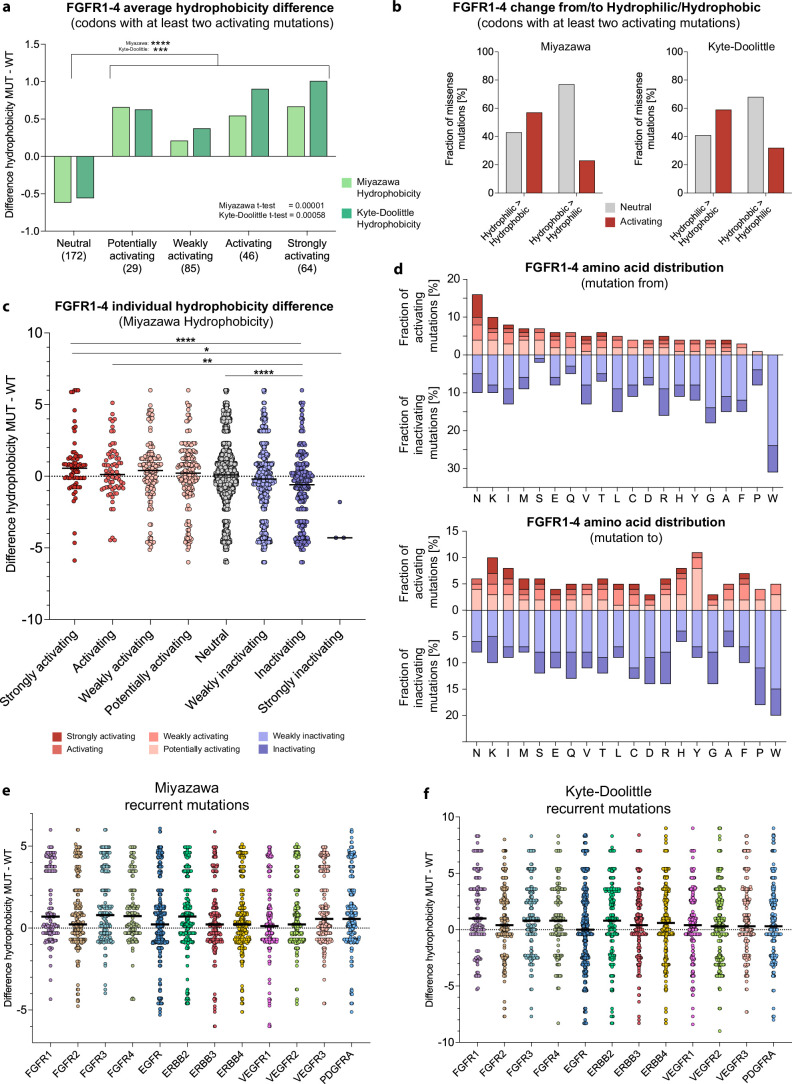
Amino acid properties and hydrophobicity of *FGFR1-4.* **a**, Comparison of the average hydrophobicity difference for missense mutations of codons with at least two activating mutations according to their activation strength. The hydrophobicity difference for each mutation was calculated by subtracting the hydrophobicity of the wildtype amino acid (WT) from the mutant amino acid (MUT) according to the Miyazawa or the Kyte-Doolittle scale (MUT-WT). The number of mutations in each category is indicated below the x-axis. *P* values are calculated for the hydrophobicity differences of neutral mutations versus all activating mutations using a two-tailed Student’s t-test. **b**, Comparison of the hydrophobicity switch of missense mutations of codons with at least two activating mutations. **c**, Difference in hydrophobicity (Miyazawa) for all missense mutations (*FGFR1-4*), grouped by their classification in the activation screen. The black line represents the median of each category. *P* values were calculated using a non-parametric Kruskal-Wallis test, comparing the median rank of each column to the median rank of every other column. Only significant differences are indicated. **d**, Fraction of activating or inactivating mutations for each of the 20 proteogenic amino acids from which the mutation originated (upper panel) and to which the amino acid was altered (lower panel). **e-f**, Hydrophobicity differences between WT and mutant amino acid of all COSMIC-annotated recurrent missense mutations for twelve different RTKs (median indicated by line). For the Miyazawa hydrophobicity scale **(e)**, the median increase in hydrophobicity is positive for all twelve RTKs, and for the Kyte-Doolittle hydrophobicity scale **(f)**, the median increase in hydrophobicity is positive for eleven out of twelve RTKs. Source data

Further analyses of the activation screens revealed activating carboxy-terminal nonsense mutations in *FGFR2* (ref. ^47^), identified, validated and characterized LoF mutations^48,49^ for activation and drug resistance and uncovered and orthogonally corroborated an association between GoF mutations increasing hydrophobicity and LoF mutations decreasing hydrophobicity in the resulting amino acid change (Fig. 4d–g, Extended Data Figs. 7c–i, 8 and 9a–f and Supplementary Note).

### Differential druggability of activating kinase domain mutations

**Fig. 5:**
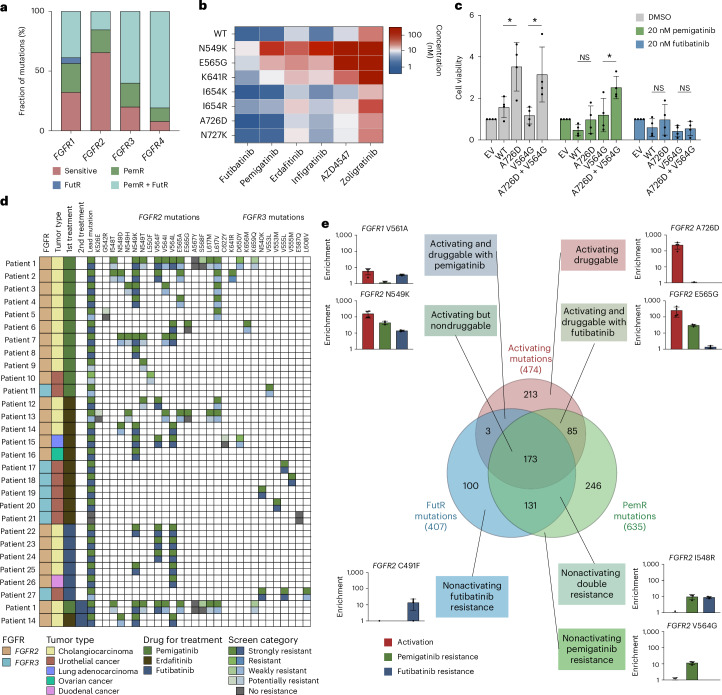
Combining activation and resistance data to determine the druggability of *FGFR* mutations. **a**, Distribution of GoF mutations identified in the activation screen classified according to their resistance phenotype determined in both inhibitor screens. **b**, Heatmap of IC_50_ values of NIH-3T3 cells expressing FGFR2 WT or 7 mutations treated with 6 different FGFRis ranging from 5,000 nM to 0.25 nM (*n* = 3). DMSO was used as a control for normalization. IC_50_ values were estimated using a four-parameter log(logistic) model. **c**, Validation of separate activation and resistance mutations in MCF10A. EV and WT constructs served as controls. The mean of the fold-change in cell viability compared to the EV ± s.d. is depicted (*n* = 4). *P* values are calculated as indicated in the graph using two-tailed Student’s *t*-tests. **d**, Resistance mutations were identified for 27 patients treated with 3 different FGFRis in clinical trials. Two patients were treated sequentially with two different inhibitors; thus, 29 genotypes were evaluated. FGFR sequencing upon acquired resistance revealed newly occurring point mutations. Comparison of the mutations detected in patients with acquired resistance to the enrichment of these mutations in the PemR and FutR screens revealed the identification of the resistance mutation in 28 out of 29 cases. **e**, Overview and classification of every enriched activating and resistance-conferring mutation identified in the saturation screening for the *FGFR1*–*4* kinase domains. The integration of the three datasets revealed seven distinct groups, according to their susceptibility to therapy or their resistance phenotype. *P < 0.05. Source data

**Extended Data Fig. 10:**
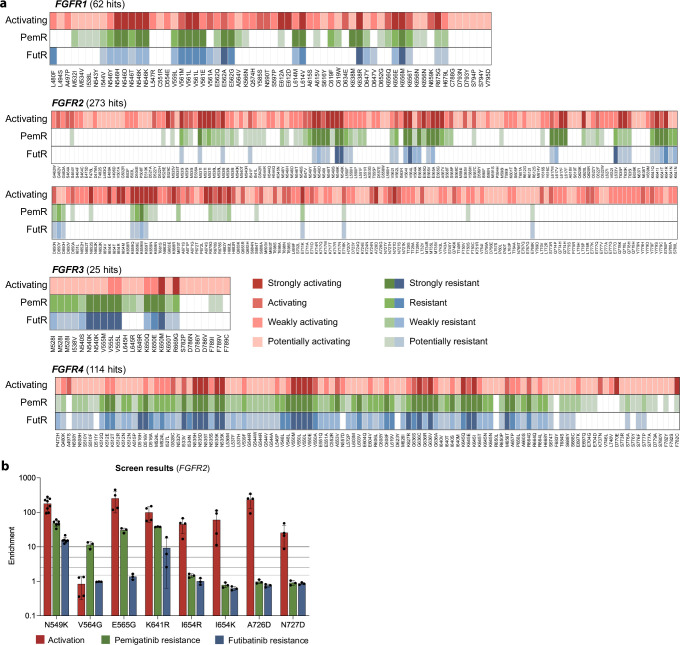
Druggability of activating FGFR mutations. **a**, Activating mutations in *FGFR1, FGFR2, FGFR3* and *FGFR4* and their classification for median activation (n = 4), pemigatinib and futibatinib resistance (n = 3) according to the respective screens. Amino acid mutations listed more than once arise from different nucleotide mutations resulting in the same amino acid change. **b**, Individual results of eight mutations regarding their activation (n = 4), pemigatinib and futibatinib resistance (n = 3) according to the respective screens. The data are presented as the median with 95% CI. Mutations with n > 4 replicates result from different nucleotide mutations that lead to the same amino acid change. The four grey lines represent the thresholds for effect strength classification. Source data

Combining the screen results for activation and resistance, we classified *FGFR1*–*4* kinase domain missense mutations according to their druggability (Extended Data Fig. 10a). Comparison of the activating mutations revealed clinically meaningful differences: for *FGFR1* and *FGFR2*, the majority of mutations were treatable with one or both FGFRis, whereas, for *FGFR3* and *FGFR4*, most mutations mediated resistance to both tested inhibitors (Fig. 5a).

To broaden the spectrum of FGFRis, we determined the IC_50_ values of six inhibitors for seven *FGFR2* mutations in NIH-3T3 cells (Fig. 5b and Supplementary Table 5). The resistance patterns for three known activating mutations with dual or differential resistance (Asn549Lys, Glu565Gly and Lys641Arg) and four newly identified mutations sensitive to both inhibitors (Ile654Lys, Ile654Arg, Ala726Asp and Asn727Asp) from the screens in NCI-H1581 were recapitulated in NIH-3T3 cells (Fig. 5b and Extended Data Fig. 10b). This experiment also supported the choice of pemigatinib and futibatinib for the screens because they showed the least resistance across these mutations.

### Resistance mutations in clinical trials match screening results

As secondary resistance mutations frequently emerge in patients with an *FGFR* translocation, amplification or activating mutation, we tested the combination of an activating sensitive mutation (*FGFR2* Ala726Asp) and a nonactivating pemigatinib-resistant mutation (*FGFR2* Val564Gly). As expected, the activating mutation conferred significantly increased cell proliferation to MCF10A cells, whereas only the combination was activating and resistant to pemigatinib but inhibited by futibatinib (Fig. 5c).

Finally, we compared the functional screening results to the clinical response of tumors after FGFR inhibition. We gathered clinical response data of 27 patients at the time of acquired resistance after treatment with one or multiple FGFRis: pemigatinib, futibatinib or erdafitinib (another noncovalent reversible FGFRi like pemigatinib)^27,50^. Two patients were consecutively treated with different inhibitors, making 29 datapoints with *FGFR* mutations and selected genetic co-alterations occurring at the time of acquired resistance available. Overall, 28 single nucleotide mutations were identified, of which 27 mediated resistance to pemigatinib (96%) and 22 to futibatinib (79%), whereas only one mutation (4%) did not mediate resistance to either drug in our screens. For the lead mutations, the mutation in a patient with the strongest drug resistance, we found 14 different ones (8× *FGFR2*, 6× *FGFR3*) with 13 of them (93%) showing resistance at any strength to pemigatinib and futibatinib (Fig. 5d). For acquired resistance to pemigatinib and futibatinib, all 19 cases (100%) harbored a resistance mutation (‘strongly resistant’ or ‘resistant’) according to the screens. For erdafitinib, which was not tested experimentally, the screening data uncovered a resistance mutation to the other reversible inhibitor pemigatinib for nine out of ten patients (90%). The one patient with only a non-resistance-mediating mutation possessed co-alterations in PIK3CA and TSC1. Several studies have linked the phosphoinositide 3-kinase (PI3K) pathway to FGFRi resistance but final evidence from a prospective clinical trial is still lacking. In urothelial cancer, *FGFR3* and PI3K showed strong synthetic lethality in a screen^51^. Another study identified the PI3K–mammalian target of rapamycin pathway (mTOR) as a main mechanism of off-target resistance^27^. Also, in *FGFR2*-driven cholangiocarcinoma, mutations in PIK3CA and PTEN frequently occurred upon acquired resistance to FGFRis^28^. However, such mutations have also already been found in responding tumors, so that the direct link to resistance remains to be proven in a clinical trial. In summary, in 28 out of 29 cases of acquired resistance to FGFRis (97%), the functional data would have correctly indicated the resistance based on the *FGFR* mutations (Fig. 5d and Supplementary Table 6).

For nine patients with acquired resistance to a reversible FGFRi, the functional screens would have indicated only potential (two) or weak resistance (seven) to the irreversible inhibitor futibatinib. In turn, for the two patients treated with futibatinib after a reversible inhibitor, the screening data would have correctly indicated that the tumor would also be resistant to futibatinib—and, indeed, both patients showed progressive disease under futibatinib.

To compare our dataset to a broader panel of resistance mutations, we performed a comprehensive literature search in PubMed. In total, we identified 21 studies^14,25,27,42,43,50,52–66^ that listed 56 different missense mutations caused by SNVs across all 4 FGFRs found in 138 patients upon acquired FGFRi resistance. Out of these 56 mutations found in patients, 51 mediated resistance to pemigatinib and/or futibatinib (91%) according to our functional screens, with 28 of them (50%) even strongly inducing resistance (>10-fold median enrichment) (Supplementary Table 7). All four *FGFR2* mutations not found in the resistance screens were identified in only one single patient each and each of these patients had between one and ten other mutations in *FGFR2* mediating strong resistance—thus, these four *FGFR2* mutations can be convincingly classified as bystander mutations. Only one out of 56 mutations remained in one single patient, which was neither linked to other *FGFR* mutations nor resistant in our functional screens. This was the same patient with the *FGFR3* Glu587Gln mutation as in our previous analysis (Fig. 5d).

In summary, the comparison of the functional screening data to clinical trial results indicated a very strong overlap between the classification based on screening and clinical outcome, further highlighting the validity of the functional categorization of actionable *FGFR* point mutations, thereby highlighting the clinical impact of these comprehensive analyses (Fig. 5e).

## Discussion

In-depth genomic characterization of tumor samples is an established approach to identify targetable genetic drivers in cancer. However, VUSs and the lack of functional data pose a major challenge in linking the genotype to the most promising targeted therapy. Specific therapeutic recommendations can be made only for the very limited number of well-characterized hotspot aberrations in the few well-studied cancer genes^67^. For the *FGFR* family, multiple inhibitors have been approved for cancer treatment by the FDA, but mostly for tumors with *FGFR* fusions or rearrangements^22–24,68^. In contrast, point mutations in *FGFR1*, *FGFR2*, *FGFR3* and *FGFR4* are especially difficult to interpret, because they barely cluster in hotspot regions, but are distributed across the entire gene sequence. In addition, the few studied activating kinase domain mutations often confer FGFRi resistance. Thus, patients with kinase domain mutations have often responded poorly in clinical studies^31,63,69,70^. Only point mutations in the extracellular domain of *FGFR3* and nonsense mutations leading to loss of exon 18 in *FGFR2* are currently considered for FGFRi treatment^47^. Although multiple studies have examined individual *FGFR* mutations, these studies did not cover the vast majority of mutations found in cancer^28,38,42,53^. However, it should be mentioned that previous studies have analyzed individual FGFR-activating mutations before beyond the CKB and OncoKB panel, which was used as the basis to categorize ‘known’ and ‘new’ mutations in this study.

We successfully established a saturation mutational scanning platform to determine the activating and resistance-mediating properties of all possible 11,520 *FGFR* kinase domain SNVs resulting in a comprehensive catalog classifying the impact of each mutation on activation and drug resistance. *FGFR* kinase point mutations should not be generally excluded from FGFRi treatment, but should be actively considered if they are activating and do not mediate drug resistance. We identified 213 activating mutations treatable with both drugs and 88 additional activating mutations druggable with futibatinib or pemigatinib, of which 205 and 72, respectively, have so far not been annotated as (predicted) GoF in the CKB or OncoKB. Resistance-conferring mutations could also serve as contraindicators against FGFRi treatment on the background of activating translocations or amplifications.

Multiple lines of evidence unanimously validate the robustness and reliability of the screening results: (1) independent biological replicates correlated significantly; (2) different nucleotide mutations giving rise to the same amino acid change correlated significantly; (3) functional mutations for GoF or LoF were strongly enriched in missense over synonymous mutations; (4) previously identified GoF mutations from the CKB or OncoKB or from moderate-throughput studies^38,71,72^ and the activating nonsense mutations at the *FGFR2* C-terminus^47^ were all recapitulated in the screen; (5) CKB resistance mutations were identified in the screen, with only those mutations missing that were specific for an inhibitor not included in this work or that occurred in patients with multiple resistance mutations^53,54^; (6) in total, 50 individually cloned mutations verified the screen results; (7) identified activating mutations occurred in a broad spectrum of tumor subtypes; (8) results for activation and resistance were each confirmed in a second independent cell line model; and (9) the functional data comprehensively identified acquired resistance mutations in a clinical study with a concordance rate of 97%.

Although patient-derived cell lines lack the microenvironment and immune status possibly present in organoids, animal models or clinical studies, they enable high-throughput screening of thousands of mutations in a massively parallel pooled approach, which is not generally feasible in the aforementioned systems. In addition, the cell-autonomous impact of the *FGFR* mutation on cell proliferation and drug resistance does not depend on the microenvironment and the immune system. To the contrary, the homogeneity of the cell line model may allow a more robust and reproducible quantification of a variant’s effect.

As different mutations of the same codon had similar or fundamentally different properties, which was also true for the corresponding mutations in the four different FGFRs, the screen results also emphasize the need for the saturation mutagenesis approach because every mutation needs to be tested individually.

Although the FGFR kinase domains are very similar at the sequence level, we found large differences in the number of activating and targetable clinically relevant kinase mutations. Most highly activating and targetable mutations were detected in *FGFR2*, correlating with the number of GoF mutations listed in the CKB. Most *FGFR4* mutations were not targetable with the inhibitors tested in this study, which is consistent with studies showing that pemigatinib efficiently inhibits *FGFR1*–*3* but has lower potency against *FGFR4*^73^. It can thus be expected that activation of *FGFR4* may already suffice to overcome pemigatinib sensitivity, which is confirmed in our data because >92% of the activating *FGFR4* mutations also show resistance to pemigatinib, compared to only 43% of the activating mutations in the other FGFRs. Futibatinib, currently promoted as a pan-FGFRi, also showed limited efficacy against *FGFR4* kinase domain mutations. In the future, a specific *FGFR4* inhibitor like fisogatinib could be tested for *FGFR4* mutations^74^.

*FGFR3* had a lower number of activating mutations compared to other FGFRs. Importantly, *FGFR3* amplifications are rarely observed and have not been associated with any particular type of cancer in contrast to other *FGFR*s^26^. Nevertheless, our screen successfully identified the well-characterized *FGFR3* GoF mutations in the kinase domain^37^.

In contrast to the more heterogeneous distribution of activating mutations between the FGFRs, the number and overlap of resistance mutations was quite similar for *FGFR1*–*3*, whereas *FGFR4* resistance mutations were more individual and frequent. Individual *FGFR* resistance mutations mostly affected amino acids that were not conserved across the four FGFRs.

Next to the GoF mutations, the additionally identified LoF mutations are of particular interest for disease syndromes that lead to craniosynostosis conditions, such as *FGFR1*-associated Pfeiffer’s, Hartsfield’s and Kallmann’s syndromes^75,76^. Importantly, all previously reported dominant-negative *FGFR1* mutations Gly487Asp, Met535Lys, Asp623Glu, Asp641Asn and Pro772S were underrepresented compared to synonymous mutation controls and were also depleted in *FGFR2*, *FGFR3* and *FGFR4*. Moreover, *FGFR2* LoF mutations have been linked to drug resistance in melanoma^77^. Notably, 78% of the *FGFR2* LoF mutations recurrently found in COSMIC also occurred in malignant melanoma, further pointing toward a subtype-specific relevance of *FGFR* LoF mutations. Nevertheless, this LoF phenotype has so far mostly been tested in the context of overexpression.

Effective mutations clustered in structural regions and associated with changes in hydrophobicity. It is interesting that hydrophobic changes were significantly more likely to be associated with GoF mutations and hydrophilic changes with LoF mutations. The FGFR kinase domains possess the conserved bilobed structure of all eukaryotic protein kinases, based on a hydrophobic core architecture. Two hydrophobic spines serve as noncovalent links between the two lobes of the domain. Their dynamic orientation upon phosphorylation of the activation loop and ATP binding is essential for the kinase activity^78^. Thus, our results that mutations altering hydrophobic to hydrophilic residues are enriched for LoF effects are concordant with the expected inhibitory consequences of disrupting the hydrophobic core architecture. Selected mutations in these hydrophobic regions can nevertheless cause constitutive activation as shown for oncogenic *BRAF*^79^, and are also recapitulated in our screen.

Purely computational approaches represent an alternative to experimental high-throughput studies for predicting the functional effect of variants observed in patients. The lack of an overall positive correlation of the functional screening results with the REVEL, EVE or AlphaMissense prediction scores showed that this is not sufficient to predict the pathogenicity of an individual *FGFR* GoF mutation. Moreover, predictions for drug response are often unavailable.

Machine learning algorithms have been used to perform in silico saturation mutagenesis. Such approaches have shown promising results for selected cancer genes like *TP53*, *CTNNB1* and *EGFR*, but also require a comprehensive set of data^80^, which are not available for every cancer gene. For example, the database BoostDM only covers *FGFR3* based on mutational data from bladder cancer and provides no information at all for *FGFR1*, *FGFR2* or *FGFR4*.

For future research, we envision the expansion to further FGFRis, especially compounds still in clinical development like the *FGFR2*-specific inhibitor lirafugratinib or RLY-4008^81,82^ or the *FGFR4*-specific fisogatinib^74^. The established saturation mutational scanning platform allows the rapid testing of newly emerging FGFRis.

The focus could be expanded to individual synonymous mutations that could affect the activity or drug response^33,83,84^, although the vast majority of synonymous mutations did not show any effect in the functional screens and could thus be used for normalization.

Genetic co-alterations in genes like *TP53*, *PTEN* or *EGFR* or the MAPK/ERK or PI3K pathway^27,42,63,85–87^ have been linked to FGFRi resistance. Thus, the analysis of these co-alterations would be important for the prediction of the effective use of FGFRis or to rationally design combination therapies^88^.

In conclusion, the comprehensive dataset of actionable mutations generated here is an important step toward personalized therapy using FGFRis in tumors harboring *FGFR* missense mutations. For mutations with no functional, genetic or clinical data available, which is the case for most *FGFR* point mutations found in an individual patient, our comprehensive catalog provides druggability information comparable to a strong PS3/BS3 evidence level. A clinically highly relevant advantage of this prospective saturation approach is the immediate availability of the information when a point mutation is identified in a patient with cancer—eliminating the need for time-consuming analysis after finding the mutation, which is crucial given the limited time that patients with cancer have for therapy decisions.

## Methods

All research presented in this study complies with all relevant legal and ethical regulations of Germany and France.

### *FGFR* saturation mutagenesis, library design and cloning

The coding sequences (CDSs) of *FGFR1*–*4* were extracted from the Consensus CDS Database (National Center for Biotechnology Information (NCBI)) using the RefSeq Match transcript as indicated at Ensembl^89^ and the kinase domain of each *FGFR* was determined using UniProt^90^ (Supplementary Table 8). Due to size limitations for pooled oligonucleotide libraries (oPools with maximum length of 300 nt; TWIST Bioscience), we divided each kinase domain into 4 pools, each covering 80 amino acids, resulting in 16 independent libraries for *FGFR1*–*4*. The oPools with a total of 720 sequences per library, covering every possible substitution mutation at the nucleotide level in the respective sequence, were designed using customized code in R. To integrate the oPools into destination plasmids using Gibson Assembly Master mix (New England Biolabs), each sequence was flanked with wild-type (WT) overhangs of 30 nt. Lentivirus-compatible destination vectors consisted of a pHAGE backbone harboring an *EF1α* promoter driving expression of *FGFR*, followed by a T2A signal and a puromycin resistance cassette. *FGFR* CDSs were ordered as clonal genes, including respective type-II-S restriction enzyme sites for each oPool integration (TWIST Bioscience).

Delivered oPools were amplified using NEBNext Ultra II Q5 Master Mix (New England Biolabs) according to the manufacturer’s protocol. Backbones were digested with the respective restriction enzymes (PaqCI, Esp3I or BaeI). Amplicons and backbones were gel purified using the GeneJET gel extraction kit (Thermo Fisher Scientific) and further purified and concentrated using AMPure XP magnetic beads (Beckman Coulter). Purified oPools were integrated via Gibson Assembly Master Mix and transformed into ElectroMAX DH10B bacteria (Thermo Fisher Scientific, cat. no. 18290015). After calculating transformation efficiencies, bacteria were plated to achieve a coverage of at least 200× (approximately 180,000 colonies per library). Bacterial colonies were scratched from plates and plasmid DNA was extracted using the PureLink HiPure Plasmid-Filter Maxiprep Kit (Thermo Fisher Scientific). The resulting plasmid library was subsequently used to produce lentiviruses according to the protocol described in Supplementary Information. The lentivirus supernatants were tested for transduction efficiency in NCI-H1581 and MCF10A cells to determine the amount of virus needed for a multiplicity of infection (MOI) of 0.3.

NGS of the obtained saturation libraries verified their very high homogeneity and the presence and near-equimolar representation of all mutations.

### High-throughput activation screen

For each replicate, MCF10A cells were plated in four 15-cm dishes with a density of 1.3 × 10^6^ cells per plate 24 h before transduction (day 0) and lentivirus libraries were transduced with an MOI ≤ 0.3 on day 1. Exchange of transduction medium was performed 24 h after transduction (day 2) and, after another 24 h, the cells were treated with puromycin (MedChemExpress, 3.5 µg ml^−1^, day 3) for 48 h. On day 5, cells were washed 3× with phosphate-buffered saline, trypsinized and resuspended in assay medium lacking growth factors and with serum reduced to 2%. After centrifugation (200*g* for 5 min), cells were transferred into a 225-cm^2^ flask and incubated for 7 d. Assay medium was refreshed on day 8. On day 12, cells were resuspended, centrifuged and, dependent on density, transferred to a 15-cm dish or a 225-cm^2^ flask. Afterward, cells were again incubated for 5–8 d, dependent on confluency with a medium refreshment on day 15. Cells were harvested between day 17 and day 20, dependent on confluency and gDNA was isolated using the DNeasy Blood & Tissue Kit (QIAGEN).

### High-throughput resistance screen

For each replicate, NCI-H1581 cells were plated in one 15-cm dish with a density of 11.25 × 10^6^ cells per plate 24 h before transduction (day 0) and lentivirus libraries were transduced with an MOI ≤ 0.3 on day 1. Exchange of transduction medium was performed 24 h after transduction (day 2) and, after another 24 h, the cells were treated with puromycin (3 µg ml^−1^, day 3), which was refreshed after 24 h (day 4). On day 5, cells were trypsinized, counted and transferred into a 225-cm^2^ flask and the respective FGFRi was added on day 6 (with a concentration of 20 nM for pemigatinib and 10 nM for futibatinib). Cells were incubated with the FGFRi for 72 h. For the pemigatinib screen, cells were resuspended on day 9 and again treated with the FGFRi whereas, for the futibatinib screen, only medium and FGFRi were refreshed on day 9. After incubation for another 96 h, cells were harvested, centrifuged and gDNA was isolated using the DNeasy Blood & Tissue Kit.

### Next-generation sequencing

The NGS library preparation entailed two rounds of PCR amplification. In the first round, plasmid DNA or gDNA was amplified using target-specific primers (Supplementary Table 9) using NEBNext Ultra II Q5 Master Mix according to the manufacturer’s protocol. For gDNA, a total of 3,000 ng and, for plasmid DNA, a total of 30 ng was used for amplification. Amplicons were purified using 0.9× *V* of AMPure XP magnetic beads (Beckman Coulter; *V* is the volume of the DNA sample) and 30–60 ng of the purified product was used as a template in a second round of PCR amplification. The primers of the second PCR consist of Illumina adapter-specific, index-specific and target-specific sequences and the forward primers also containing a degenerate sequence of six to nine random bases (Supplementary Table 9). NEBNext Ultra II Q5 Master Mix was again used for amplification according to the manufacturer’s protocol. The PCR product was gel purified using the GeneJET gel extraction kit (Thermo Fisher Scientific) and further purified and concentrated using 0.7× *V* of AMPure XP magnetic beads (Beckman Coulter). The resulting samples were pooled in equimolar portions and diluted to 4 nM. Libraries were spiked with 35% PhiX control (Illumina, cat. no. FC-110-3001), denatured using 0.2 M NaOH and diluted to 10 pM according to the manufacturer’s protocol. The MiSeq Reagent Kit v.3 (600-cycle, Illumina, cat. no. MS-102-3003) was used on the Illumina MiSeq platform and paired-end sequencing with a read length of 210 nt was performed. All samples were sequenced with a coverage corresponding to at least 300 reads per point mutation in the reference library.

### NGS analysis

Fastq data files were analyzed using Galaxy^91^. The quality of each sample of one NGS run was reviewed using FastQC. Forward reads were trimmed using Trimmomatic^92^. The HEADCROP Trimmomatic operation was selected with ten bases that were removed from the start of the read. The trimmed forward read and the reverse read were merged using FLASH with a minimum overlap of 10, a maximum overlap of 200 and a maximum mismatch density of 0.25. In case the same index was used for different targets, Cutadapt^93^ was performed to split the samples based on customized target adapter sequences. Matches were left unchanged and a separate file was created for each adapter. Mapping was performed using ‘Map with BWA-MEM^94^ for medium and long reads ≥100 bp’ using a 260-nt-long reference sequence corresponding to the *FGFR* WT sequence in the respective region. The decision for the best algorithm was set to automatic and default settings were used, with only scoring options 0 used as a penalty for a mismatch. Samtools^95^ calmd was used to change identical bases to ‘=’ and the resulting BAM files were formatted to SAM files using Samtools view. Final SAM files were downloaded from Galaxy and used for analysis in R. Analysis in R was based on a customized code to identify reads harboring only a single-point mutation and the frequency of each read was determined. The results of different replicates and screens of the same library were merged with the initial library design file and exported as Excel files. The results were further analyzed in Excel by first normalizing the individual read counts to the total number of read counts in the library. Then, the results were normalized to the read coverage of the plasmid library (median of three replicates). To determine whether reads were enriched compared to the background, the normalized frequency of each point mutation was divided by the median calculated from all synonymous mutations (which served as a control), resulting in the final enrichment score.

For further analysis of statistical significance of enriched mutations, enrichment scores were log(transformed) and *P* values were calculated using two-tailed Student’s *t*-tests, with all replicates of synonymous mutations serving as the control group. Variance of the different groups was determined using an *F-*test and relevant Student’s *t*-tests were applied. *P* values were further false discovery rate corrected using the two-stage step-up method of ref. ^96^ and discoveries were determined with *Q* = 1% as the maximum acceptable false positive rate (Supplementary Tables 10 and 11). To designate a mutation as a hit, we defined a median of ≥1.5 in the final enrichment score and enrichment in at least 2 of 3 replicates for resistance and 3 of 4 replicates for activation as sufficient, due to the high s.d. obtained for strongly activating mutations. The statistical significance is given for all missense mutations in Supplementary Table 10 and can be used for further assessment of mutation effects.

### Acquired resistance mutations in clinical trials for FGFRi response

In total, 27 patients were treated with an FGFRi (pemigatinib, futibatinib or erdafitinib) in clinical trials. Patient recruitment took place between 2015 and 2022. At progression, the molecular analyses were performed within two institutional studies: MATCH-R (NCT02517892) and STING (NCT04932525). All patients participating in these studies were fully informed and signed a written informed consent. The studies have been approved by ethics committees in France (French National Agency for Medicines and Health Products Safety) and were conducted in accordance with the Declaration of Helsinki.

*FGFR* mutations before treatment, as well as upon acquired resistance, were determined in the tumors and compared to the functional screening results. If multiple mutations occurred in the same tumor, the mutation conferring the maximum level of resistance was reported as lead mutation. Two patients were consecutively treated with two different FGFRis with newly appearing mutations upon acquired resistance, so that 29 datasets were available for analysis.

### Reporting summary

Further information on research design is available in the Nature Portfolio Reporting Summary linked to this article.

## Online content

Any methods, additional references, Nature Portfolio reporting summaries, source data, extended data, supplementary information, acknowledgements, peer review information; details of author contributions and competing interests; and statements of data and code availability are available at 10.1038/s41588-025-02431-8.

## Supplementary information

Supplementary Information Supplementary Results and Methods.

Reporting Summary

Supplementary Tables Supplementary Tables 1–16.

Supplementary Table Precise *P* values.

## Source data

Source Data Figs. 1–5 Statistical source data.

Source Data Extended Data Figs. 1–10 Statistical source data.

Source Data Figs. 1, 3 and 4 Unprocessed western blots.

## Data Availability

The 208 NGS datasets generated during the current study have been deposited in the NCBI Sequence Read Archive repository with BioProject accession no. PRJNA1136966. The design and analysis pipelines have been deposited at GitHub and are accessible at https://github.com/Ctangermann/Saturation-mutagenesis-library-generator and https://github.com/Ctangermann/SNV-analyzer. The screening results have been deposited at MAVEdb and are accessible at https://www.mavedb.org/search?search=urn:mavedb:00001220-a. The authors declare that all other data supporting the findings are available within the paper and Supplementary Information. Source data are provided with this paper.

## Code availability

The R code used for detailed analysis of deep sequencing results is available via Zenodo at 10.5281/zenodo.17251670 (https://zenodo.org/records/17251670)^97^.
