# Supplementary material for: Saturation mutagenesis identifies activating and resistance-inducing FGFR kinase domain mutations
Source: Nat Genet. 2025 Dec 8;58(1):157–68. doi: 10.1038/s41588-025-02431-8 (PMC12807871; doi:10.1038/s41588-025-02431-8)
Supplement: Supplementary file 1 — Supplementary Results and Methods. [file 41588_2025_2431_MOESM1_ESM.pdf]

nature genetics

Supplementary information

https://doi.org/10.1038/s41588-025-02431-8

Saturation mutagenesis identifies activating and resistance-inducing FGFR kinase domain mutations

In the format provided by the authors and unedited

Supplementary Information - Table of Contents

SUPPLEMENTARY RESULTS

SUPPLEMENTARY METHODS

SUPPLEMENTARY METHODS REFERENCES

SUPPLEMENTARY TABLES 1 - 16
see separate xlsx file

Supplementary Results

Saturation mutational scanning libraries and screen controls

The pooled plasmid libraries of FGFR single nucleotide variants were analyzed via next generation sequencing (NGS) to ensure that all oligos were present, and their abundance in the library was used to normalize subsequent assays. All plasmid libraries covered 100% of the intended mutations, were highly homogeneous and contained mutations at largely equimolar ratios (**Extended Data Fig. 1**).
As quality controls for the screens, we confirmed the highly significant correlations between independent biological replicates (n=4 for activation, n=3 for resistance) (**Fig. 1c, Extended Data Fig. 2a, Supplementary Table 1**) and the also highly significant correlations between nucleotide mutations leading to the same amino acid change (**Fig. 1d, Extended Data Fig. 2b, Supplementary Table 2**). Also the pairwise comparison of the enrichments for activation or drug response between all four FGFRs revealed highly significant moderate to strong positive correlations[32] (**Extended Data Fig. 2c,d**).
The saturation libraries included 2667 synonymous mutations, *i.e.* mutations altering the nucleotide sequence of the mRNA but not the amino acid sequence of the protein due to the degeneracy of the genetic code. Although synonymous mutations can impact protein expression[33], this is rather rare and most of these mutations are expected to be neutral. The strong enrichment of effective missense mutations over synonymous mutations thus further validated our screen (**Fig. 1e, Extended Data Fig. 2e**).

Analysis and validation of FGFR activation screens

Comparison of the activation screen results with the CKB and OncoKB databases revealed that >90% of activating mutations in FGFR1, FGFR2 and FGFR4 identified in our screen had so far not been classified as GoF (**Fig. 2d**). For FGFR3, the overall number of activating mutations was

low with thus 44% already classified as GoF. Conversely, the screens correctly identified 90% of the known GoF mutations from CKB (**Fig. 2e, Extended Data Fig. 3b-d**). The four amino acid changes with discordant results between the screen and the CKB annotation were cloned and tested individually, but no mutation showed significant activation - thus corroborating the screening results (**Extended Data Fig. 3e**).

Since FGFR mutations occurred across tumor subtypes, we verified that results of the activation screen conducted in MCF10A cells, an epithelial cell line derived from the mammary gland, reflected pan-cancer mutations and not potentially restricted to breast cancer. We identified 36 activating and CKB-annotated GoF mutations and analyzed their occurrence in patients in COSMIC, where they were found in total 356 cases distributed across 24 different tumor subtypes (**Fig. 2f**). Thus, the screen identfied activating mutations not restricted to a subtype, but found across tumor subtypes. When analyzing all FGFR1-4 mutations listed in COSMIC, recurrent ($n \geq 3$) activating mutations were also found in a large number of different tumor subtypes (**Extended Data Fig. 3f**).

For validation, we cloned all mutations for the FGFR2 codons K659 and the gatekeeper V564, both of which had entries in CKB/OncoKB for some but not all mutations. In the screen, the six amino acid mutations for each codon showed different activation levels (**Extended Data Fig. 3g**), which were concordant with individual validation experiments (**Fig. 2g**). The differential effects of these mutations of the same codon emphasized the need to characterize every mutation individually by saturation mutagenesis. For further validation, we selected five known and six newly identified GoF mutations, which showed strong activation in the screen including mutations in the three molecular brake triad residues N549, E565 and K641[39] (**Extended Data Fig. 3h**). All eleven mutations led to significantly increased cell viability in comparison to the wildtype (**Fig. 2h**). Western blot analysis confirmed expression of all six tested mutant proteins and caused an increased phosphorylation of FGFR and its downstream target FRS2 (**Fig. 2i**). To ensure that the activation was not cell type-restricted, seven mutations were analyzed in mouse embryonic fibroblasts NIH-3T3 in a low serum proliferation assay. All mutations showed stronger proliferation than the wildtype control and significantly lower effective doses of serum (higher ΔED_{50}) needed for proliferation (**Fig. 2j**) confirming the screen results in MCF10A cells (**Supplementary Table 4**). NIH-3T3 colony formation was also significantly higher for the five tested mutations compared to *FGFR2* WT (**Fig. 2k, Extended Data Fig. 3i**).

For three new activating mutation positions close to the C-terminus of the kinase domain, we generated structural predictions using AlphaFold2 modelling. Mutations of FGFR2 N727, L732 and A726, that differed in their activation capacity in the screen, also had differential predicted impacts on the positioning of the tyrosine residues Y656 and Y657, which are known to be critical autophosphorylation sites within the activation loop, with Y656 phosphorylation preceding Y657[40,41]. For N727 and L732, there was a general trend for activating mutations more strongly altering the tyrosine positioning relative to the WT than for non-activating mutations (**Extended Data Fig. 4a-c**).

In conclusion, we identified hundreds of activating kinase domain mutations in *FGFR1-4*. The respective somatic mutations in cancer covered a broad spectrum of tumor subtypes.

Validations in two different cell lines indicated a high level of reproducibility, robustness and universal validity of the mutational scanning data.

Validation of the resistance mutations to pemigatinib or futibatinib

For validation of the resistance screen results, we cloned nine *FGFR2* mutations affecting the V564 and C491 codons, which showed dual as well as selective pemigatinib and futibatinib resistance in the screen (**Extended Data Fig. 5d**) successfully verified in the individual validation (**Fig. 3c**).

To ensure that resistance patterns were not restricted to the cell type or the tumor subtype, we used the FGFRi-sensitive urinary bladder cancer cell line RT-112 harboring an FGFR3-TACC3 fusion for an independent validation of selected resistance mutations (**Extended Data Fig. 5e**) correlating significantly with the validation results in NCI-H1581 corroborating the robustness of the results (**Fig. 3d**).

Comparing the few known resistance mutations annotated in the CKB database to our resistance screens showed that we identified 83% of the CKB-annotated resistance mutations (**Extended Data Fig. 5f**) - which was remarkable especially since the CKB resistance mutations were not specific to the tested inhibitors.

In contrast to the more FGFR-specific activation patterns, more than half of the resistance mutations occurred in two or more *FGFR*s for both drugs (referred to as conserved resistance) with the exception of *FGFR4* with more unique resistance mutations (**Extended Data Fig. 5g-j**). Twelve mutations conferred resistance in all four FGFRs for both pemigatinib and futibatinib, 16 conserved resistance mutations specifically for pemigatinib and nine for futibatinib, which were all mapped to the FGFR1 structure in complex with pemigatinib (**Fig. 3e**). While specific FutR mutations clustered at the beginning of the kinase domain, particularly in the nucleotide binding loop, specific PemR and double resistance mutations clustered in the central part of the kinase domain - several in close proximity to each other (**Fig. 3e**).

To verify the resistance at the molecular level, western blotting confirmed increased phosphorylation levels of FGFR2 and FRS2 after pemigatinib treatment by FGFR2 K659M, V564G and L633V, while K659M, C491S and L633V conferred increased phosphorylation upon futibatinib treatment (**Fig. 3f**).

For further validation, we cloned 20 mutations affecting five conserved codons in *FGFR1*, *FGFR2*, *FGFR3* and *FGFR4* for PemR with either universal or differential resistance between the FGFRs, which successfully verified the screen results for all 20 mutations (**Extended Data Fig. 6a-b**).

Lastly, we also verified resistance mutations at the endogenous level introduced by precision genome editing using CRISPR/Cas9. Since sufficient endogenous FGFR expression and a defined dependency on FGFR signaling as well as a minimum efficiency of genome editing were needed, we selected the urothelial bladder cancer cell lines RT-112 and RT-4 harboring aberrant FGFR3 activation. All four clones with edited FGFR3 C613F showed resistance to pemigatinib, all three clones with edited FGFR3 D521V showed resistance to futibatinib and all three clones with edited FGFR3 N540K or V555L showed resistance to both FGFRis

validating the screening results in homozygous as well as heterozygous settings (**Extended Data Fig. 6c-e**).

Activating C-terminal nonsense mutations in FGFR2

Besides missense and synonymous mutations, our screen also covered 446 nonsense mutations introducing a premature stop codon. We detected twelve activating *FGFR2* nonsense mutations at the end of the kinase domain resulting in a loss of exon 18. As truncating *FGFR2* mutations have been reported to result in FGFR2 activation[47], this additionally validated our screen results (**Extended Data Fig. 7c**).

Detection of loss-of-function mutations in the FGFR kinase domains

In the screens, which had been designed to identify GoF mutations by positive selection, mutations were also depleted showing negative selection (**Extended Data Fig. 7d-i**). LoF mutations could act in a dominant-negative manner interfering with the endogenous FGFR signaling of the cell by either dimerizing with the endogenous monomers or by binding to and thus competing for ligands and downstream interaction partners[48,49]. In addition, LoF mutations without dominant-negative effects could be outcompeted in the pooled libraries by the majority of neutral mutations with a wildtype-like effect and thus depleted.

Missense LoF mutations were classified into 634 weakly inactivating (<= 0.4, i.e. 2.5- to 5-fold depleted), 304 inactivating (<0.2, i.e. >5- to 10-fold depleted) and three strongly inactivating (<0.1, i.e. >10-fold depleted) mutations in at least three of the four replicates.

As one measure to determine the specificity of the LoF effects, we compared missense and synonymous mutations (mostly likely neutral): indeed, 941 out of 8407 missense mutations (11.19%) showed a LoF effect compared to only 1 out of 2667 synonymous mutations (0.04%). Thus, LoF were enriched 299-fold in missense over synonymous mutations indicating a specific and non-random observation (**Extended Data Fig. 7e**).

Pairs of missense mutations differing at the nucleotide level but giving rise to the same amino acid change indicated a highly significant correlation of LoF effects further corroborating the specificity of the observed LoF mutations (**Extended Data Fig. 7f**). Also, the predicted LoF mutations from CKB were 3.8-fold enriched among the mutations with LoF effects compared to all mutations.

When mapping the minimum enrichments per amino acid to the FGFR2 kinase domain structure, LoF mutations clustered specifically in highly conserved regions like the ß2-sheet and the activation loop (**Fig. 4d**). The activation loop harbors the DFG motif, involved in ATP and magnesium binding in the active site. Mutations altering the aspartic acid in this motif, FGFR2 D644 or FGFR3 D635, showed LoF effects, while the synonymous mutations at these positions behaved neutral (**Fig. 4e**).

To relate the functional data to conservation, we performed a protein sequence alignment of FGFR1-4, EGFR (ERBB1), ERBB2-4, PDGFRA and VEGFR1-3 (**Extended Data Fig. 8**) and defined a homology score for each amino acid indicating in how many of these twelve RTKs this amino

acid was identical. For FGFR2 and FGFR3 with most LoF mutations, we compared the minimum enrichment score for each amino acid to its homology score finding a highly significant negative correlation, *i.e.* a stronger conservation associated with a stronger LoF (**Fig. 4f, Extended Data Fig. 7g**). Furthermore, we verified the LoF phenotype at the molecular level for eight different FGFR2 LoF mutations and confirmed their inhibitory impact on FGFR phosphorylation by western blotting (**Fig. 4g**), including the Y657S mutation affecting an FGFR2 autophosphorylation site.

Lastly, we endogenously introduced the FGFR3 K508M mutation by precision genome editing homozygously into the FGFR3-driven RT-112 cells, which strongly decreased cell viability verifying the loss-of-function. (**Extended Data Fig. 7h**).

Analyzing LoF phenotypes in the FGFRi resistance screens also detected LoF mutations potentially indicating drug hypersensitivity. Many of these mutations also showed a LoF for FGFR activation and thus likely have an overall negative impact on cell proliferation, while others displayed a specific effect only in the drug resistance screens (**Extended Data Fig. 7i**). A strong enrichment of hypersensitizing missense over synonymous mutations (358-fold for pemigatinib, 188-fold for futibatinib) as well as clustering of strong hypersensitizing mutations in few codons (*e.g.* FGFR4 R611, D612, R616, D630) corroborated that the effects were likely non-random. However, no hypersensitizing and activating mutations have been identified, so that they will likely be relevant only in co-occurrence with activating mutations or fusions.

In summary, these data show that the saturation screen can also be exploited to identify LoF mutations.

Amino acid properties and hydrophobicity in GoF and LoF mutations

Next, we investigated whether the change in hydrophobicity of the exchanged amino acid correlated with the effect of a mutation. To compare mutations at the same position that altered hydrophobicity to those that did not, we selected codons with at least two activating mutations in FGFR1-4 yielding 224 activating and 172 neutral mutations within these codons. Not the absolute difference in hydrophobicity differed between neutral and activating mutations, but the direction of the change: neutral mutations associated with increased hydrophilicity, while activating mutations rather changed to hydrophobicity (**Extended Data Fig. 9a,b**).

Extending this analysis to the entire dataset and distinguishing GoF, LoF and neutral mutations uncovered a general median trend of increasing hydrophobicity associated with stronger GoF, while decreasing hydrophobicity associated with LoF (**Extended Data Fig. 9c**). Activating mutations were most frequent for mutations from asparagine, lysine or isoleucine and to tyrosine, lysine or histidine, while inactivating mutations were most frequent for changes from tryptophan, glycine and arginine and to tryptophan, proline and glycine (**Extended Data Fig. 9d**).

In summary, mutations introducing hydrophobicity were more likely GoF, while mutations introducing hydrophilicity were more likely LoF, but with different contributions of individual amino acids.

To assess whether this phenomenon could be widespread and to validate the result with an independent approach, we analyzed all 3257 recurrent missense mutations (frequency n>=2 in COSMIC v101) in the twelve RTKs mentioned above (**Extended Data Fig. 8**). For all twelve RTKs (Miyazawa hydrophobicity scale) or eleven out of twelve RTKs (Kyte-Doolittle hydrophobicity scale), the median difference between the mutant minus the WT amino acid hydrophobicity was positive, indicating a trend towards higher hydrophobicity caused by recurrent mutations (**Extended Data Fig. 9e-f**). Notably, also the bigger hydrophobicity differences showed a strong trend: while 1150 (Miyazawa) and 1229 (Kyte-Doolittle) mutations showed an increase in hydrophobicity by at least +1, only 323 (Miyazawa) and 766 (Kyte-Doolittle) mutations showed a decrease by at least -1. Assuming that recurrent cancer-associated mutations were likely oncogenic and hence gain-of-function, this analysis independently corroborated that GoF mutations were enriched in hydrophobicity-increasing mutations across twelve RTK proteins.

Reference numbers in the Supplementary Results section refer to references in the Main Text.

Supplementary Methods

Cell culture

HEK293T cells were maintained in DMEM + GlutaMax (Thermo Fisher Scientific) + 10% FBS + 4mM L-glutamine. NCI-H1581 cells were maintained in DMEM:F12 1:1 + GlutaMax + 10% FBS. RT-112 cells were maintained in RPMI 1640 + GlutaMax + 10% FBS. MCF10A cells were maintained under normal conditions in full medium consisting of DMEM:F12 1:1 + GlutaMax + 100 ng/ml cholera toxin + 20 ng/ml epidermal growth factor (EGF) + 0.01 mg/ml insulin + 500 ng/ml hydrocortisone + 5% horse serum. To assay growth factor independent proliferation, MCF10A cells were maintained in assay medium consisting of DMEM:F12 1:1 (PanBiotech) + 100 ng/ml cholera toxin + 500 ng/ml hydrocortisone + 2% horse serum. NIH-3T3 cells were cultured in RPMI 1640 + GlutaMax + 10% bovine serum (BS). Phoenix Eco were cultured in DMEM + GlutaMax + 10% FBS. All media supplements were purchased from Sigma / Merck KGaA unless specified otherwise.

All cell lines were cultured in standard incubators at 37°C with 5% CO_2, were routinely checked to be mycoplasma free and authenticated using the Multiplex Cell Authentication service (Multiplexion). **Supplementary Table 12** lists all cell lines and their origin/reference used in this study.

Production of lentivirus and transduction

HEK293T cells were seeded at a density of $52*10^4$ cells/cm^2 (6-well: $5*10^5$ cells/well, 10 cm dish: $3*10^6$ cells/dish) one day prior to transfection with lentiviral plasmids (transfer plasmid, psPAX2 packaging and pMD2.G envelope plasmid at a ratio of 4:2:1) using PEI (Sigma, Merck KGaA). Plasmids were diluted in Opti-MEM (Gibco) and mixed with PEI at a ratio of 1.5:1 (with a total of 2 µg DNA/well of a 6-well plate), immediately vortexed for 15 s and incubated at room temperature for 15 min. Normal medium was replaced with Opti-MEM and complexes were added on top. 12-18 h after transfection, the transfection medium was replaced with Opti-MEM. Viral supernatants were collected after 24 h and 48 h, pooled, filtered using a 0.45 µm filter (Sigma / Merck KGaA), snap frozen and stored at -80°C.

Cells were transduced with lentiviruses at 40-60% confluency in the presence of 8 µg/ml polybrene (Sigma / Merck KGaA). Transduction medium was replaced with normal medium after 24 h.

Retrovirus production and NIH-3T3 transduction

Plasmids carrying *FGFR* variants were transfected into Phoenix Eco using Lipofectamine 2000. Supernatant containing ecotropic retrovirus particles was harvested 2-3 days post-transfection, sterile-filtered, and used for infection of NIH-3T3 assisted by 4 µg/ml polybrene. NIH-3T3 carrying *FGFR* overexpression constructs were subsequently sorted to purity based on translationally coupled eGFP expression.

Cloning and mutagenesis of single point mutations

Primers used for cloning and mutagenesis and resulting plasmids are listed in **Supplementary Tables 9 and 13**, respectively. *FGFR1-4* cDNA for overexpression of wildtype cds were synthesized from TWIST Bioscience as clonal genes (for transcript IDs: see **Supplementary Table 8**) and cloned into the pHAGE backbone harboring an EF1α promoter, a puromycin cassette and 3'LTRs for the human cell lines MCF10A and NCI-H1581. For validation in NIH-3T3 cells, *FGFR2* cDNA was amplified from the source plasmid listed in **Supplementary Table 13** and cloned into EcoRI-digested pMIGR1 using Gibson assembly. Single point mutations coding for *FGFR* variants were introduced into the respective plasmids by site-directed mutagenesis (Q5® Site-Directed Mutagenesis Kit, New England Biolabs) according to the manufacturer's protocol. Successful mutagenesis was verified using Sanger sequencing of individual clones with four different primers to cover the full length of the FGFR cds. 1000 ng of plasmid DNA was also digested with BsrGI and PvuI as a quality control for each individual mutant plasmid. All mutations tested for this project have been included into the manuscript.

Cell viability assay for validation of activating mutations

MCF10A cells were seeded in full medium for transduction into 6-well plates with a density of $9.3x10^3$ cells/cm^2 (90000 cells/well) and transduced with an MOI of $\leq$0.3 as described above. Viruses used for validation experiments harbored either *FGFR1/2/3/4* WT, *FGFR1/2/3/4* point mutation or empty vector constructs and were produced as described above. After antibiotic selection with puromycin (3.5 µg/ml), cells were split once. When the cells reached a confluence of 60-90%, they were washed, trypsinized, centrifuged (200g, 5 min), resuspended with assay medium and seeded into new 6-well plates at a density of $9.3x10^3$ cells/cm^2 (90000 cells/well) or where indicated $7.8x10^3$ cells/cm^2 (75000 cells/well) for 96 h. Thereafter, the cells were washed, trypsinized, centrifuged and resuspended in 900 µl assay medium. 60 µl of this cell suspension was added to each well of a 96-well plate and 40 µl assay medium was added to each well. 96-well plates were incubated for further 72 h and finally analyzed using CellTiter-Glo® Luminescent Cell Viability Assay (Promega) according to the manufacturer's recommendations but with the reagent diluted 1:4 with PBS prior to use. Per cell line, four technical replicates per biological replicate and n≥3 biological replicates were prepared.

Cell viability assay for validation of resistance mutations

NCI-H1581 cells were seeded into 96-well plates at a density of $1.56x10^4$ cells/cm^2 (5000 cells/well). RT-112 cells were seeded into 96-well plates at a density of $1.25x10^4$ cells/cm^2 (4000 cells/well). RT-4 cells were seeded into 96-well plates at a density of $1.88x10^4$ cells/cm^2 (6000 cells/well). To add the indicated inhibitor after 24 h, the medium was aspirated and fresh medium with a final inhibitor concentration of 20 nM pemigatinib or futibatinib was added to the cells. After incubation of the cells for 72 h, cells were washed, trypsinized, resuspended and again treated with medium harboring the same inhibitor concentration. After another 24 h incubation, analysis using the CellTiter-Glo® Luminescent Cell Viability Assay (Promega) according to the manufacturer's recommendations was performed but with the CTG reagent

being diluted 1:4 with PBS prior to use. Per cell line four technical replicates per biological replicate and n≥3 biological replicates were prepared.

Cell viability assay for validation of endogenous LoF mutation

RT-112 wildtype and the RT-112 K508M LoF mutant cells were seeded into 96-well plates at a density of 1.56x10^4 cells/cm^2 (5000 cells/well). After incubation of the cells for 72 h, analysis using the CellTiter-Glo® Luminescent Cell Viability Assay (Promega) according to the manufacturer's recommendations was performed but with the CTG reagent being diluted 1:4 with PBS prior to use. Per cell line, four technical replicates per biological replicate and three biological replicates were prepared.

NIH-3T3 low-serum proliferation assay

NIH-3T3 cells expressing *FGFR* variants were seeded into 96-well plates at a density of 5000 cells per well in RPMI 1640 + GlutaMax supplemented with 1% Penicillin/Streptomycin and serum concentrations ranging from 0% to 5% BS. After incubation for 7 days, the viability of cells was measured with ATPlite 1step Luminescence Assay System (Revvity) according to the manufacturer's recommendations. Data was background corrected, normalized to the signal obtained from wells with 5% BS and the R package "drc"[1] was used to analyze data with a two-parameter log-logistic dose response model.

NIH-3T3 TKI sensitivity profiling

NIH-3T3 cells expressing *FGFR* variants were seeded into 96-well plates at a density of 4500 cells per well in RPMI 1640 + GlutaMax supplemented with 1% Penicillin/Streptomycin and 1.5% BS. 10 mM inhibitor stock solutions in DMSO were serially diluted and added to achieve final concentrations ranging from 5000 nM to 0.25 nM. The following inhibitors were used: AZD4547 (TargetMol), erdafitinib (TargetMol), futibatinib (MedChemExpress), infigratinib (TargetMol), pemigatinib (TargetMol) and zoligratinib (MedChemExpress). After incubation for 5 days, the viability of the cells was measured with the cell proliferation reagent WST-1 (Roche). Data was background corrected, normalized to the signal obtained from wells with no inhibitor and the R package "drc"[98] was used to analyze data with a four-parameter log-logistic dose response model.

NIH-3T3 soft agar assay

Medium for soft agar assays was RPMI 1640 + GlutaMax supplemented with 1% Penicillin/Streptomycin and 10% BS. A base layer containing 0.7% agarose was poured into 6 well-plates and solidified. NIH-3T3 cells expressing *FGFR* variants were seeded at a density of 5000 cells per well into a semi-solid top layer containing 0.4% agarose. The top agar was covered with medium which was exchanged after 7 days. After incubation for 14 days, colonies were stained with 1 mg/ml nitroblue tetrazolium chloride solution overnight, imaged, and the area covered by colonies measured with ImageJ plugin ColonyArea[99]. Area measurements from individual wells were normalized to control wells of NIH-3T3 *FGFR2*_WT from the same plate.

CRISPR/Cas based genome editing

First, the wild-type genomic DNA sequence covering a 1-kb region around the intended site of mutagenesis in the cell lines RT-112 and RT-4 was verified by Sanger sequencing. To introduce individual mutations endogenously, a CRISPR/Cas9 homology directed repair (HDR) based precision genome editing system was used. The required sgRNAs and ssODNs were either custom designed or designed using the Alt-R™ HDR Design Tool (IDT). sgRNAs and ssODNs were synthesized at IDT and sequences as well as specifications are listed in **Supplementary Tables 14 and 15**, respectively. Ribonucleoprotein particles (RNPs) were formed by combining sgRNA and SpyCas9 (EnGen® Spy Cas9 NLS, New England Biolabs) according to IDT guidelines. RNPs were delivered by nucleofection using the Amaxa 4D-Nucleofector device (Lonza). For both cell lines, the cells were grown to 70-80% confluency, then trypsinized, counted and $4x10^5$ cells per reaction were centrifuged at 100g for 5 min at room temperature. RT-112 cells were resuspended with 20 µl SE and RT-4 cells with 20 µl SG cell line buffer. 1 µl of ssODNs (100 µM) and afterwards the pre-prepared RNP complexes were added to the mixture. The final mixture was mixed carefully by pipetting up and down and transferred to a 16-well Nucleocuvette™ strip. Cells were nucleofected using the SG Cell Line 4D-NucleofectorTM X Kit (Lonza) for RT-4 and the SE Cell Line 4D-NucleofectorTM X Kit (Lonza) for RT-112 with the DS-126 program by the Amaxa 4D-Nucleofector device (Lonza). Afterwards, cells were incubated for 10 min at room temperature and carefully resuspended with 20 µl of medium spiked with 3 mM Alt-R HDR Enhancer V2 (IDT). The whole mixture was then added to a 12-well plate containing 1 ml of medium. Cells were incubated for 48-72 h until confluent and afterwards trypsinized. Part of the cell suspension was used for gDNA isolation using the DNeasy Blood & Tissue Kit (Qiagen) and the respective locus was amplified via PCR (Q5® High-Fidelity DNA Polymerase, New England Biolabs). Bulk Sanger sequencing and ICE analysis were performed to determine editing efficiencies for each mutation. For each mutation, only the sgRNA with the highest HDR efficiency was further processed. The remaining cells were seeded into 96-well plates for single cell isolation. When cells were 50-80% confluent, gDNA was isolated using the Quick-DNA 96 Kit (Zymo Research). The target sequence of each clone was amplified (Q5® High-Fidelity DNA Polymerase, New England Biolabs) and purified using AMPure XP magnetic beads (Beckman Coulter). Afterwards, 96-well Sanger sequencing was performed to determine the genotypes and identify homozygously or heterozygously edited clones. The remaining clones were expanded, the mutation was validated by another round of Sanger sequencing and the cells were subsequently used to determine cell viability for validation of resistance/LoF mutations as described above.

Cell lysis, western blotting and image quantification

For activating and LoF mutations, MCF10A cells were transduced with an MOI$\leq$0.3 as described above and seeded into 10 cm dishes with a density of $1.59x10^4$ cells/cm^2 ($9x10^5$ cells/plate) using full medium and incubated for 48 h. For resistance mutations, NCI-H1581 cells were transduced with an MOI$\leq$0.3 as described above and seeded into 10 cm dishes with a density of $1.06x10^5$ cells/cm^2 ($6x10^6$ cells/plate). After 24 h, medium was replaced with medium

containing the respective inhibitors or DMSO and the cells were incubated for 4 h. After incubation, cells were washed once with PBS and scratched with a cell scraper using 5 ml ice-cold PBS. Next, cells were centrifuged at 4200g at 4°C and the pellet was snap frozen. RIPA buffer (150 mM NaCl, 50 mM Tris pH 7.4, 0.1% SDS, 0.5% Sodium deoxycholate, 1% Triton X-100) was added in a 1:1 ratio to the cell pellet and incubated on ice for 50 min. Tubes were mixed by gently flicking the tube every 10 min and centrifuged afterwards for 30 min at 16200g at 4°C. The supernatant was transferred to a fresh 1.5 ml reaction tube and protein concentrations were determined using the Pierce™ BCA Protein Assay Kits (Thermo Fisher Scientific). Per sample, 6 µg protein for activation and LoF mutations or 10 µg protein for resistance mutations were mixed with RIPA buffer and 6x SDS loading buffer (0.5 M Tris (pH 6.8), 50% glycerol, 10% DTT (Dithiothreitol), 0.001% Bromophenol blue, 4% SDS) and incubated at 95°C for 10 min. Samples were loaded onto an 8% SDS-PAGE gel and the transfer was carried out for 80 min at 80 V onto an activated 0.45 µM PVDF membrane. Blocking was performed at room temperature for 60 min using 5% Bovine Serum Albumin (BSA) in TBST (20 mM Tris-Cl, pH 7.6, 158 mM NaCl, and 0.1% Tween-20). For the primary antibodies, membranes were cut and incubated with a 1:1000 dilution (except beta-Actin: 1:10000) with 5% BSA in TBST of the respective antibody at 4°C moving overnight. On the next day, the secondary antibodies were diluted 1:10000 with 5% BSA in TBST and blots were incubated with the secondary antibodies for 60 min moving at room temperature. After each antibody incubation, membranes were washed three times with TBST. For visualization, the Cytiva Amersham™ ECL™ Prime Western-Blot-Detection-Reagent and ChemoCam Imager (INTAS) were used. Antibodies used in this study are listed in **Supplementary Table 16**.

Odds Path Analysis

CKB and OncoKB provided a classification for a small subset of mutations, namely 136 (CKB) and 118 (OncoKB) missense mutations in the kinase domains of FGFR1-4. We used these classifications for an odds path analysis guided by the “decision tree for the evaluation of functional data for clinical variant interpretation”[100] to assess the results of our screening assay. As pathogenic classified variants regarding FGFR activation, we used mutations annotated as “gain of function” or “gain of function - predicted” (CKB) or “gain-of-function” or “likely gain-of-function” (OncoKB), respectively. As benign classified variant regarding FGFR activation, we used mutations annotated as “no effect - predicted”, “loss of function - predicted” or “loss of function” (CKB) or “likely neutral” or “likely loss-of-function” (OncoKB), respectively. These were then compared to functionally abnormal, *i.e.* activating, mutations according to our screen (median enrichment >=1.5) versus functionally normal, *i.e.* non-activating, mutations (median enrichment <1.5). The odds path analysis[100] then determined an evidence strength equivalent of PS3/BS3. The calculations are presented in **Supplementary Table 3**.

External data sources

Data from the Clinical Knowledgebase (CKB) were obtained from https://ckb.jax.org/ and downloaded on February 7th, 2024, for *FGFR1, FGFR2, FGFR3* and *FGFR4*. OncoKB data were

obtained from https://www.oncokb.org/ and downloaded on October 23rd, 2023, for *FGFR1, FGFR2, FGFR3* and *FGFR4*. Comparison to the screen data was performed via matching of the amino acid mutation. SNV allele frequencies were obtained from gnomAD at http://gnomad.broadinstitute.org/ (v3.1.2, non-cancer, GRCh38) on January 19th, 2023[101]. The SNV frequencies of cancer samples were obtained from COSMIC at https://cancer.sanger.ac.uk/cosmic (v97, GRCh38) on January 19th, 2023. This included the following datasets: *FGFR1* (ENST00000447712.6), *FGFR2* (ENST00000358487.9), *FGFR3* (ENST00000440486.7) and *FGFR4* (ENST00000292408.8). Comparison with the screen data was conducted by matching the nucleotide mutation for gnomAD and COSMIC samples. The tissue origin data for the COSMIC samples were obtained on November 28th, 2023, from https://cancer.sanger.ac.uk/cosmic/download/cosmic#cosmic and generated by combining the Genome Sequencing, Targeted Sequencing and Classification data (v99, GRCh38). For the hydrophobicity analysis of the twelve RTKs, data was downloaded from COSMIC on March 14th, 2025 (v101, GRCh38) for the respective transcript isoform with most simple mutations for each gene. To determine the prediction scores for the FGFR mutations examined in this study, the algorithms REVEL[102], EVE[103] and AlphaMissense[104] were applied.

Mapping of screen data to FGFR kinase domain structures and structural prediction of mutant FGFRs

FGFR structure files for mapping of screen data were either derived from PDB[105] (7WCL, https://www.rcsb.org/) or predicted using AlphaFold2. For the structural alignment, the screen data were first aggregated for each amino acid by calculating the median of the screening results for all missense mutations. For the LoF structural analysis, the data were aggregated again at the amino acid level for the respective minimum value of all missense mutations. These values were then plotted in color onto the structure according to the effect strength. Amino acid sequences with individual mutations were predicted on Galaxy[106] using the AlphaFold2[107] algorithm and visualized with PyMOL (The PyMOL Molecular Graphics System, Version 3.0 Schrödinger, LLC).

Determination of hydrophobicity properties

The hydrophobicity of each amino acid was assigned using the Miyazawa[108] (https://web.expasy.org/protscale/pscale/Hphob.Miyazawa.html) or the Kyte-Doolittle[109] (https://web.expasy.org/protscale/pscale/Hydropath.Doolittle.html) hydrophobicity scales. For each mutation, the difference in hydrophobicity was determined by subtracting the hydrophobicity of the wildtype from that of the mutant.

Data analysis, statistics and graphics tools

The complete data for enrichment of each tested mutation including all replicates and significance values are available in **Supplementary Table 10**. Thresholds for p-values with Q = 1% applicable for screen data discoveries are available in **Supplementary Table 11**. For validation experiments, statistical significance was calculated using a two-tailed t-test, with variance of the different groups being determined using an F-test upstream of the t-test. For

Spearman correlation analysis, an approximate *P* value for nonparametric correlation was calculated using Prism. For the correlation between different cell lines, parametric Pearson correlation analysis was performed. *P* values appear as: ns = $P > 0.05$, * = $P \leq 0.05$, ** = $P \leq 0.01$, *** = $P \leq 0.001$, **** = $P \leq 0.0001$.
GraphPad Prism version 10.2.3 and Microsoft Excel version 16.86 was used for creating all graphs and for performing the statistical tests. Arrangement and combination of figure panels was carried out using Adobe Illustrator version 10.16.

Supplementary Methods References

98. Ritz, C. et al. Dose-Response Analysis Using R. PloS One **10**, e0146021 (2015).
99. Guzmán, C. et al. ColonyArea: an ImageJ plugin to automatically quantify colony formation in clonogenic assays. PloS One **9**, e92444 (2014).
100. Brnich, S. E. et al. Recommendations for application of the functional evidence PS3/BS3 criterion using the ACMG/AMP sequence variant interpretation framework. Genome Med. 12, 3 (2019).
101. Lek, M. et al. Analysis of protein-coding genetic variation in 60,706 humans. Nature **536**, 285–291 (2016).
102. Ioannidis, N. M. et al. REVEL: An Ensemble Method for Predicting the Pathogenicity of Rare Missense Variants. Am. J. Hum. Genet. 99, 877–885 (2016).
103. Frazer, J. et al. Disease variant prediction with deep generative models of evolutionary data. Nature 599, 91–95 (2021)
104. Tordai, H. et al. Analysis of AlphaMissense data in different protein groups and structural context. Sci. Data 11, 495 (2024).
105. Berman, H. M. et al. The Protein Data Bank. Nucleic Acids Res. **28**, 235–242 (2000).
106. Galaxy Community. The Galaxy platform for accessible, reproducible, and collaborative data analyses: 2024 update. Nucleic Acids Res. 52, W83–W94 (2024).
107. Jumper, J. et al. Highly accurate protein structure prediction with AlphaFold. Nature **596**, 583–589 (2021).
108. Miyazawa, S. & Jernigan, R. L. Estimation of effective interresidue contact energies from protein crystal structures: quasi-chemical approximation. Macromolecules **18**, 534–552 (1985).
109. Kyte, J. & Doolittle, R. F. A simple method for displaying the hydropathic character of a protein. J. Mol. Biol. **157**, 105–132 (1982).
