# Supplementary material for: Saturation mutagenesis identifies activating and resistance-inducing FGFR kinase domain mutations
Source: Nat Genet. 2025 Dec 8;58(1):157–68. doi: 10.1038/s41588-025-02431-8 (PMC12807871; doi:10.1038/s41588-025-02431-8)

<u>Raw Data of Western Blot Replicates</u>

BR = Biological Replicate

<u>Western blots MCF10A:</u>

Activation = Figure 2i

Activation BR1 (Blot 1, FGFR, FRS2, Actin)

Marker (FGFR2)

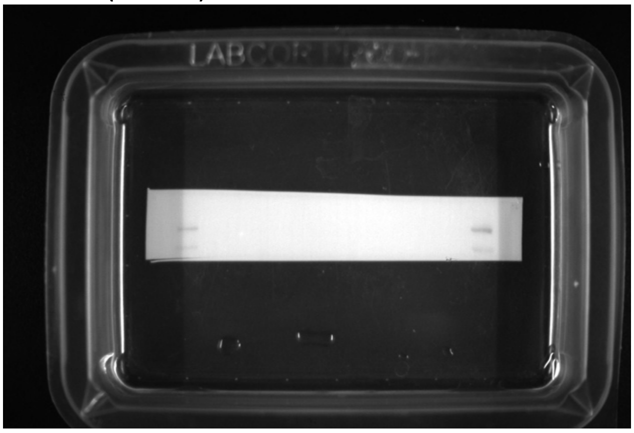

FGFR2

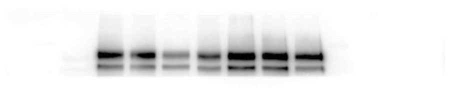

Marker (FRS2)

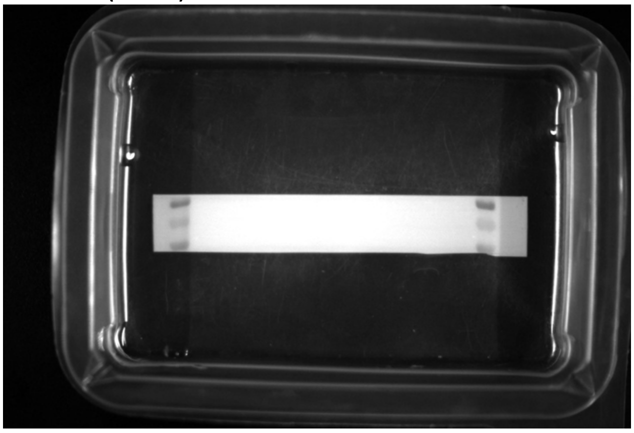

FRS2

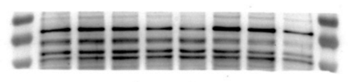

Marker (Actin)

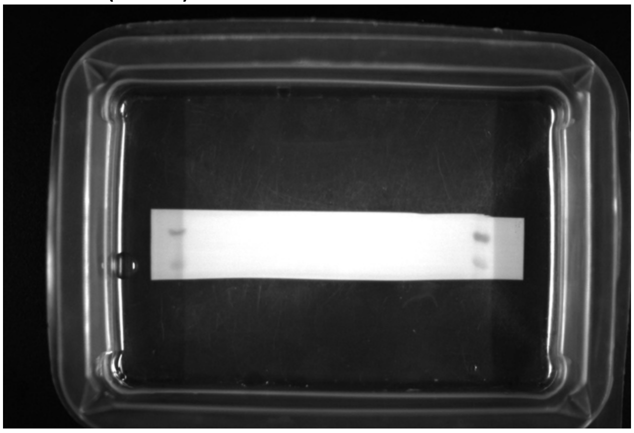

Actin

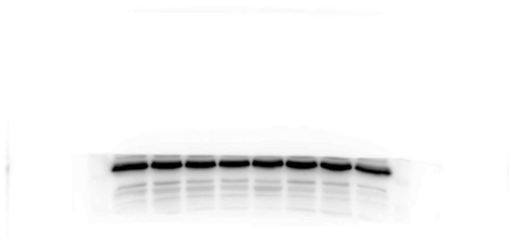

Activation BR1 (Blot 2, pFGFR, pFRS2, Actin)

Marker (pFGFR)

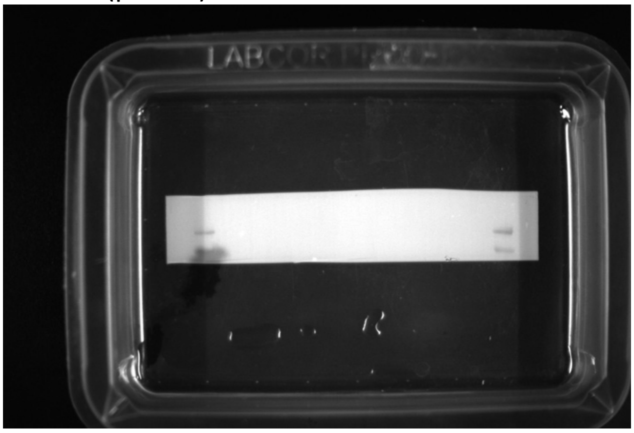

pFGFR

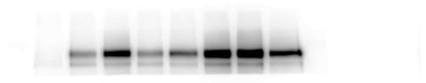

Marker (pFRS2)

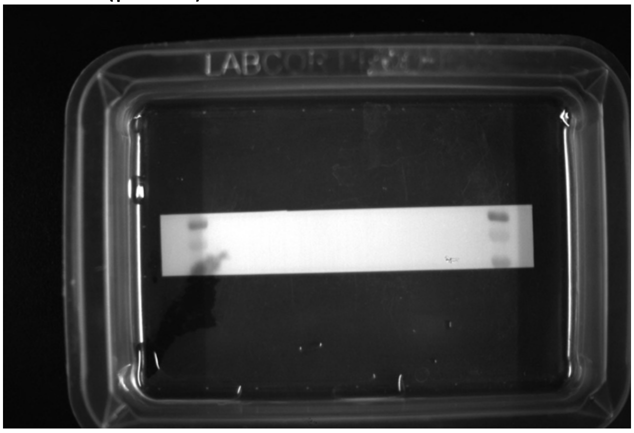

pFRS2

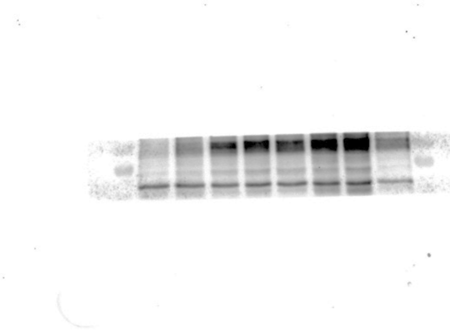

Marker (Actin)

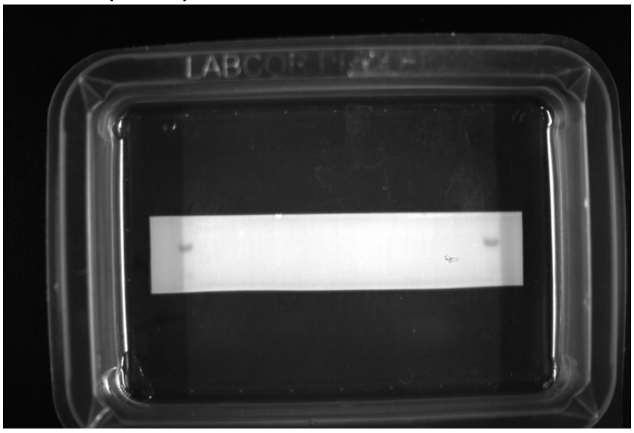

Actin

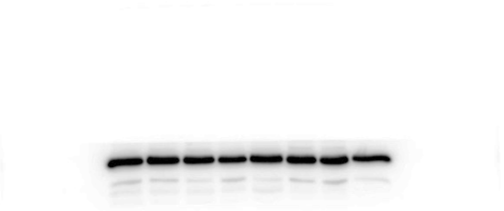

Activation BR2 (Blot 1, FGFR, FRS2, Actin)

Marker (FGFR2)

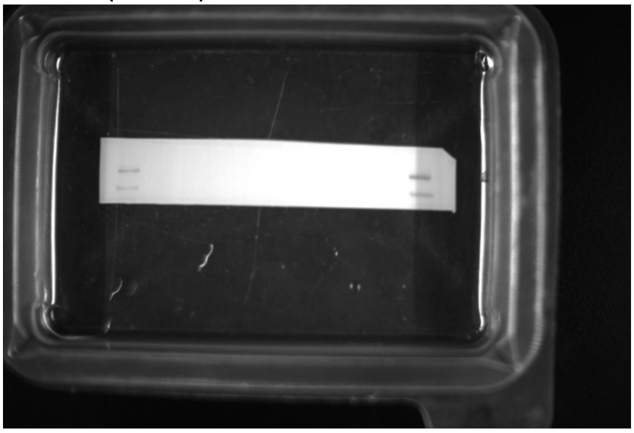

FGFR2

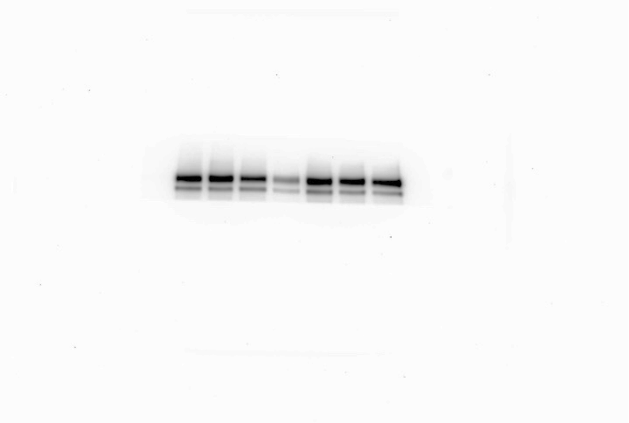

Marker (FRS2)

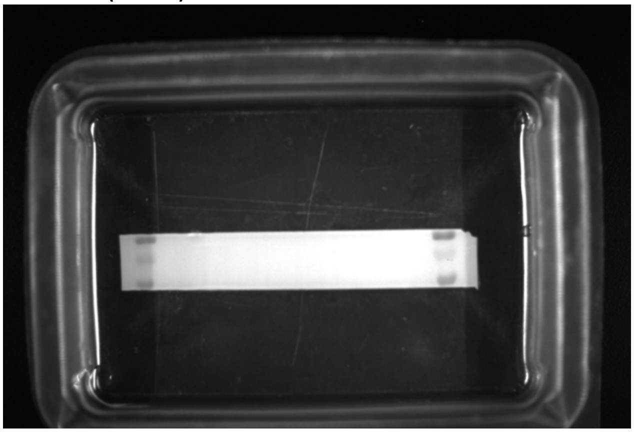

FRS2

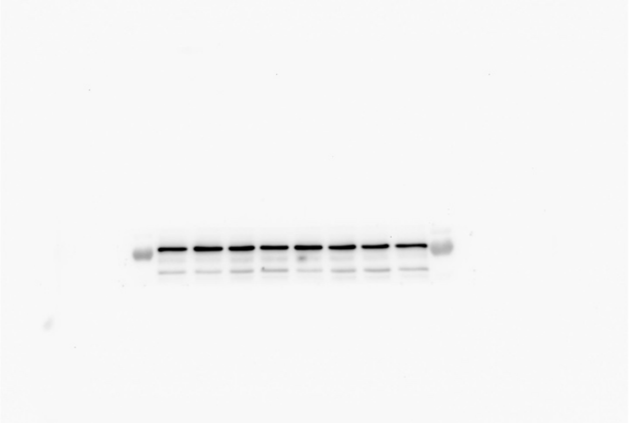

Marker (Actin)

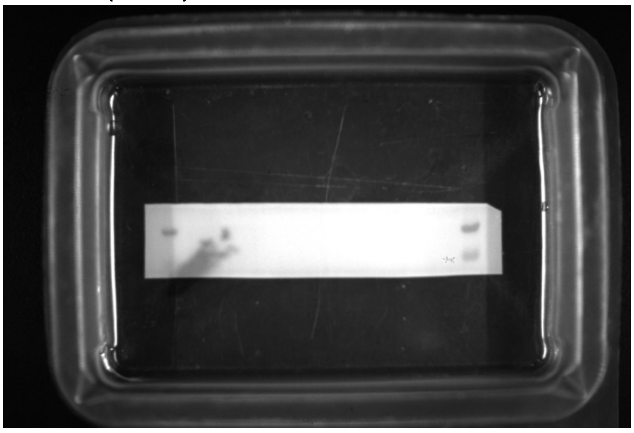

Actin

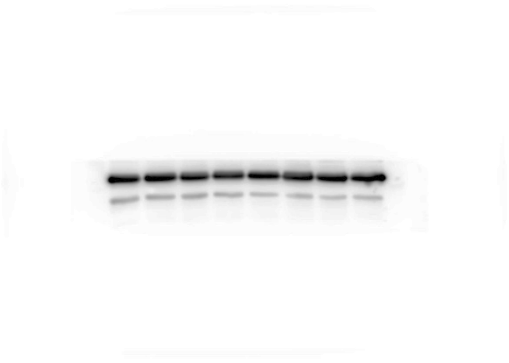

Activation BR2 (Blot 2, pFGFR, pFRS2, Actin)

Marker (pFGFR)

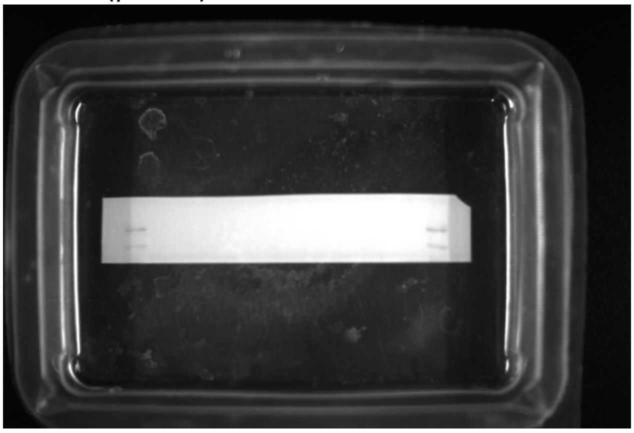

pFGFR

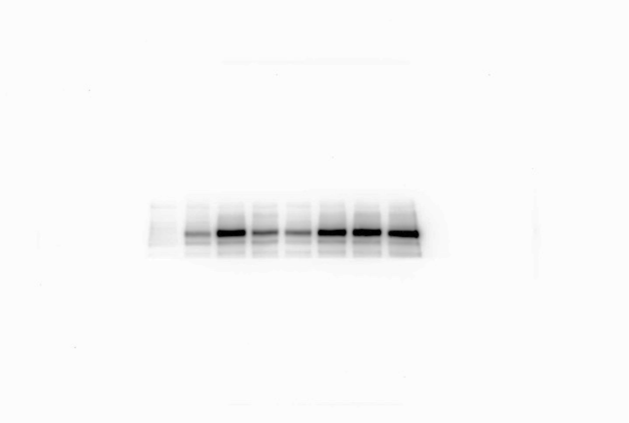

Marker (pFRS2)

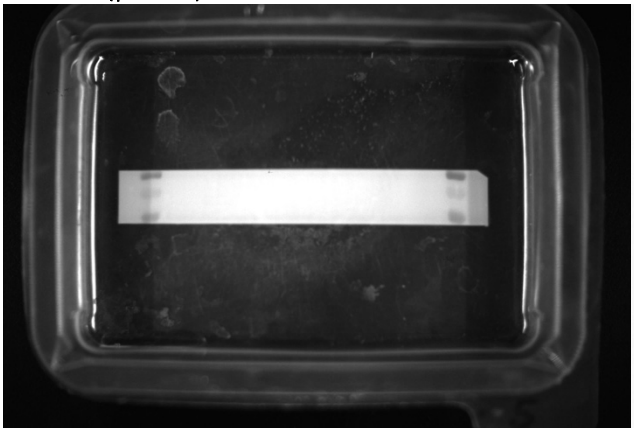

pFRS2

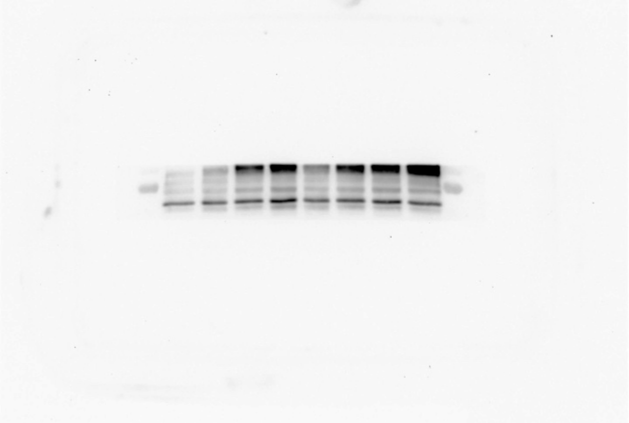

Marker (Actin)

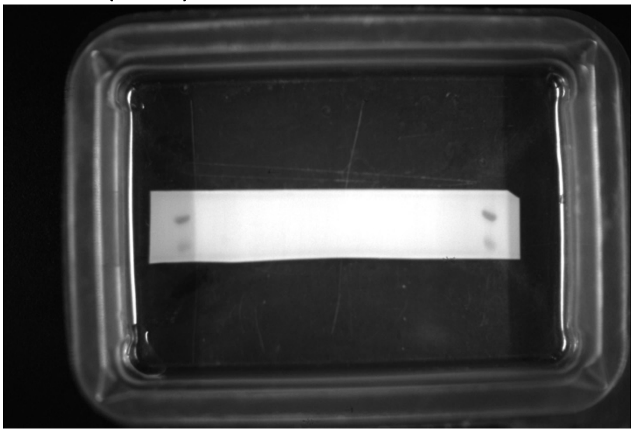

Actin

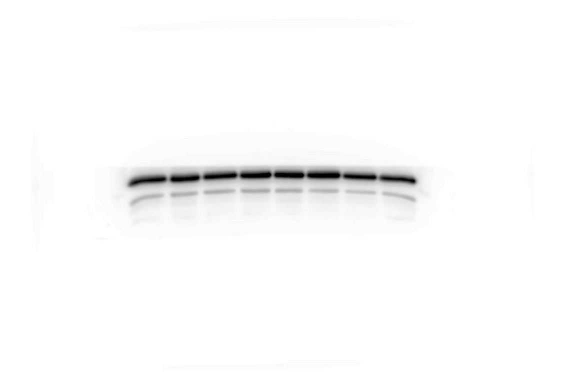

Activation BR3 (Blot 1, FGFR, FRS2, Actin)

Marker (FGFR2)

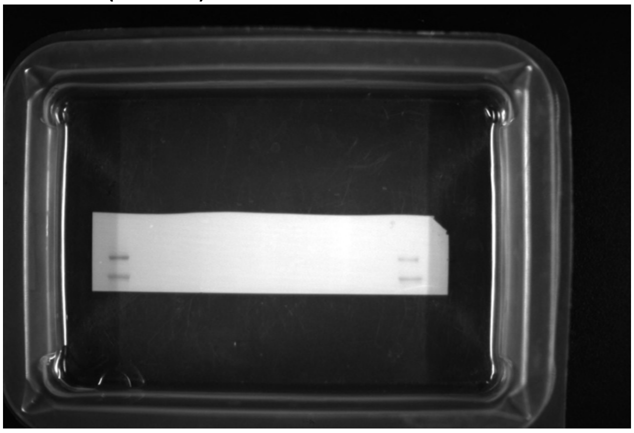

FGFR2

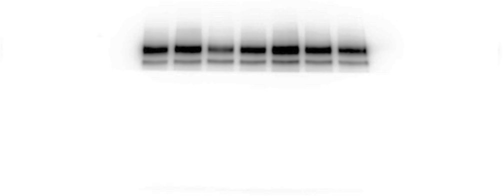

Marker (FRS2)

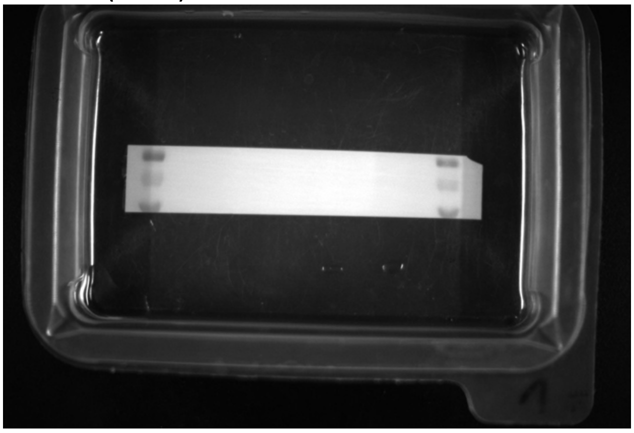

FRS2

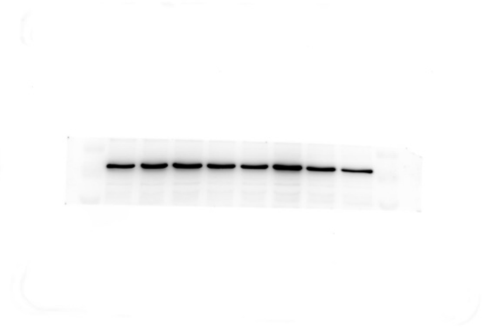

Marker (Actin)

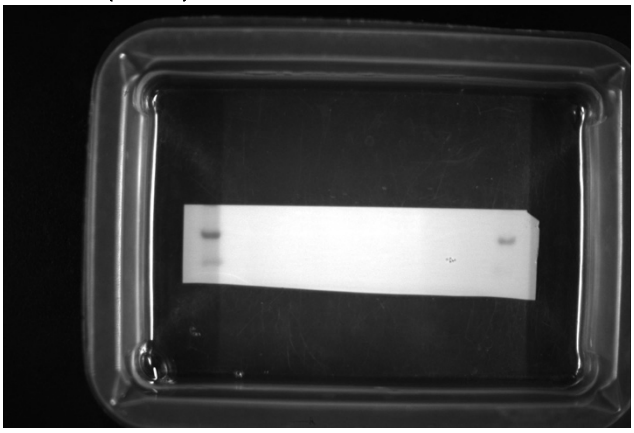

Actin

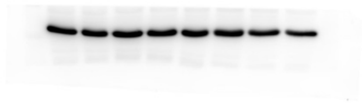

Activation BR3 (Blot 2, pFGFR, pFRS2, Actin)

Marker (pFGFR)

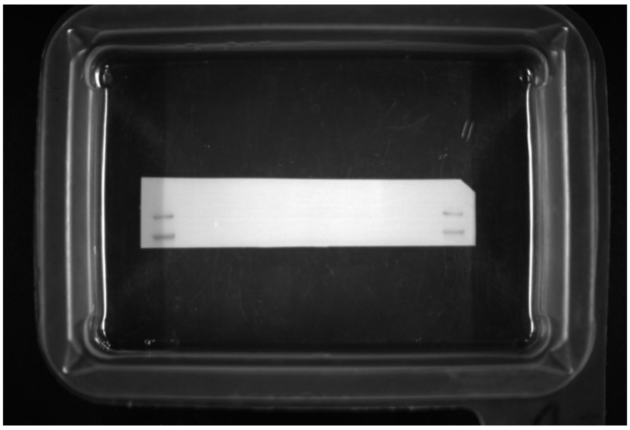

pFGFR

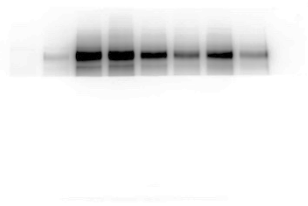

Marker (pFRS2)

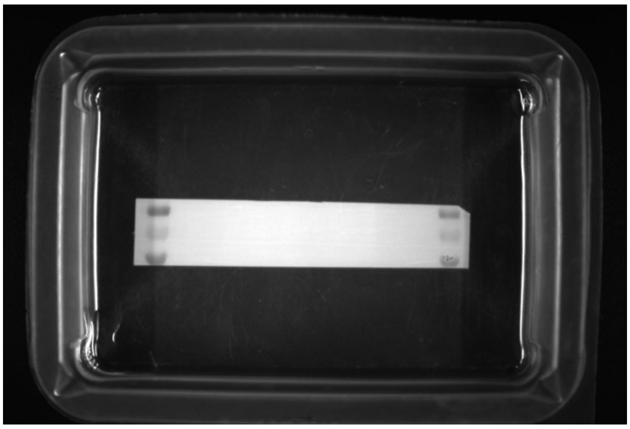

pFRS2

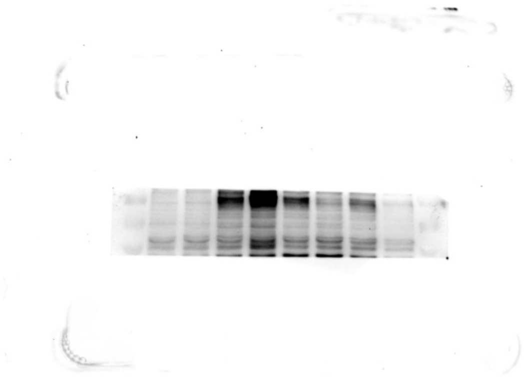

Marker (Actin)

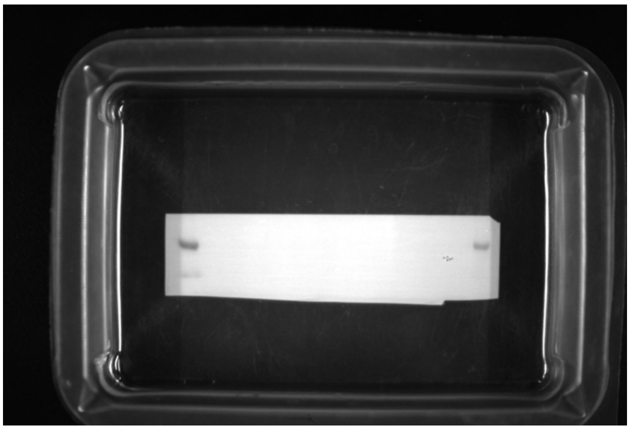

Actin

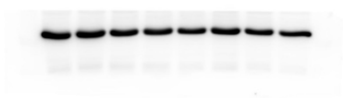

Loss-of-function mutations = Figure 4g

Loss-of-function BR1 (Blot 1, FGFR2, Actin)

Marker (FGFR2)

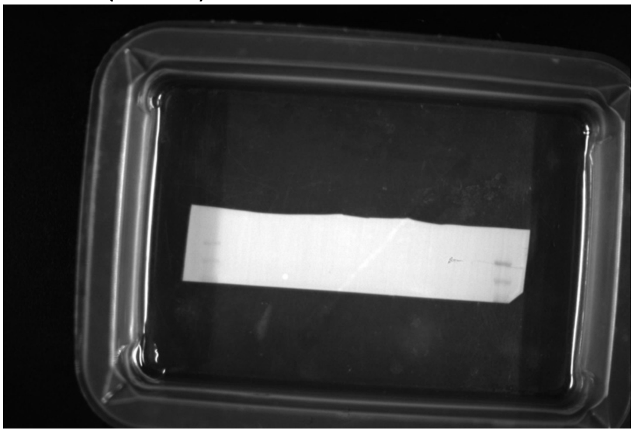

FGFR2

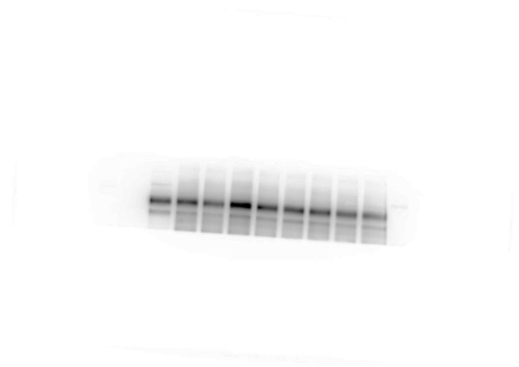

Marker (Actin)

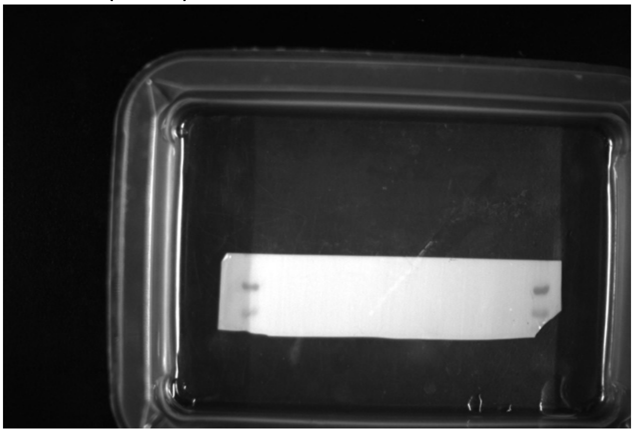

Actin

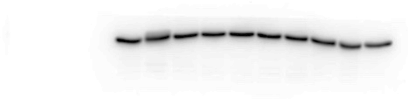

Loss-of-function BR1 (Blot 2, pFGFR2, Actin)

Marker (pFGFR2)

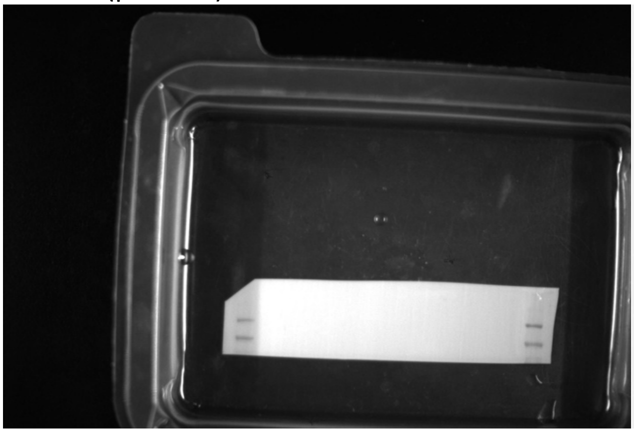

pFGFR2

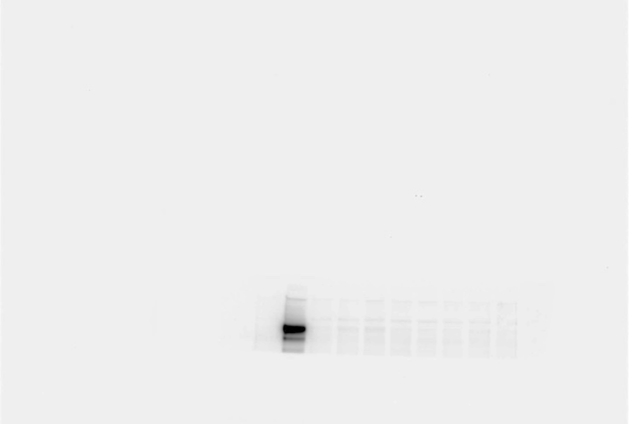

Marker (Actin)

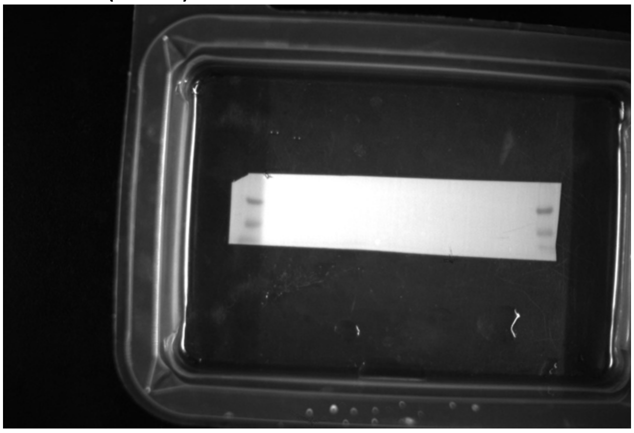

Actin

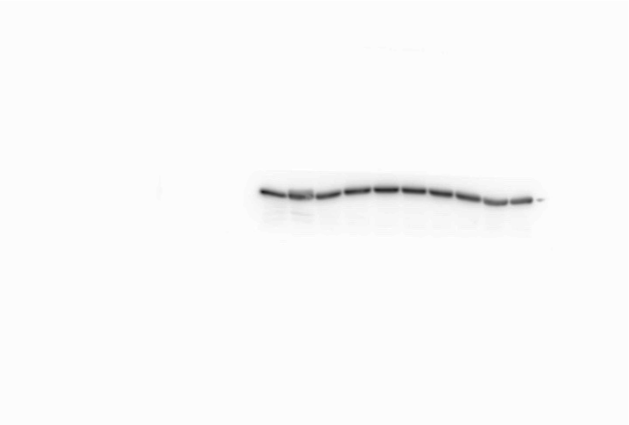

Loss-of-function BR2 (Blot 1, FGFR2, Actin)

Marker (FGFR2)

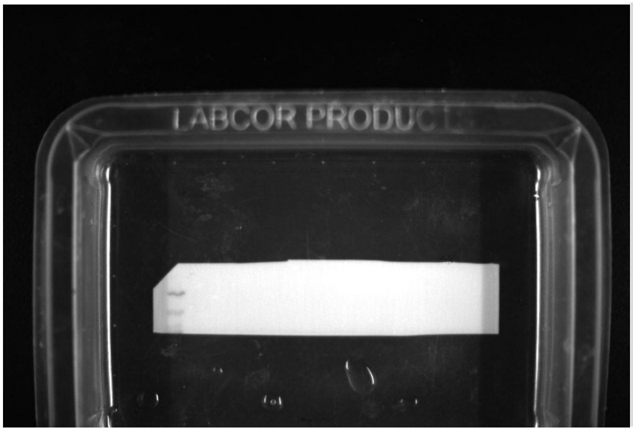

FGFR2

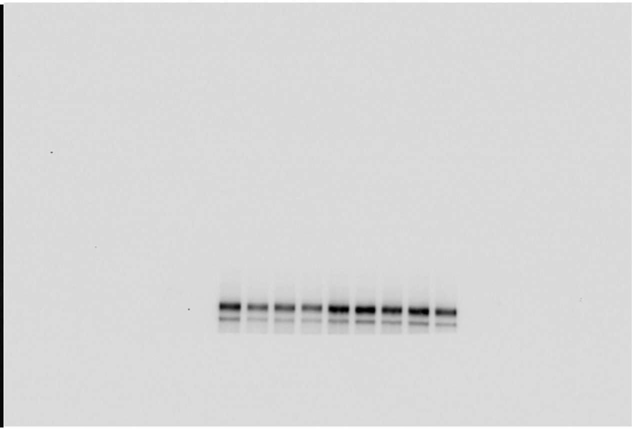

Marker (Actin)

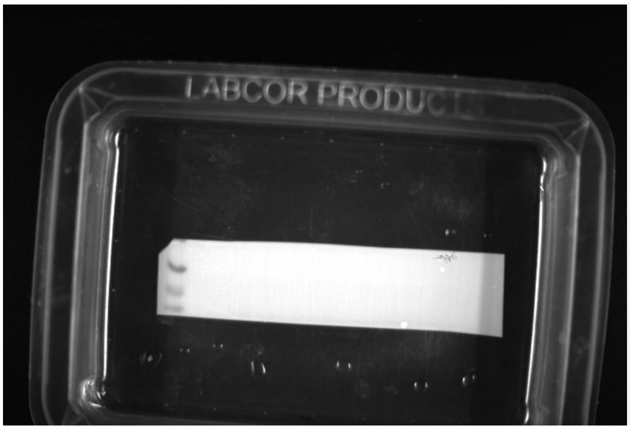

Actin

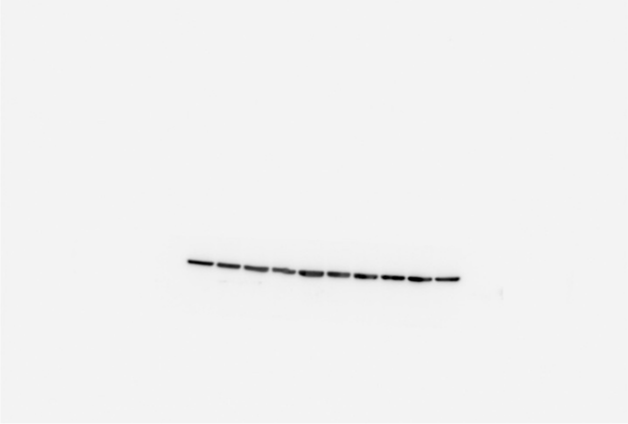

Loss-of-function BR2 (Blot 2, pFGFR2, Actin)

Marker (pFGFR2)

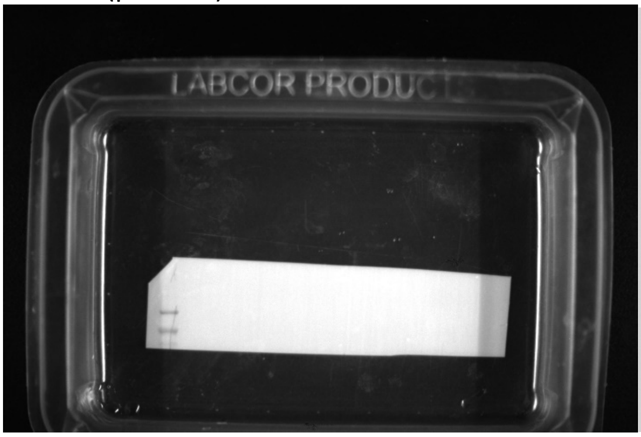

pFGFR2

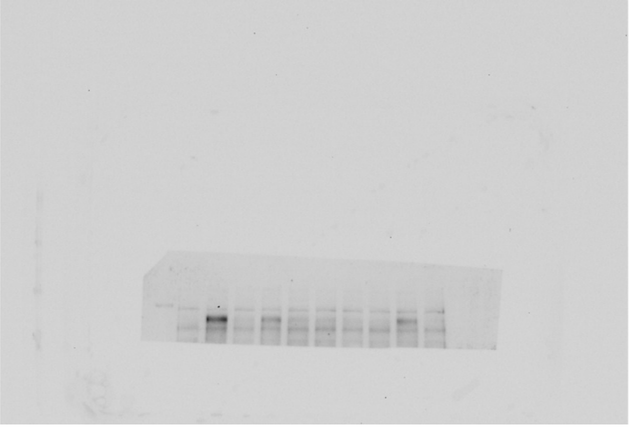

Marker (Actin)

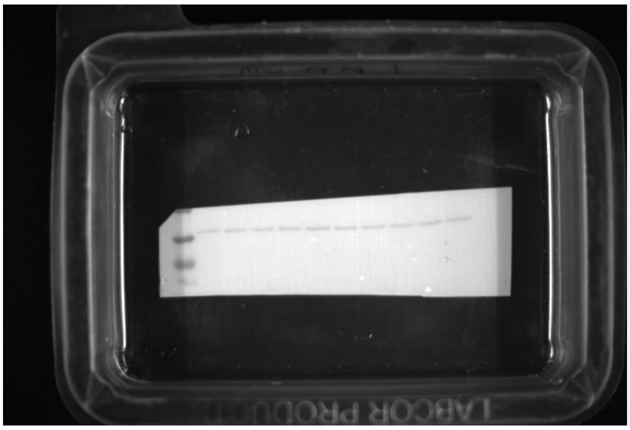

Actin

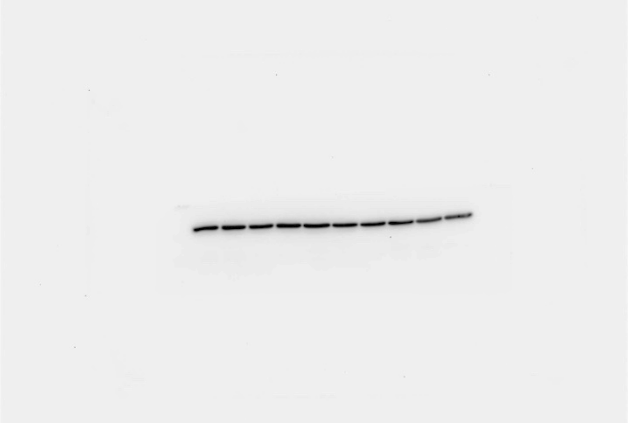

Loss-of-function BR3 (Blot 1, FGFR2, Actin)

Marker (FGFR2)

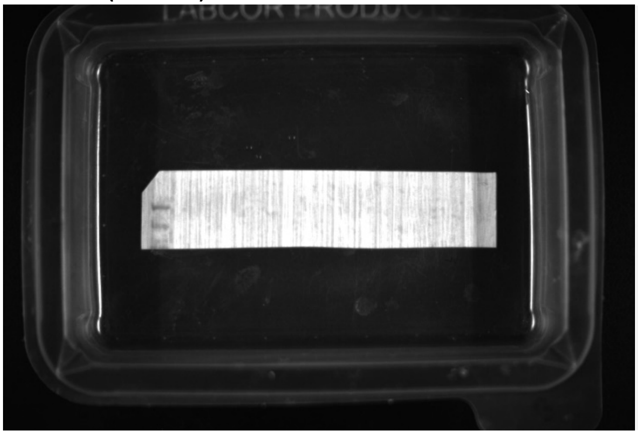

FGFR2

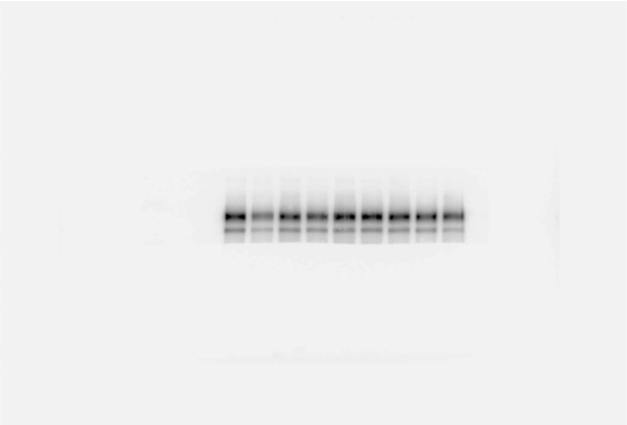

Marker (Actin)

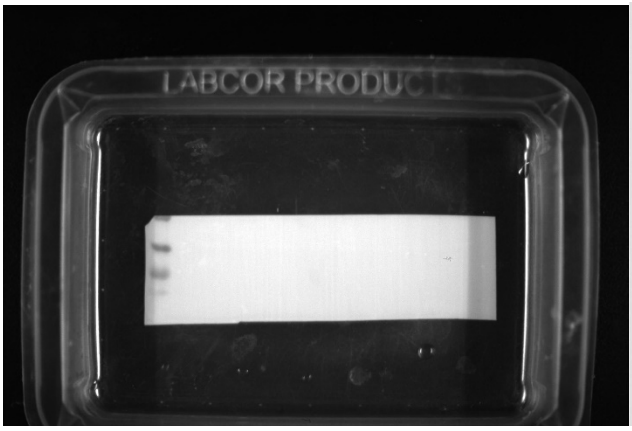

Actin

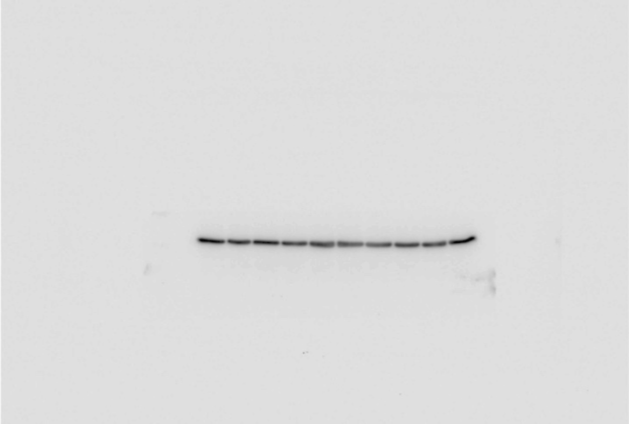

Loss-of-function BR3 (Blot 2, pFGFR2, Actin)

Marker (pFGFR2)

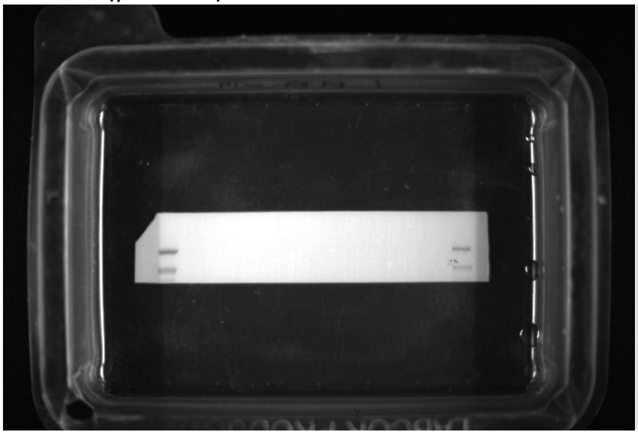

pFGFR2

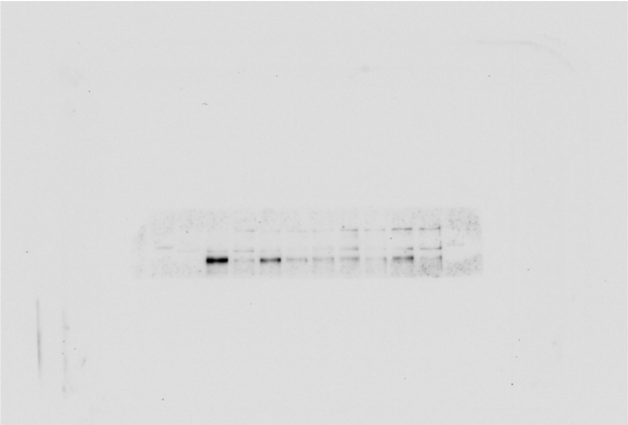

Marker (Actin)

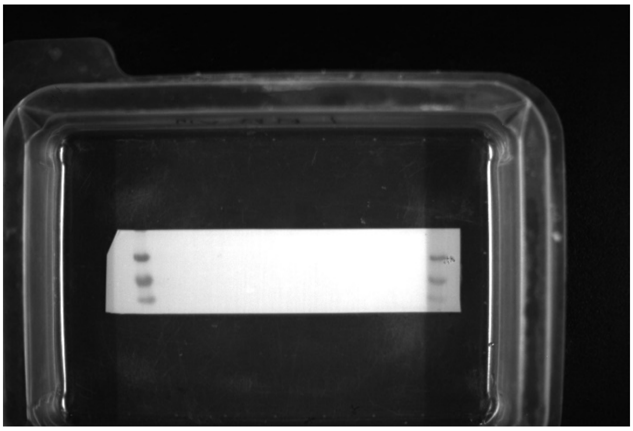

Actin

<u>Western blots NCI-H1581:</u>
Pemigatinib resistance = Figure 3f, left panel

Pemigatinib resistance NCI-H1581 BR1 (Blot 1, FGFR2, FRS2, Actin)

Marker (FGFR2)

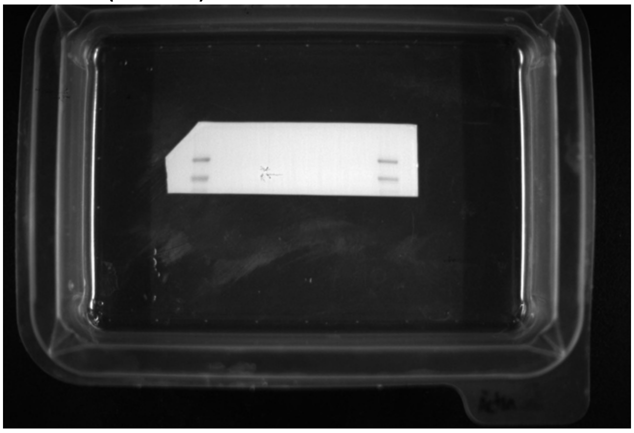

FGFR2

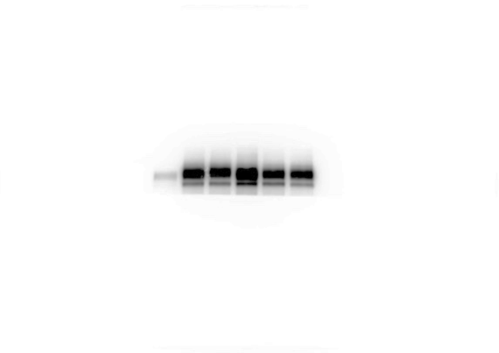

Marker (FRS2)

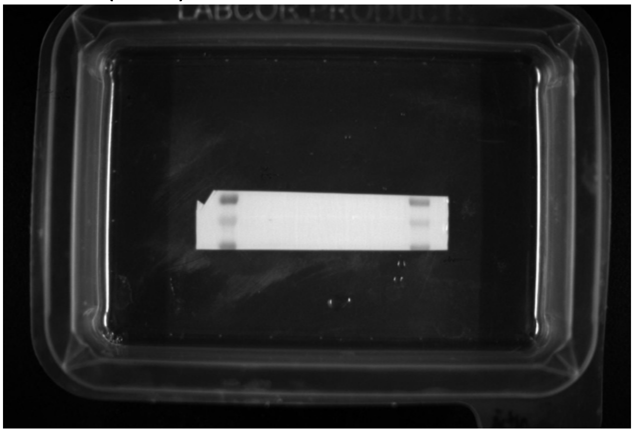

FRS2

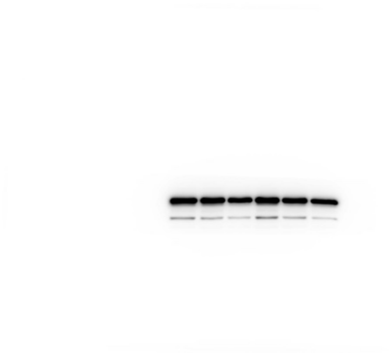

Marker (Actin)

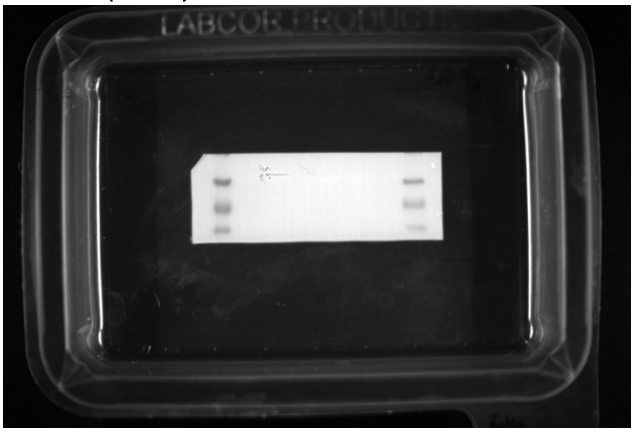

Actin

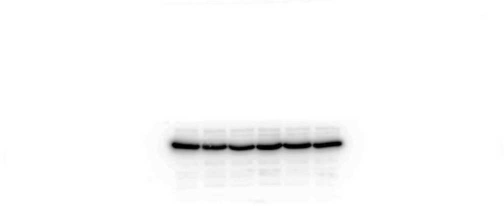

Pemigatinib resistance NCI-H1581 BR1 (Blot 2, pFGFR2, pFRS2, Actin)

Marker (pFGFR)

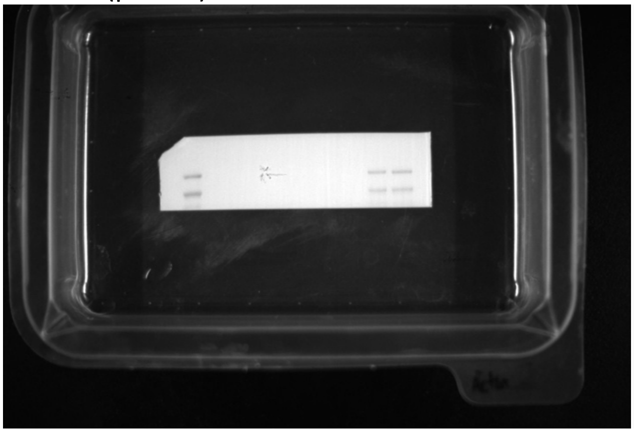

pFGFR

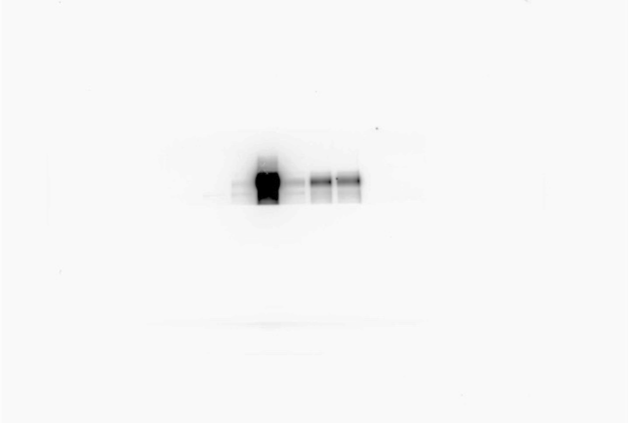

Marker (pFRS2)

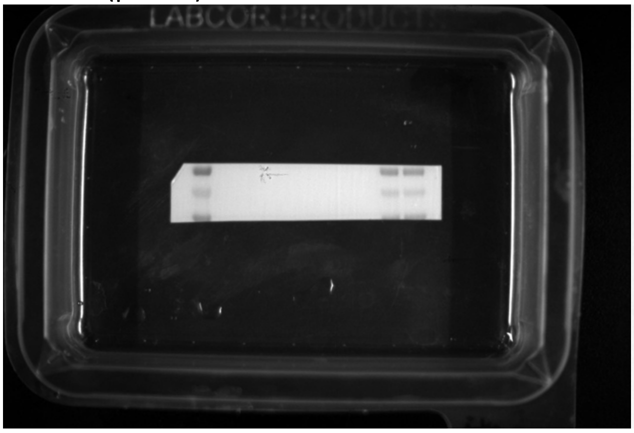

pFRS2

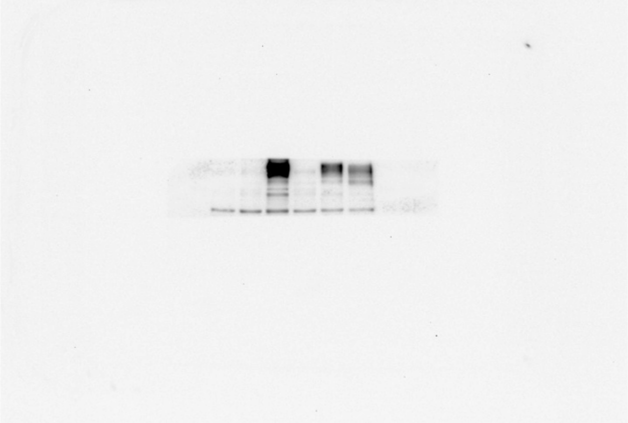

Marker (Actin)

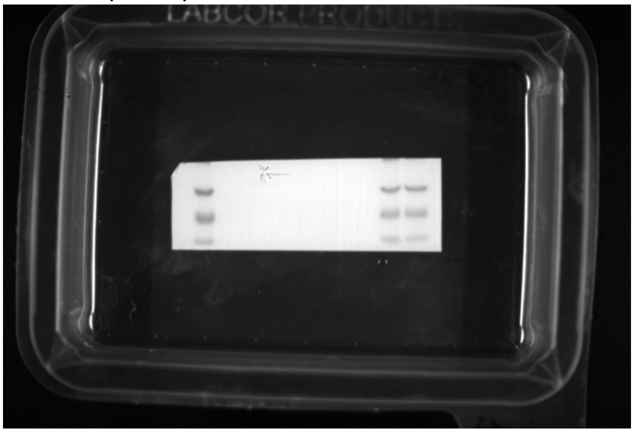

Actin

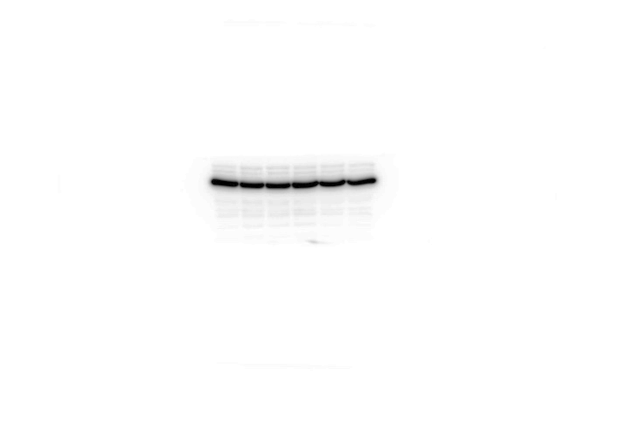

Pemigatinib resistance NCI-H1581 BR2 (Blot 1, FGFR2, FRS2, Actin)

Marker (FGFR2)

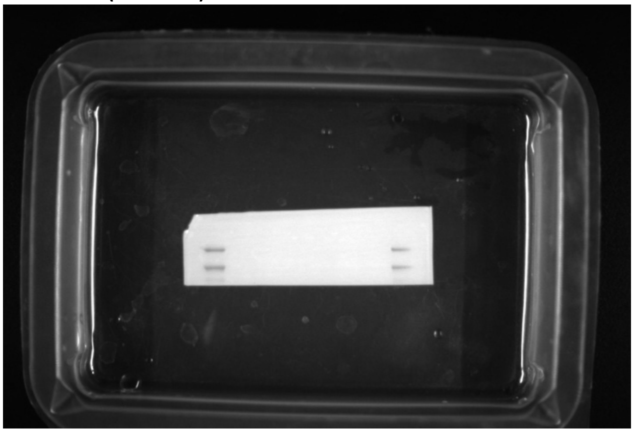

FGFR2

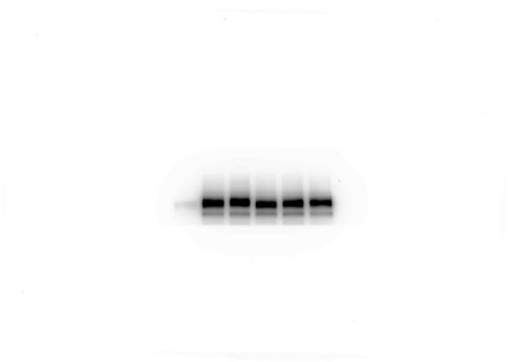

Marker (FRS2)

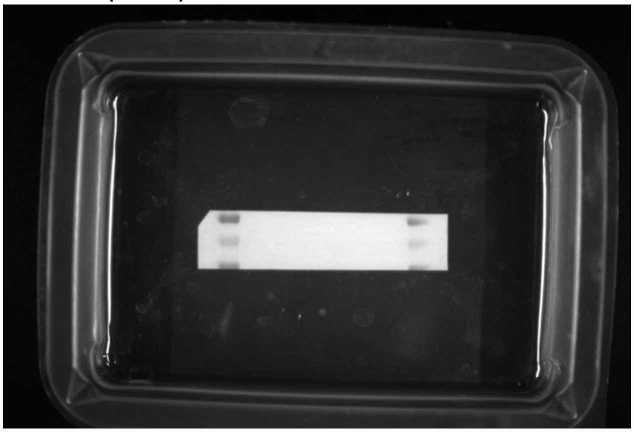

FRS2

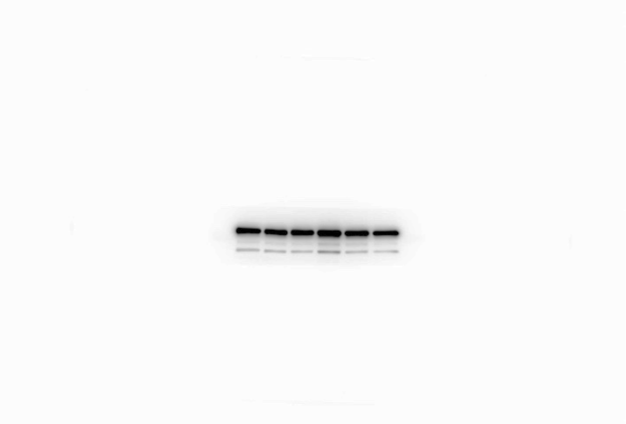

Marker (Actin)

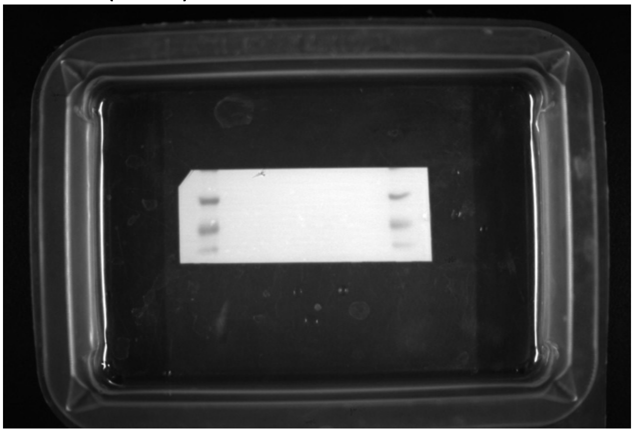

Actin

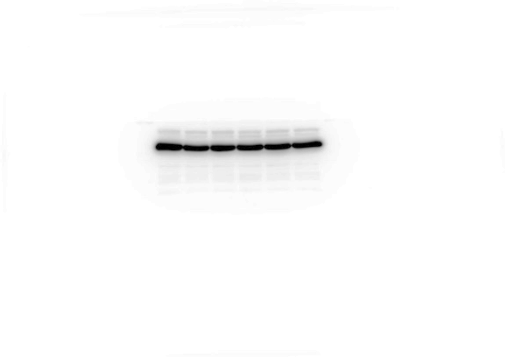

Pemigatinib resistance NCI-H1581 BR2 (Blot 2, pFGFR2, pFRS2, Actin)

Marker (pFGFR)

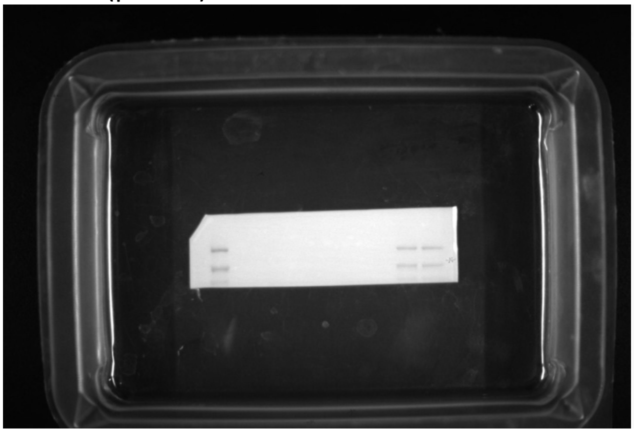

pFGFR

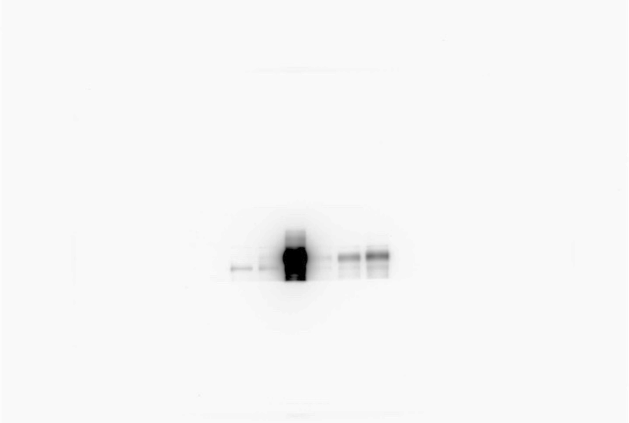

Marker (pFRS2)

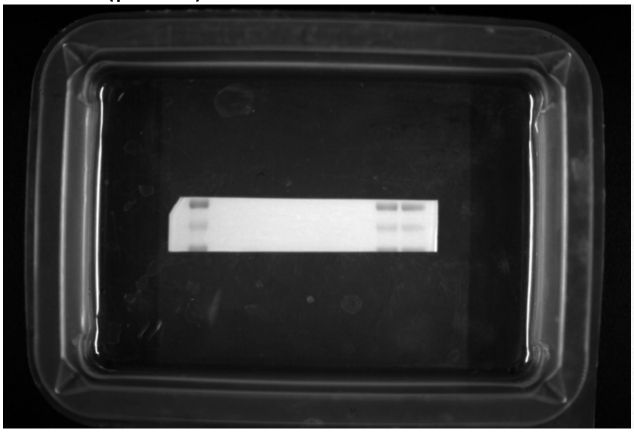

pFRS2

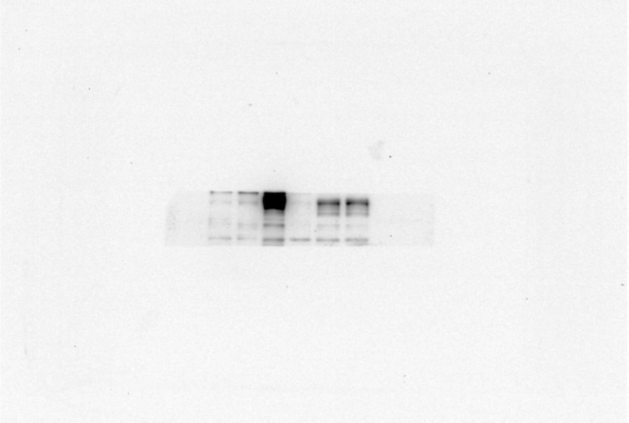

Marker (Actin)

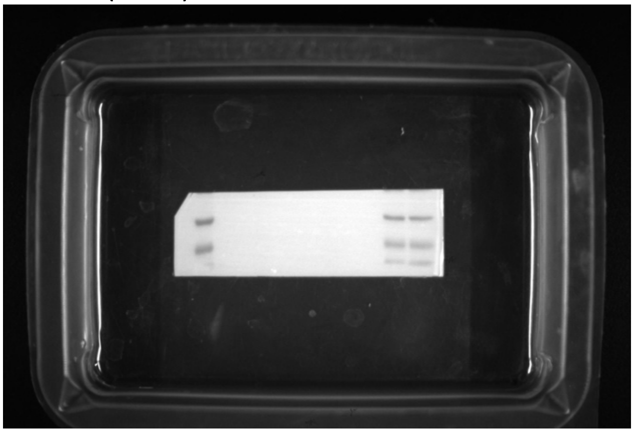

Actin

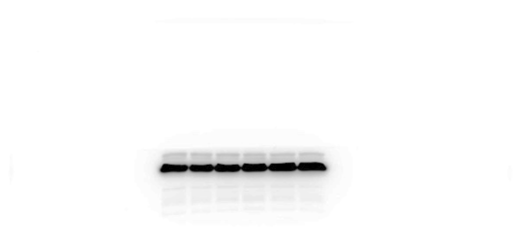

Pemigatinib resistance NCI-H1581 BR3 (Blot 1, FGFR2, FRS2, Actin)

Marker (FGFR2)

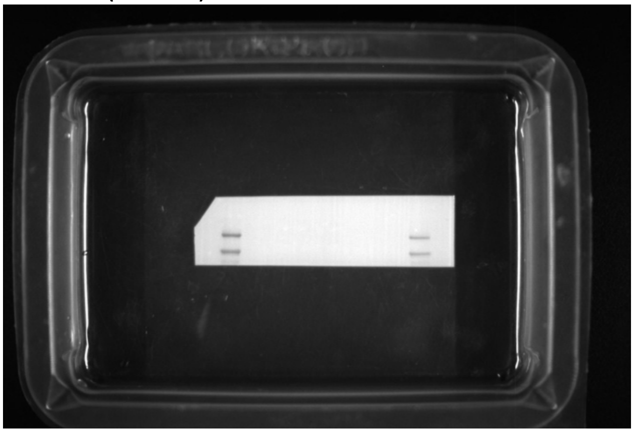

FGFR2

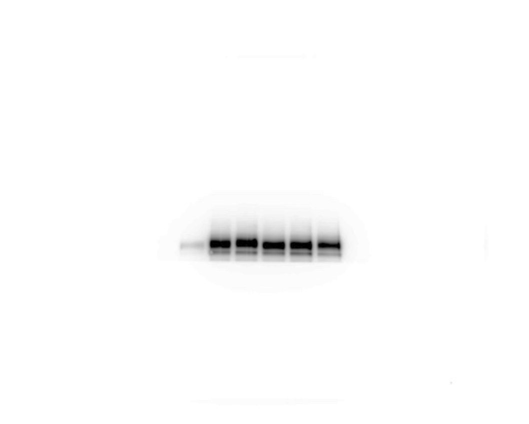

Marker (FRS2)

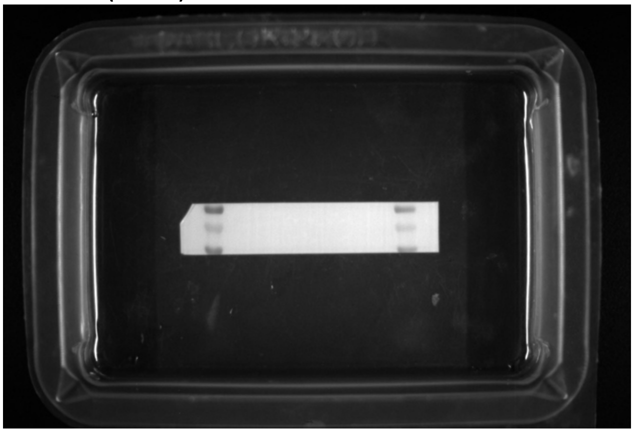

FRS2

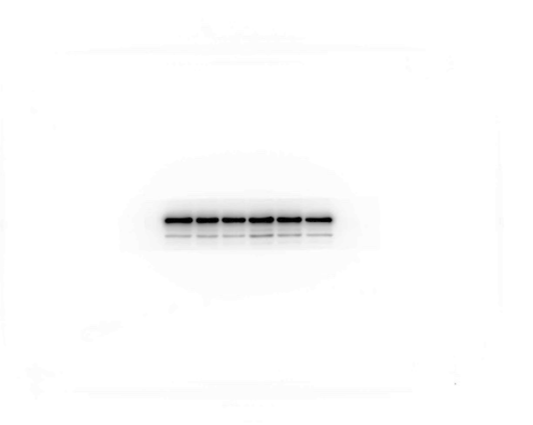

Marker (Actin)

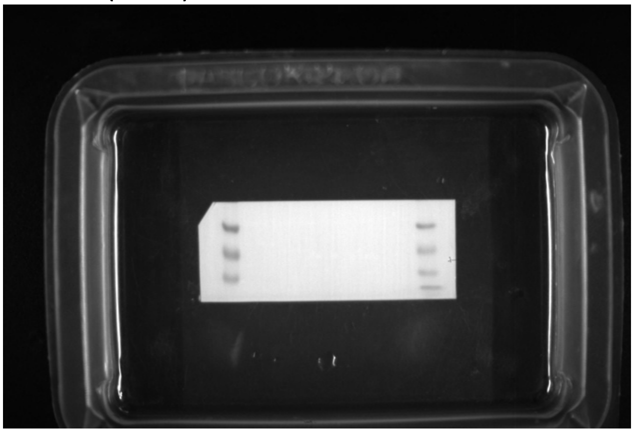

Actin

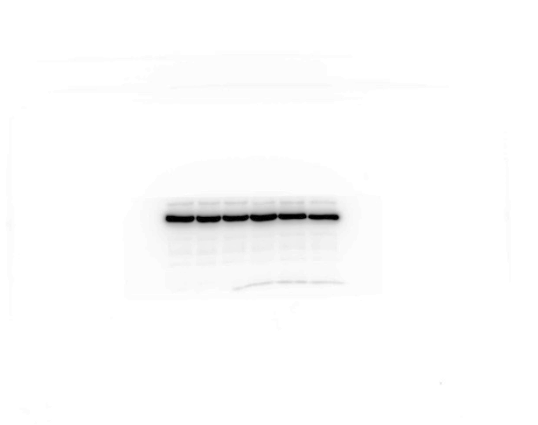

Pemigatinib resistance NCI-H1581 BR3 (Blot 2, pFGFR2, pFRS2, Actin)

Marker (pFGFR)

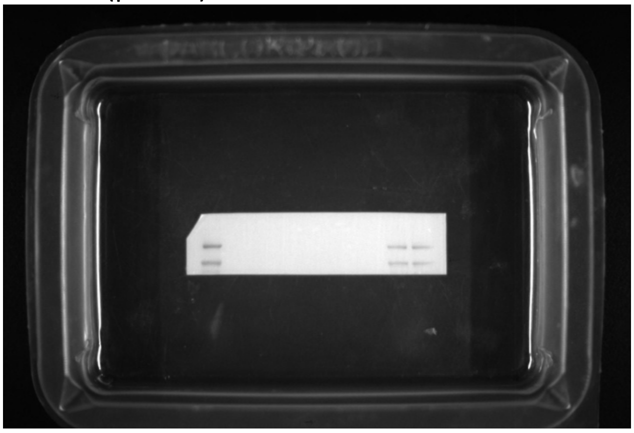

pFGFR

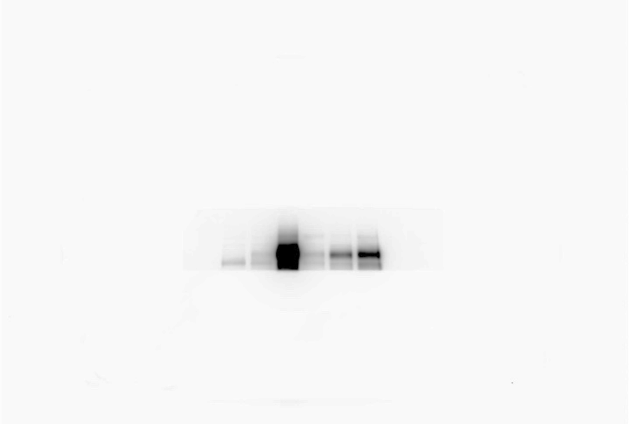

Marker (pFRS2)

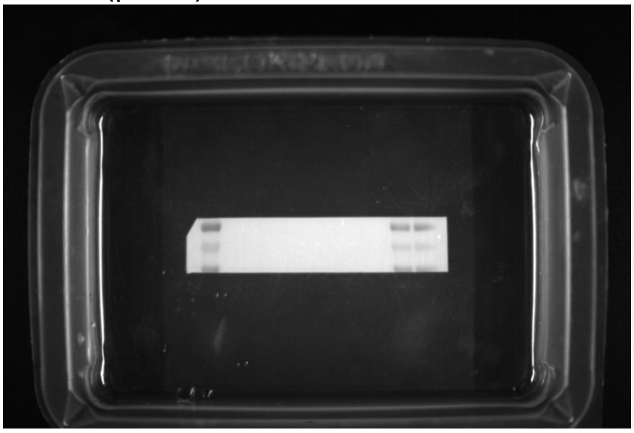

pFRS2

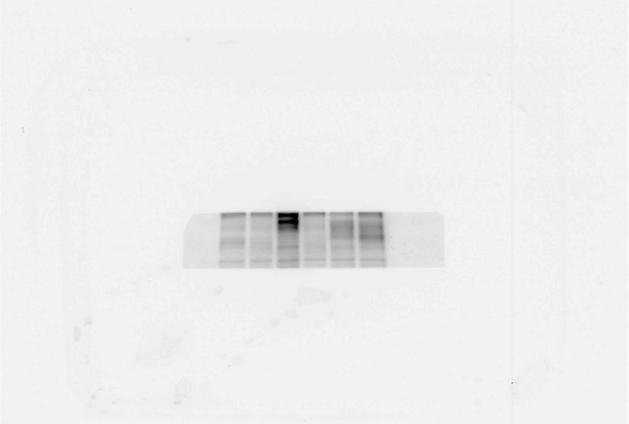

Marker (Actin)

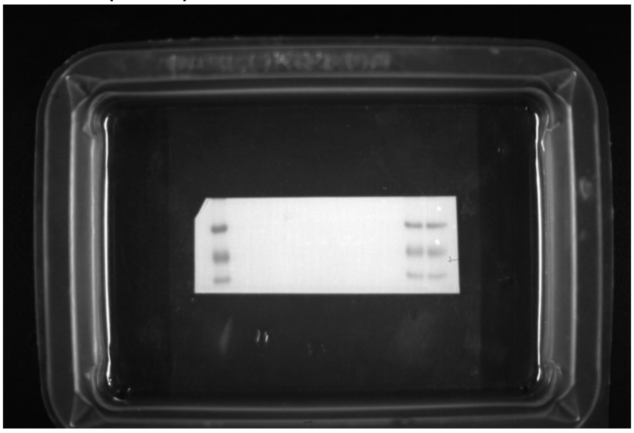

Actin

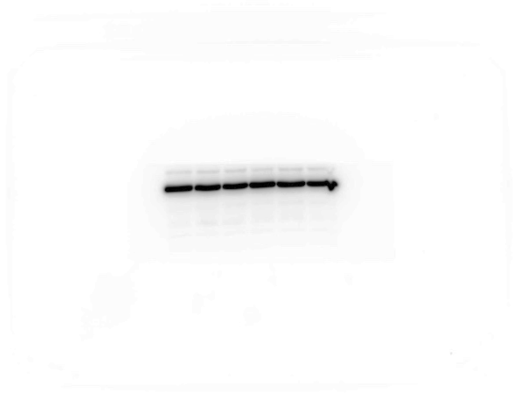

Pemigatinib resistance NCI-H1581 BR4 (Blot 1, FGFR2, FRS2, Actin)

Marker (FGFR2)

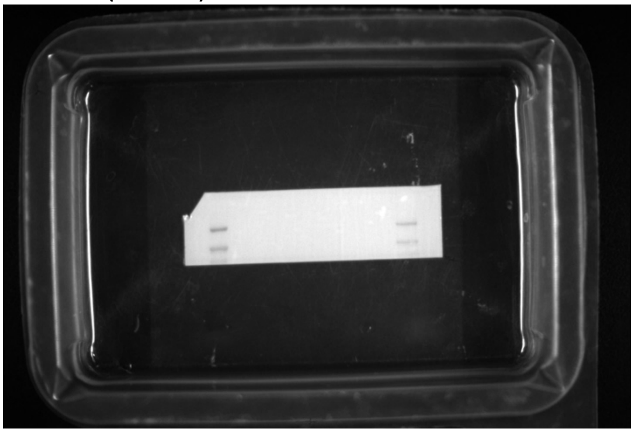

FGFR2

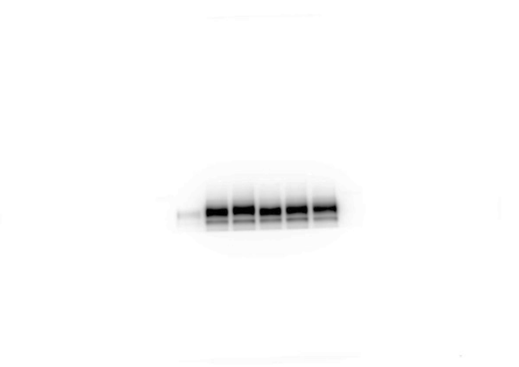

Marker (FRS2)

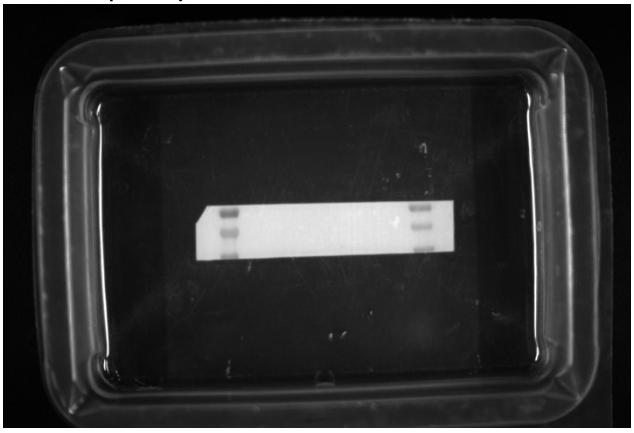

FRS2

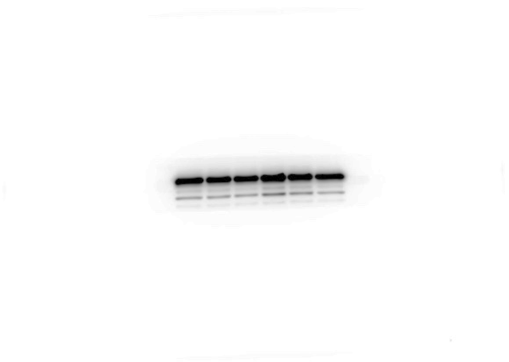

Marker (Actin)

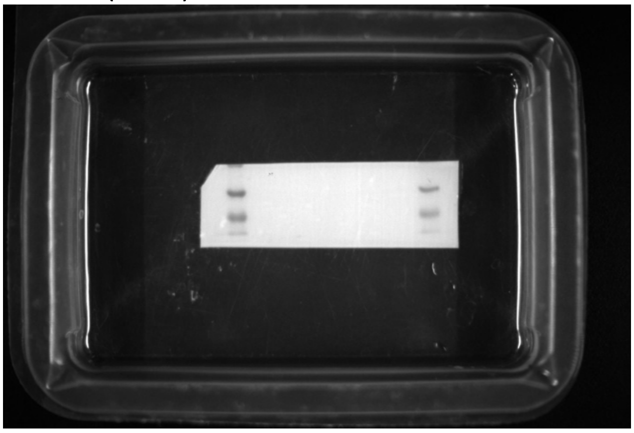

Actin

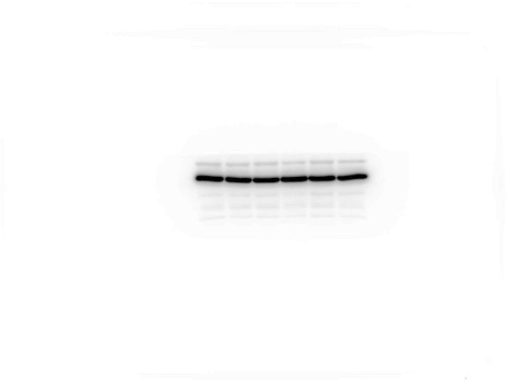

Pemigatinib resistance NCI-H1581 BR4 (Blot 2, pFGFR2, pFRS2, Actin)

Marker (pFGFR)

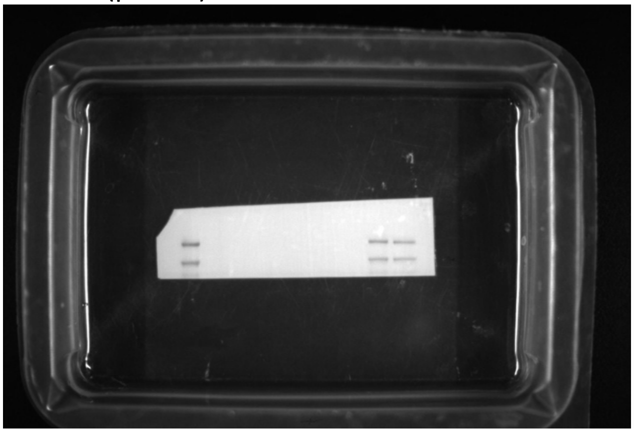

pFGFR

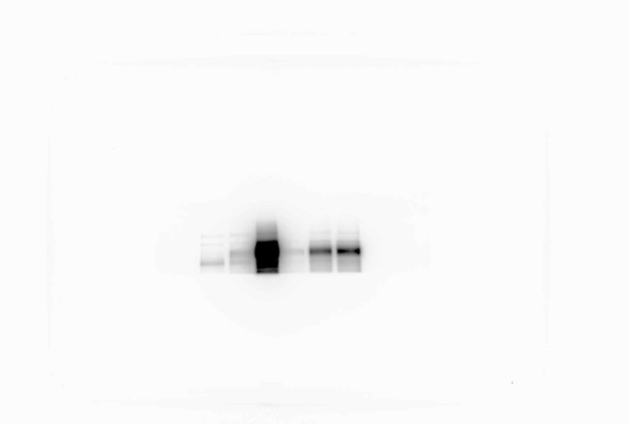

Marker (pFRS2)

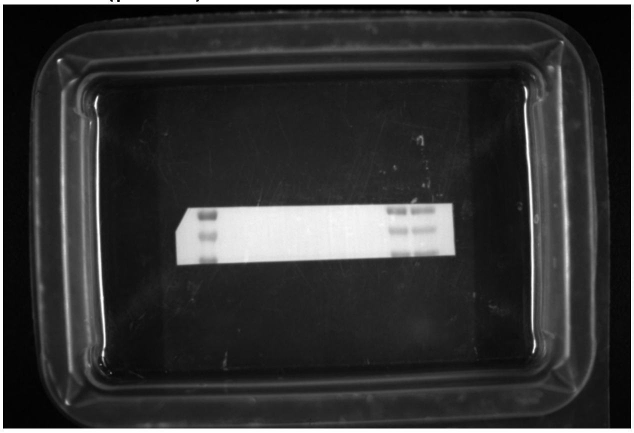

pFRS2

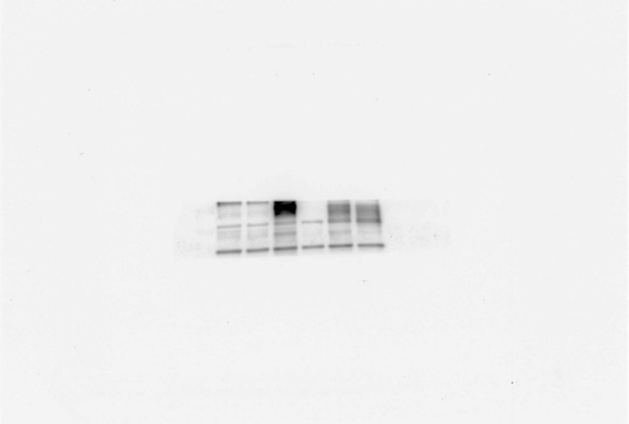

Marker (Actin)

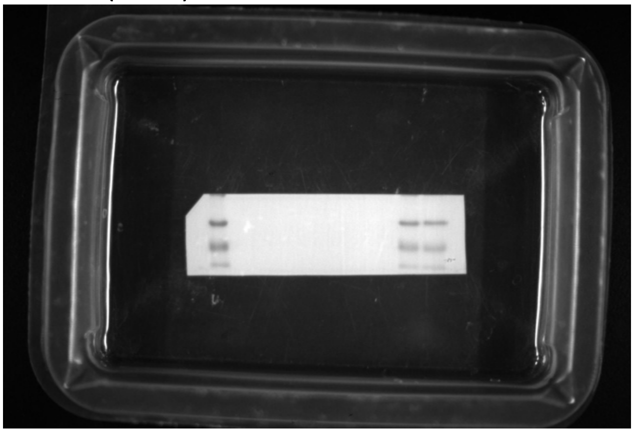

Actin

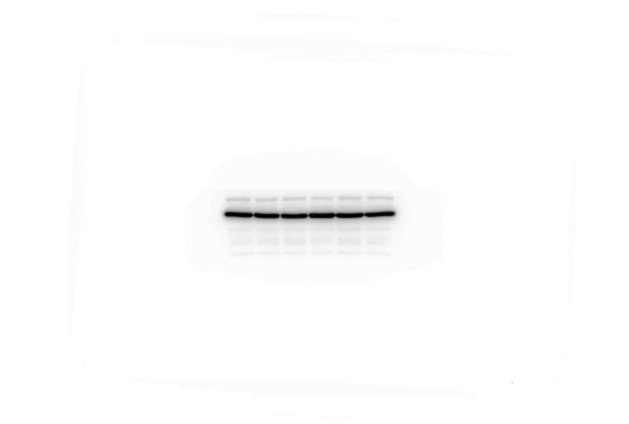

Futibatinib resistance = Figure 3f, right panel

Futibatinib resistance NCI-H1581 BR1 (Blot 1, FGFR2, FRS2, Actin)

Marker (FGFR2)

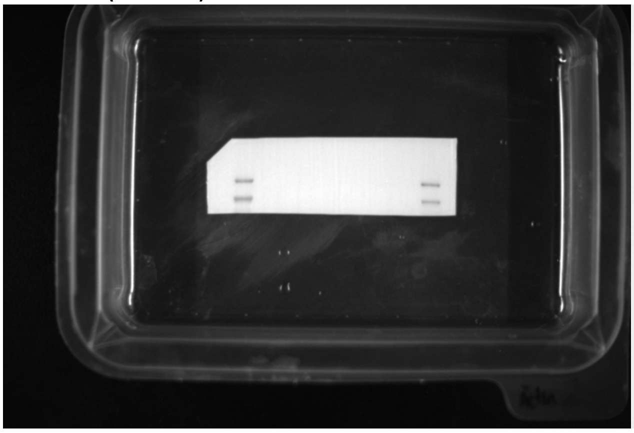

FGFR2

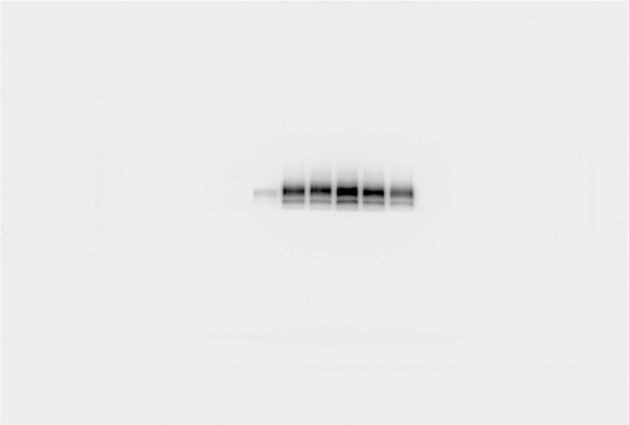

Marker (FRS2)

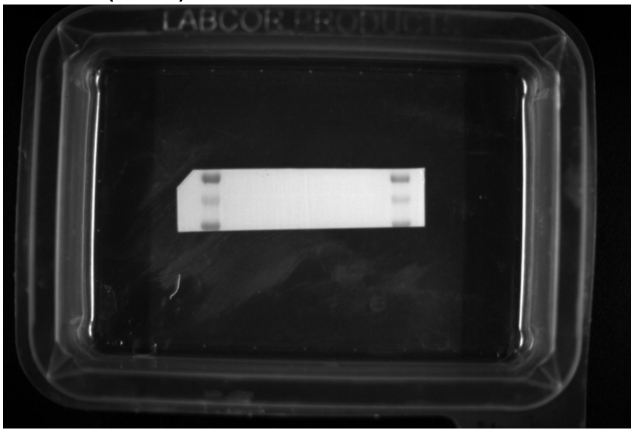

FRS2

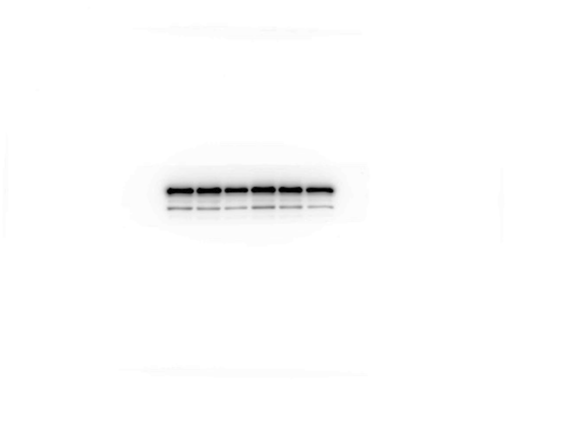

Marker (Actin)

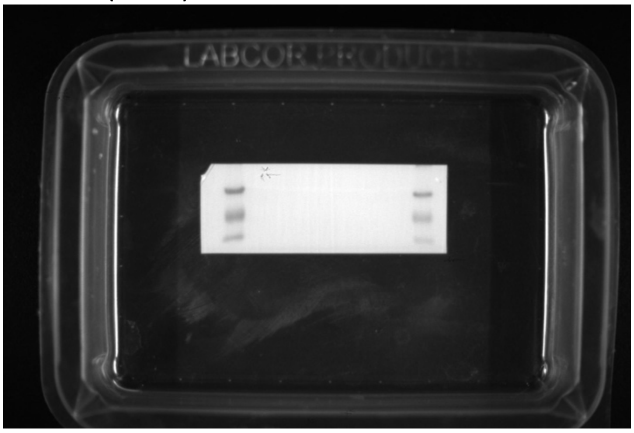

Actin

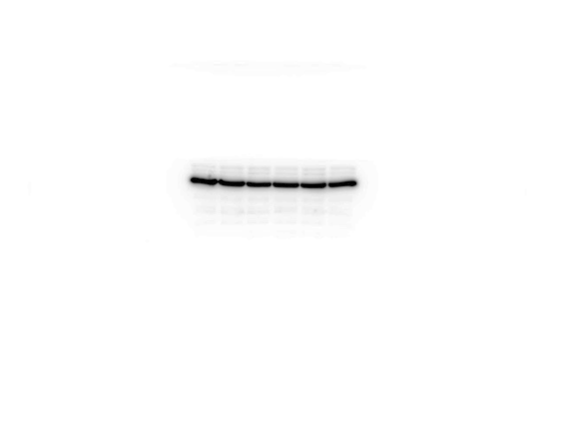

Futibatinib resistance NCI-H1581 BR1 (Blot 2, pFGFR2, pFRS2, Actin)

Marker (pFGFR)

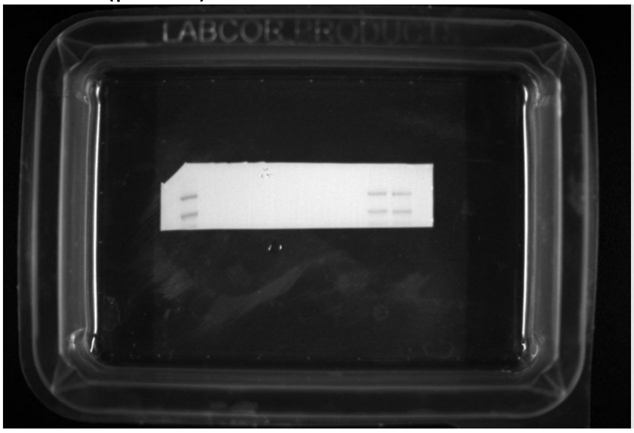

pFGFR

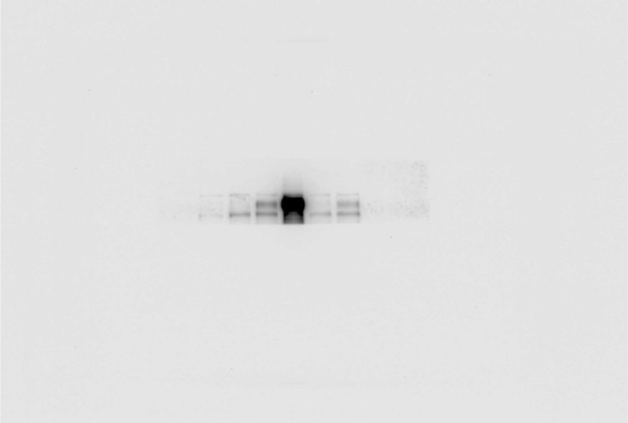

Marker (pFRS2)

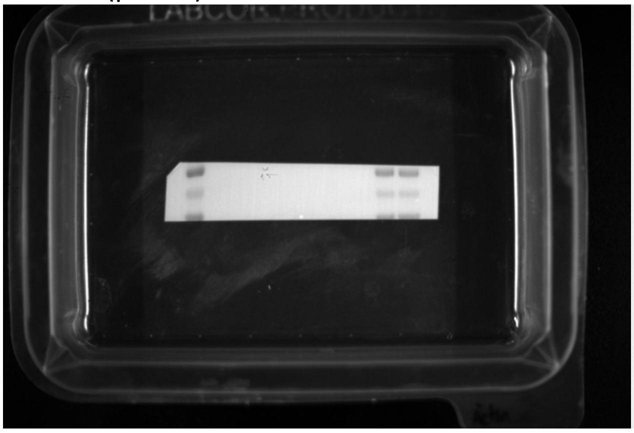

pFRS2

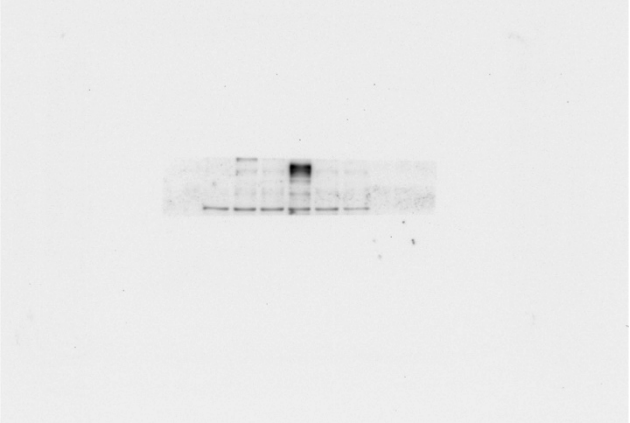

Marker (Actin)

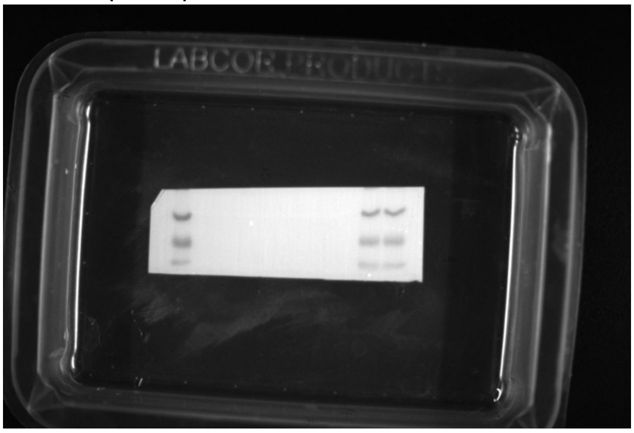

Actin

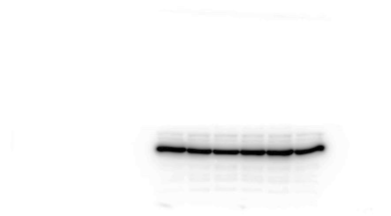

Futibatinib resistance NCI-H1581 BR2 (Blot 1, FGFR2, FRS2, Actin)

Marker (FGFR2)

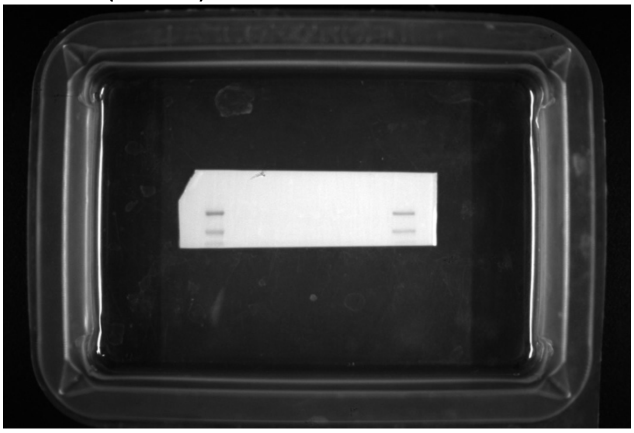

FGFR2

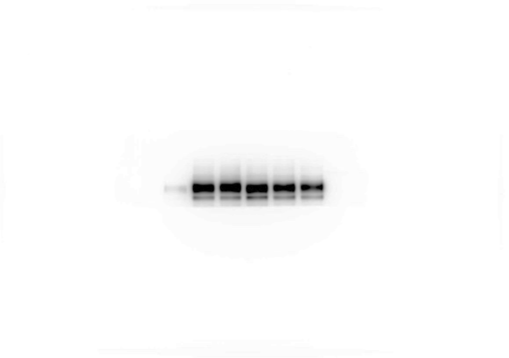

Marker (FRS2)

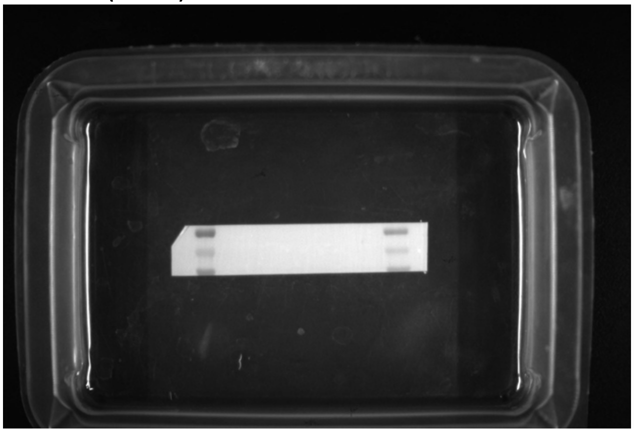

FRS2

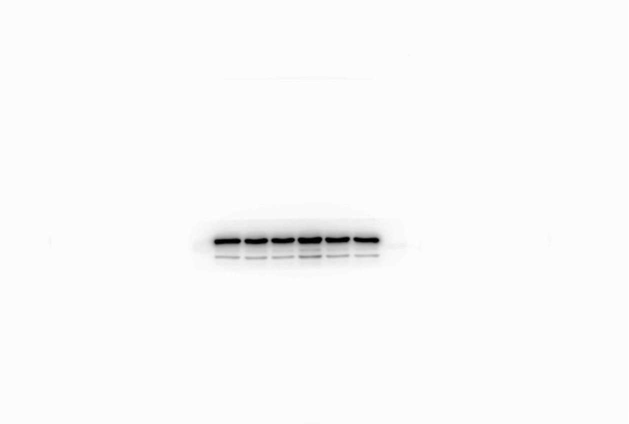

Marker (Actin)

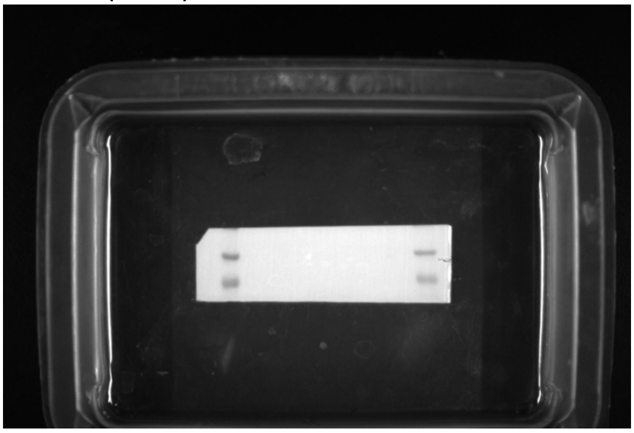

Actin

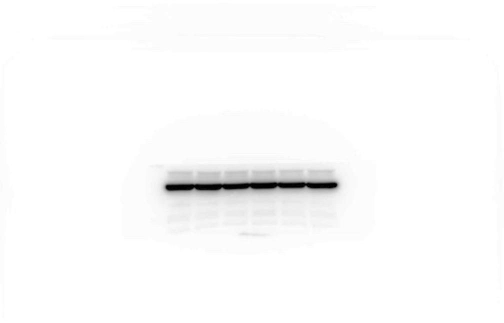

Futibatinib resistance NCI-H1581 BR2 (Blot 2, pFGFR2, pFRS2, Actin)

Marker (pFGFR)

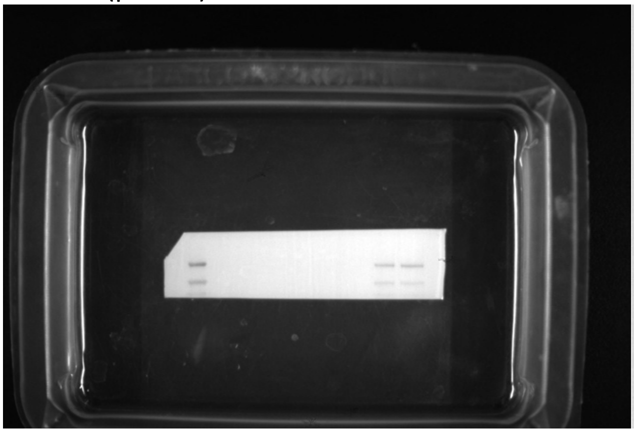

pFGFR

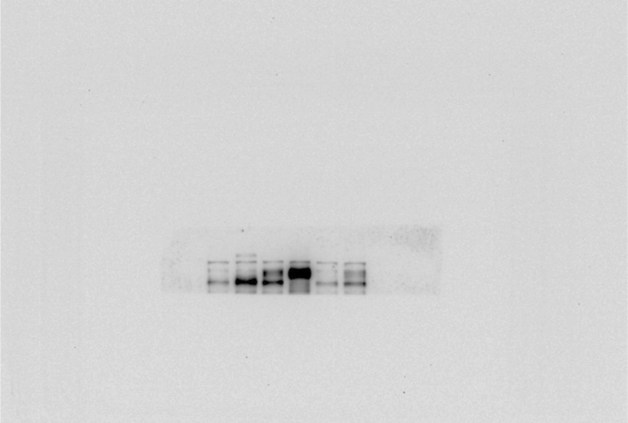

Marker (pFRS2)

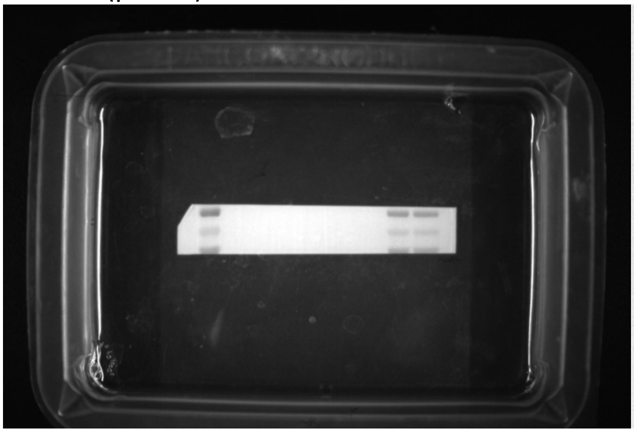

pFRS2

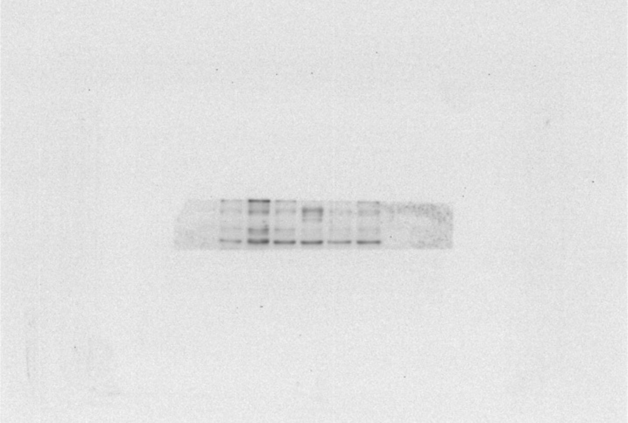

Marker (Actin)

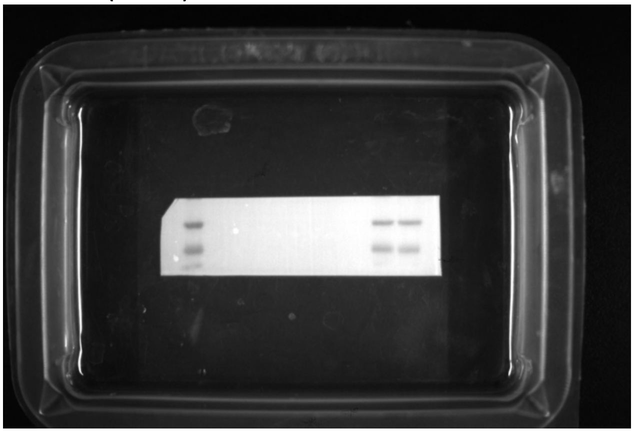

Actin

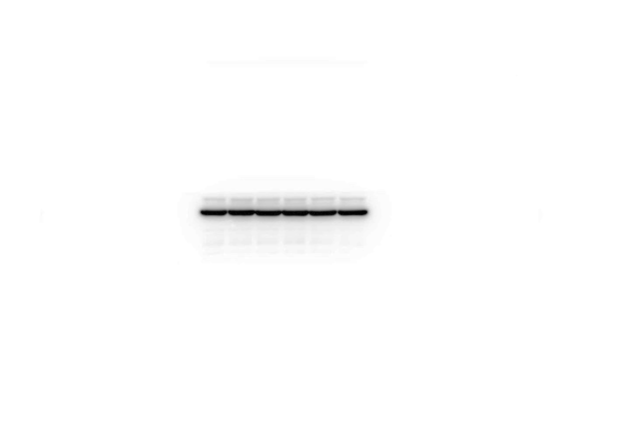

Futibatinib resistance NCI-H1581 BR3 (Blot 1, FGFR2, FRS2, Actin)

Marker (FGFR2)

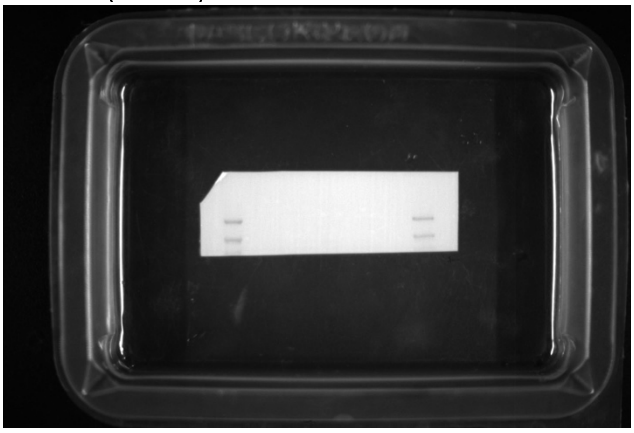

FGFR2

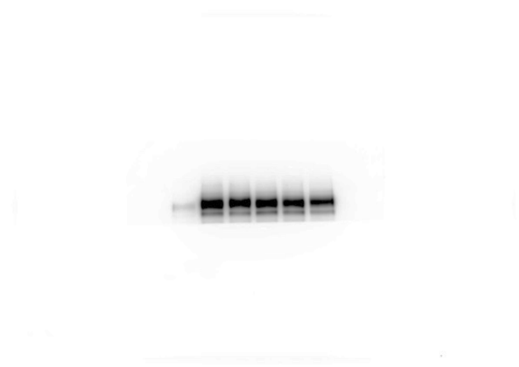

Marker (FRS2)

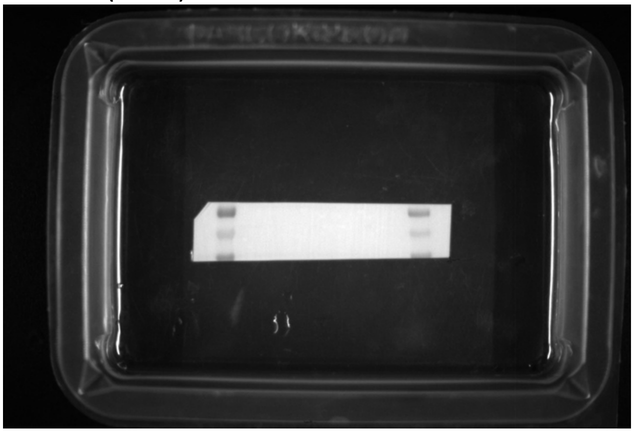

FRS2

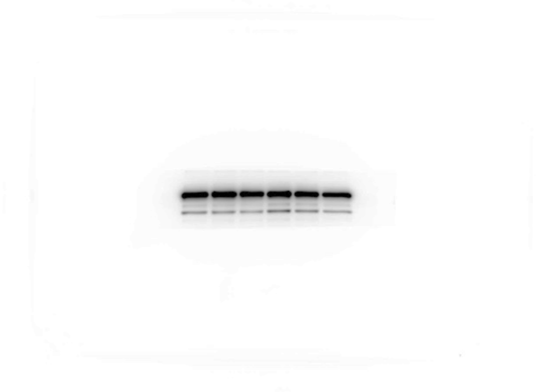

Marker (Actin)

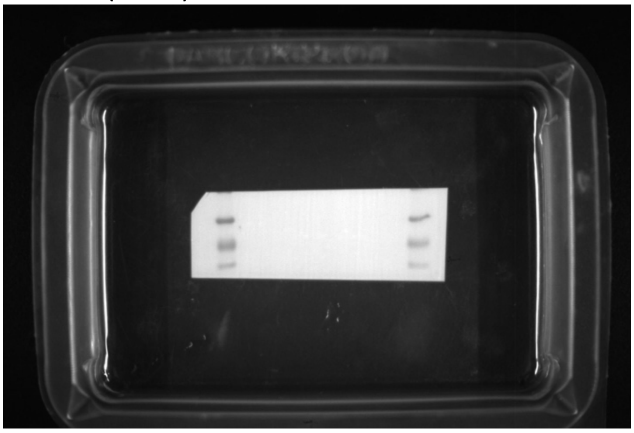

Actin

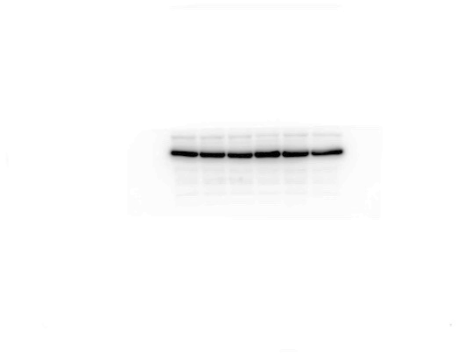

Futibatinib resistance NCI-H1581 BR3 (Blot 2, pFGFR2, pFRS2, Actin)

Marker (pFGFR)

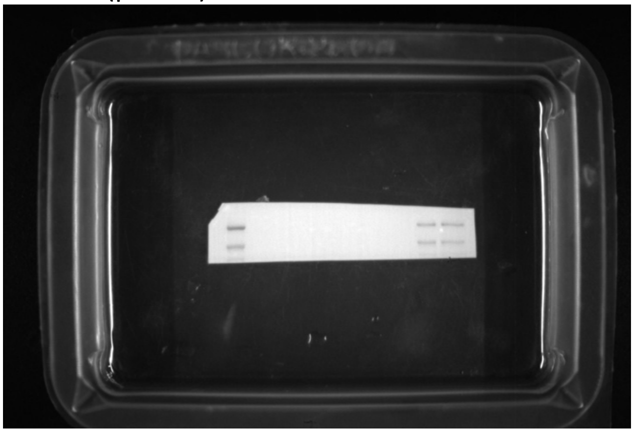

pFGFR

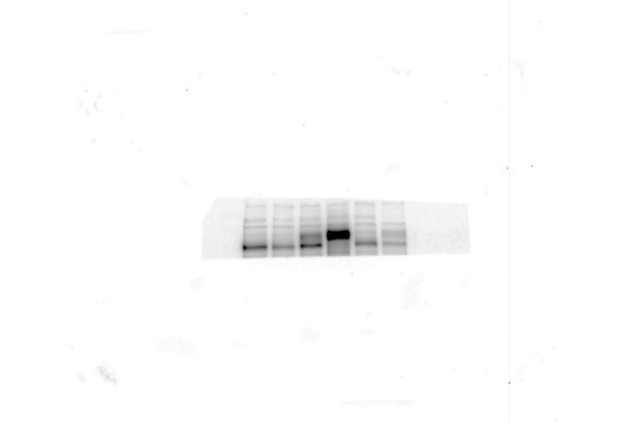

Marker (pFRS2)

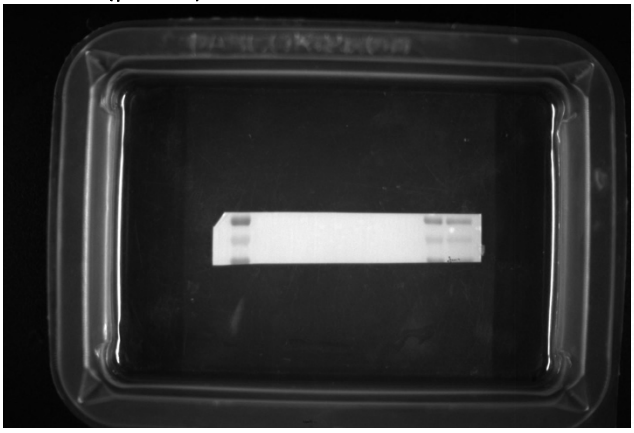

pFRS2

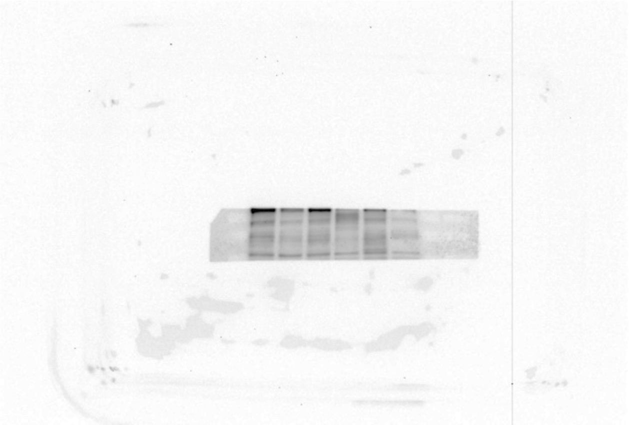

Marker (Actin)

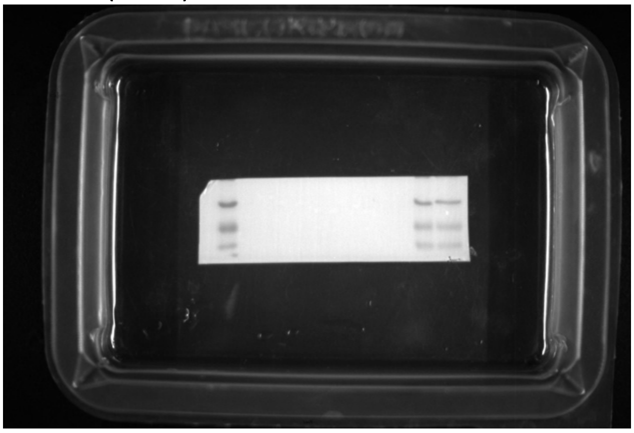

Actin

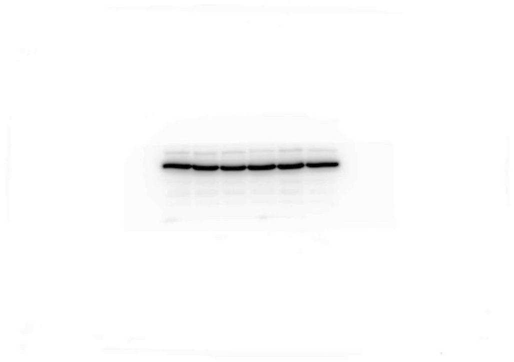

Futibatinib resistance NCI-H1581 BR4 (Blot 1, FGFR2, FRS2, Actin)

Marker (FGFR2)

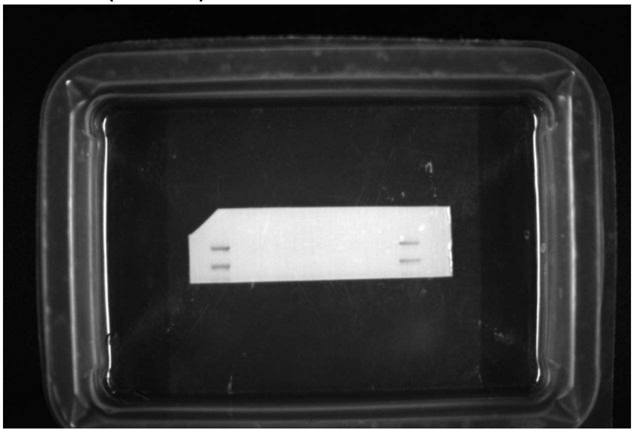

FGFR2

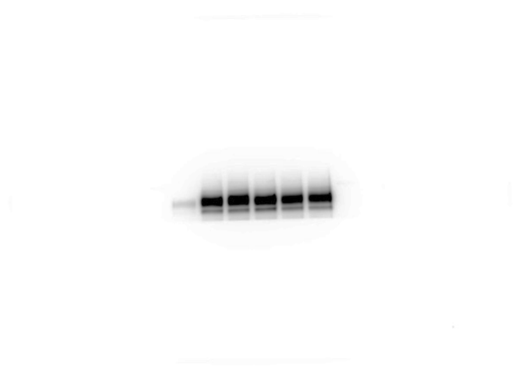

Marker (FRS2)

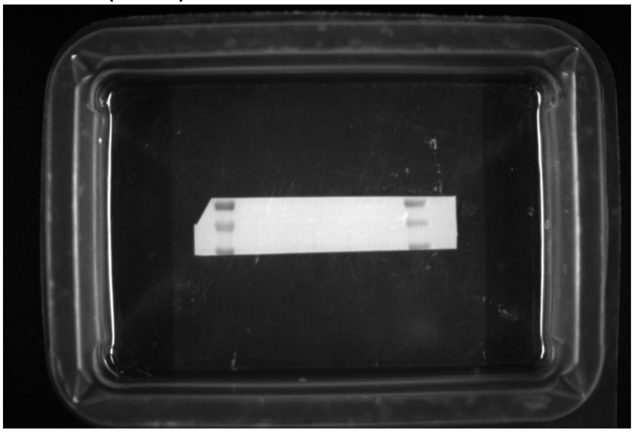

FRS2

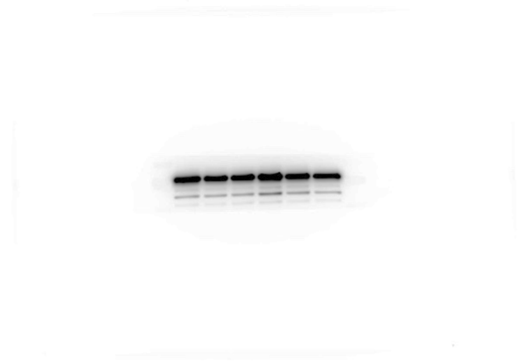

Marker (Actin)

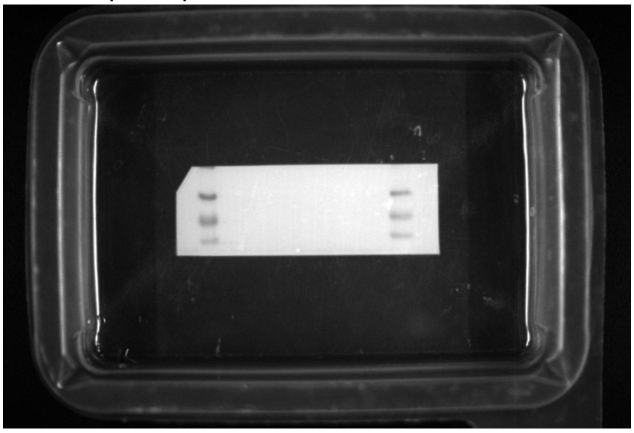

Actin

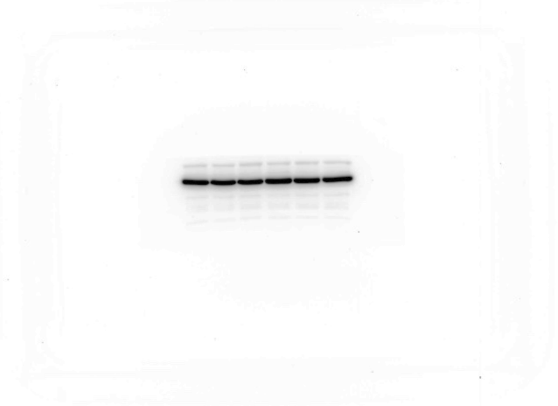

Futibatinib resistance NCI-H1581 BR4 (Blot 2, pFGFR2, pFRS2, Actin)

Marker (pFGFR)

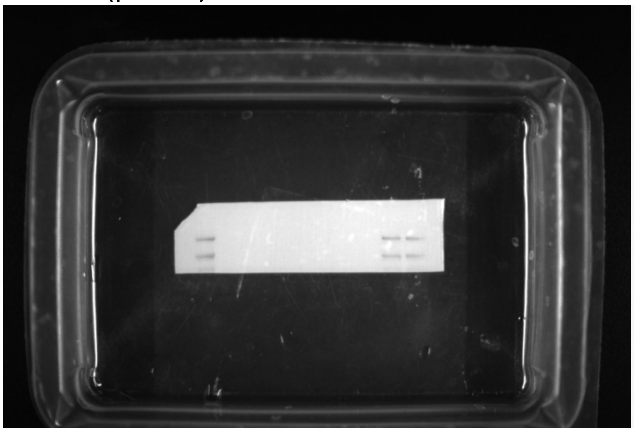

pFGFR

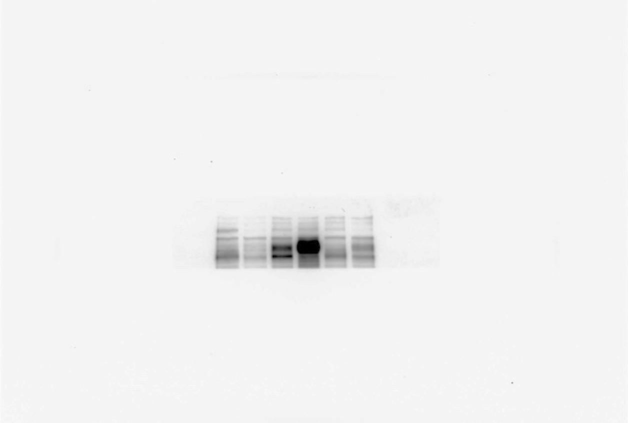

Marker (pFRS2)

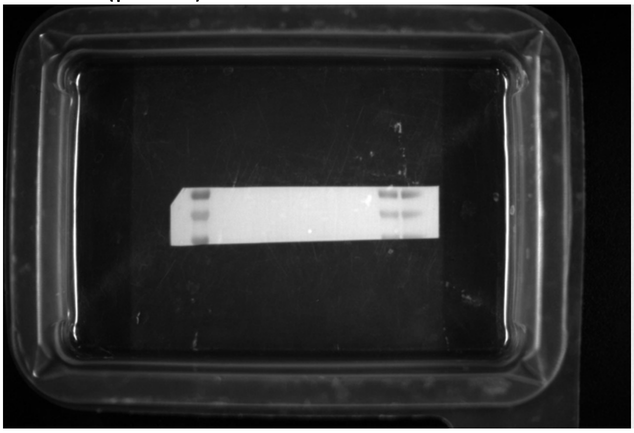

pFRS2

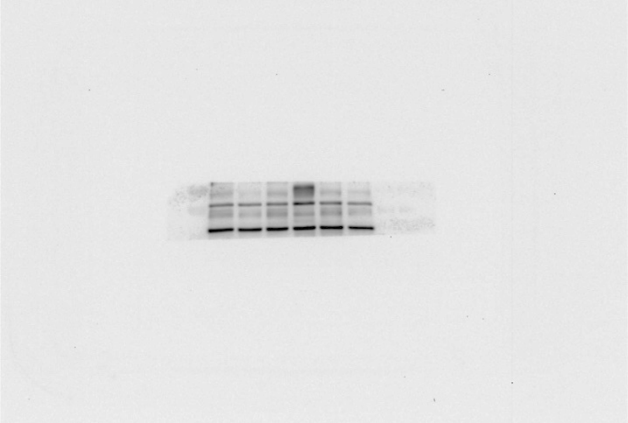

Marker (Actin)

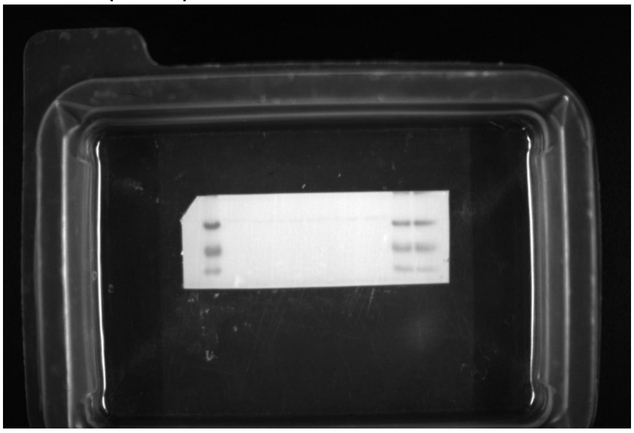

Actin

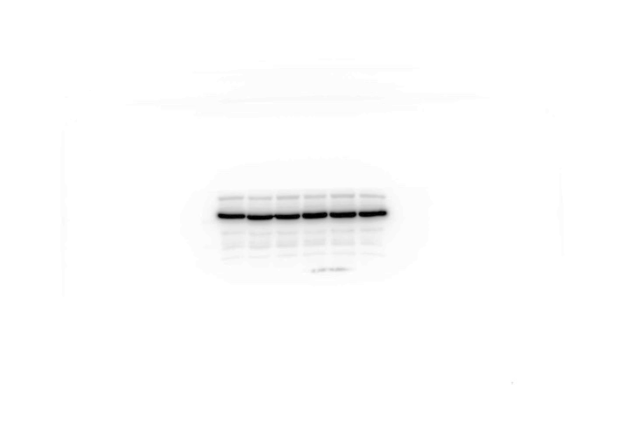

Supplement: Supplementary file 7 — Unprocessed western blots. [file 41588_2025_2431_MOESM7_ESM.pdf]
